# A Chemometric Analysis of Deep-Sea Natural Products

**DOI:** 10.3390/molecules24213942

**Published:** 2019-10-31

**Authors:** Lisa I. Pilkington

**Affiliations:** School of Chemical Sciences, The University of Auckland, Auckland 1010, New Zealand; lisa.pilkington@auckland.ac.nz; Tel.: +64-9-373-7599 (ext. 86776)

**Keywords:** deep-sea, natural products, *drug-like*, *lead-like*, known drug space, chemical space

## Abstract

Deep-sea natural products have been created by unique marine organisms that thrive in a challenging environment of extreme conditions for its inhabitants. In this study, 179 deep-sea natural products isolated from 2009 to 2013 were investigated by analysing their physicochemical properties that are important indicators of the ADMET (Absorption, Distribution, Metabolism, Excretion and Toxicity) profile of a compound. The study and analysis of these molecular descriptors and characteristics enabled the defining of these compounds in various chemical spaces, particularly as an indication of their *drug-likeness* and position in chemical space and is the first to be conducted to analyse deep-sea derived natural products. It was found that ~40% of all deep-sea natural products were *drug-like* and 2/3 were within Known Drug Space (KDS), highlighting the high *drug-likeness* of a significant proportion of deep-sea natural products, most of which have already been shown to have notable biological activities, that should be further investigated as potential therapeutics. Furthermore, this study was able to reveal the general structural differences between compounds from Animalia, Bacteria and Fungi organisms where it was observed that natural products from members of the Animalia kingdom are structurally more varied than compounds from bacteria and fungi. It was also noted that, in general, fungi-derived compounds occupy a more favourable position in *drug-like* chemical space and are a rich and promising source of biologically-active natural products for the purposes of drug development and therapeutic application.

## 1. Introduction

Natural products have provided plentiful and notable inspiration in the pursuit of modern drug therapies, with a number of therapeutics inspired by compounds of natural origins. A significant proportion (~40%) of existing drugs on the market are either natural products or derivatives thereof [1]. While historically almost all of these natural product-derived therapeutics are developed from compounds that are of terrestrial origins, there is an ever-increasing interest in the discovery and development of drugs from marine natural products [2,3,4]. This trend is largely due to the array of novel and unique chemical structural motifs that are being found in marine organisms. It has been shown that marine natural products have greater chemical novelty that their terrestrial counterparts and that ~70% of structural scaffolds found in marine natural products are only found in marine-based life [5]. Furthermore, it has been shown that marine natural products are generally more biologically potent than natural products of terrestrial origins [2,4,6]. While these biological activities are varied, greater than 50% of marine natural product bioactivities are anti-proliferative and cytotoxic activities [7,8]—most likely indicative of the chemical defence functions that these compounds often impart for the organism from which they are derived [9,10,11].

**Figure 1:**
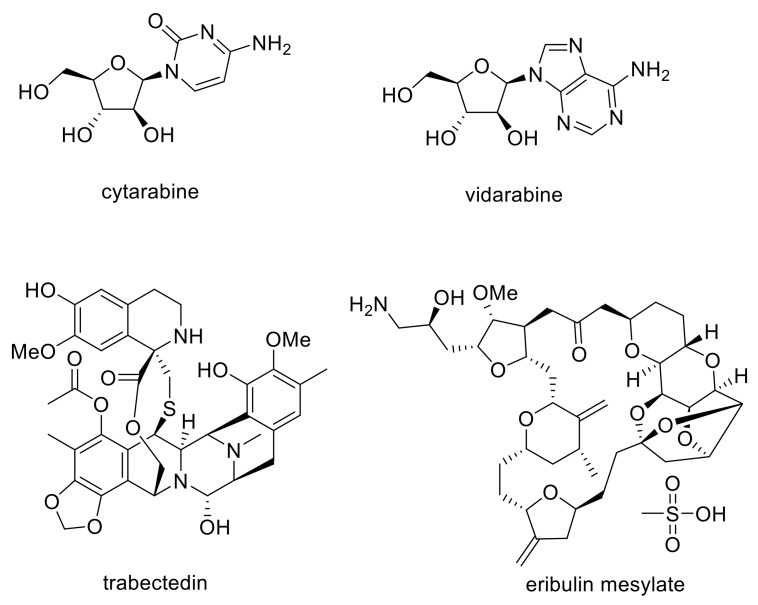
Structures of some marine-derived drugs that are or have been approved for clinical use.

The first FDA (U.S. Food and Drug Administration)-approved marine natural product-inspired drugs were anticancer agent (for acute lymphocytic leukemia, ALL; acute myeloid leukemia, AML; non-Hodgkins lymphoma and myelodysplasic syndrome, MDS) cytarabine and the antiviral vidarabine, in 1969 and 1976, respectively (Figure 1) [12]. Since then, a number of other drugs have been approved for use, including ziconotide (for severe chronic pain, in 2004), trabectedin (for cancers including soft tissue and ovarian cancers, in 2007 (Europe)/2015 (FDA), eribulin mesylate (for metatstatic breast cancer, in 2010) and brentuximab vedotin (for various cancers, in 2015 (Europe)/2011 (FDA). Furthermore, there are a number of marine-derived drug candidates currently in clinical trials [4,13]. These examples show the usage and impact of marine natural products as therapeutics and inspiration for their development—an area that is predicted to grow rapidly in the coming years [2,3,4].

The vast majority of known marine natural products are those that are isolated from shallow waters—of the ~30,000 known marine natural products [14,15,16], circa 2% are isolated from deep-sea waters [7,17]. This statistic contrasts significantly with that of the knowledge that 95% of the Earth’s seas are greater than 1000 m deep [17]. The main reason for this notable disparity is the limitations in our capabilities in sourcing marine organisms at depths below 50 m, that cannot be reached by scuba. In recent years, however, developments in technology have helped mitigate the difficulties in reaching greater depths, such as improved acoustic technology and collaborations with marine-based industries have allowed for the gaining of momentum in the discovery of deep-sea natural products.

The deep-sea offers up a challenging environment of extreme conditions for its inhabitants, including the absence of light (essentially no light penetrates below 250 m), low temperatures (~2 °C at 2000 m), low levels of oxygen and extremely high pressures (pressure increases by 1 atm every 10 m below sea level) [17]. As a result, the deep-sea is the habitat of an extraordinary array of unique marine species [17,18,19,20]. In order to survive, these organisms have had to make significant biochemical and physiological adaptations—these adaptations affect the metabolic pathways in these organisms, thereby giving rise to natural products with novel structural motifs [21,22].

As stated above, marine natural products are known to be potent molecules with a broad range of biological activities. Deep-sea natural products are no exception to this rule—there has been reports of very high hit rates from the screening of deep-sea organisms in biological assays, particularly for their anti-proliferative activities [23,24]. It has been reported that ~75% of deep-sea natural products possess notable biological activity, with greater than 50% of compounds displaying anti-proliferative activity against human cancer cell lines [7].

Although the most imperative property of a potential drug or lead compounds is its biological activity, it is extremely important for a compound to have acceptable ADMET (Absorption, Distribution, Metabolism, Excretion and Toxicity) profiles as these characteristics are critical to evaluate the likelihood of a given compound of being an effective drug lead [25]. The calculation and assessment of the physicochemical properties of a compound enables the assessment of the ADMET profile of a compound, whereby these molecular descriptors can be compared to existing and verified benchmarks that define *lead-like*, *drug-like* and known drug chemical spaces [26,27].

*Lead-like* compounds are compounds that are those that are suitable to undergo further modification before use as therapeutics [28,29]—therefore, to be considered as *lead-like*, compounds need to be small, less-complex compounds with low molecular weights and lipophilicity (LogP) as these properties will typically increase during the drug-optimisation process [30]. The Lipinski’s rule of five is a set of parameters that define a chemical space for compounds that are capable of being orally-absorbed [31,32], and is the set of values that define *drug-like* chemical space. Lastly, KDS (known drug space) is a set of criteria that includes all small organic compounds that have been assessed in human clinical trials and were/are subsequently in therapeutic use [33]. The upper limits for each chemical space referred to in this study are provided (Table 1).

We wished to place recently-isolated deep-sea marine natural products in these chemical spaces to identify both overall trends in the characteristics of these novel natural products, as well as to identify any differences within or between compounds according to the type of organism from which they were isolated and particular compounds of interest and their potential targets. Doing so would also establish this methodology as a useful approach to investigating and assessing drugs. Presented herein is the result of the investigation into the physicochemical properties of deep-sea marine natural products, to establish if these compounds are *lead-like*/*drug-like*, thereby exhibiting potential to be or act as leads in the development of future therapeutics. This is the first such study to be conducted to analyse deep-sea derived natural products.

## 2. Results and Discussion

All of the compounds in the most recent available review of deep-sea natural products, by Skropeta and Wei, were included in this study [17]. This set of compounds was chosen as it was deemed that the most recently reported natural products be those of most interest to those looking for compounds for future development or research. These compounds were those that had been isolated from 2009 to 2013 from deep-water (50–5000 m). The term deep sea is variable, however for this study, it has been defined as commencing at 50 m which is beyond the limits of recreational SCUBA (Self-Contained Underwater Breathing Apparatus) diving. The compound details (name, origin and CAS number) of the 179 compounds included in this study are given in the Supplementary Information (Table S1, while the review by Skropeta and Wei lists 188 compounds, nine of these compounds were not able to be appropriately modelled).

**Figure 2:**
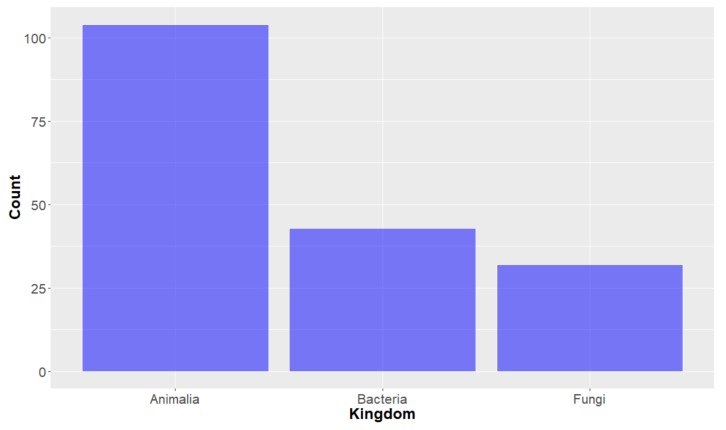
Number of natural products isolated from deep-sea marine organisms, by Kingdom.

**Figure 3:**
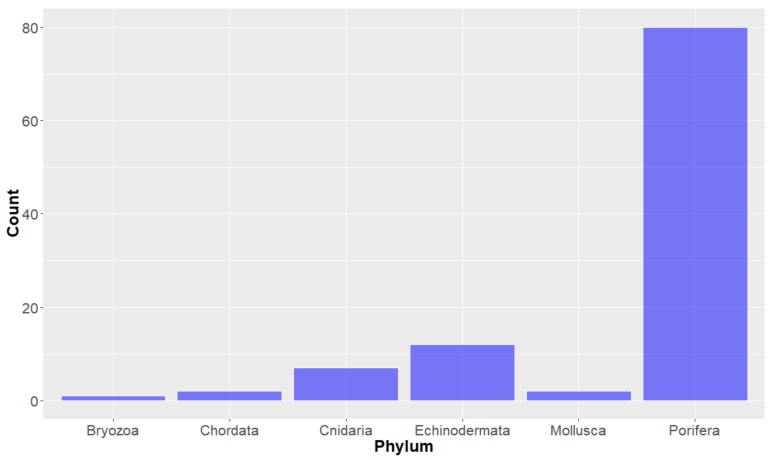
Number of natural products isolated from the Animalia Kingdom, by Phylum.

**Figure 4:**
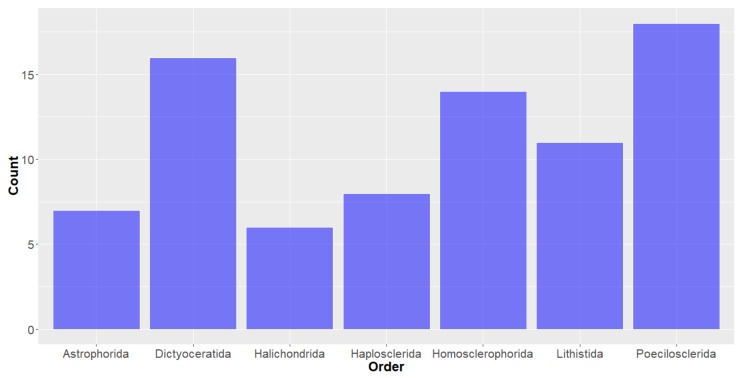
Number of natural products isolated from deep-sea marine organisms of the Porifera Phylum in the Animal Kingdom, by order.

**Figure 5:**
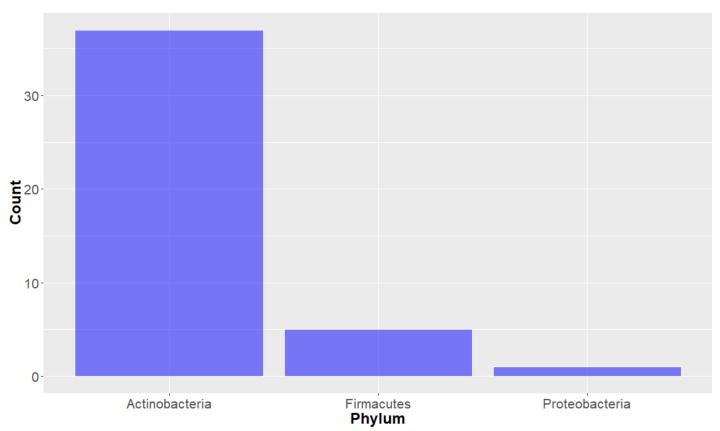
Number of natural products isolated from deep-sea marine bacteria, by Phylum.

**Deep-Sea Organisms.** For the purposes of this study, organisms were first classified using the taxonomic system outlined by Ruggiero et al. [34] and looking at the taxonomy of the deep-sea marine species from which the studied compounds originated, it can be seen that the organisms of the Animalia Kingdom have proffered the most of the deep-sea marine natural products in the years studied (104 out of the 179). Bacteria and Fungi kingdom-derived products are also represented, with 43 and 32 compounds, respectively (Figure 2). Of the natural products derived from the Animalia kingdom, the vast majority (greater than 75%) were from the Porifera (Sea sponge) Phylum (Figure 3), within which many orders were represented (Figure 4). It should be noted that there are some instances where natural products obtained from Porifera have been later determined to originate from a microorganism present in the sponge; [35] for the purpose of this study, compounds are considered to have originated from the sponge unless a microorganism has been deemed to be the source. The bacterium studied all come from three Phylum (actinobacteria, firmacutes and proteobacteria, Figure 5), while only one Phylum of fungi, ascomycota have been shown to exist in deep seas and provide marine natural products of interest, in the years studied.

**Molecular descriptors.** Utilising the described methods, a range of molecular descriptors were calculated for each of the 179 compounds studied. Molecular weight, lipophilicty (LogP), the number of hydrogen bond donors, hydrogen bond acceptors and rotatable bonds and polar surface area (PSA) have been extensively used in the assessment of a molecules’ suitability to be considered as a drug [36]. Polarisability and water solubility (LogS) have been used less extensively in the assessment of *drug-like* properties, however their association with desirable characteristics for therapeutics has recently led them to being increasingly studied [26,27,37,38,39].

To analyse the molecular descriptors, summary statistics—the mean, median and standard deviation for each of these variables, both overall, and also classified by the Kingdom of the marine organism from which the natural products were derived, were calculated and are shown in Table 2.

**Figure 6:**
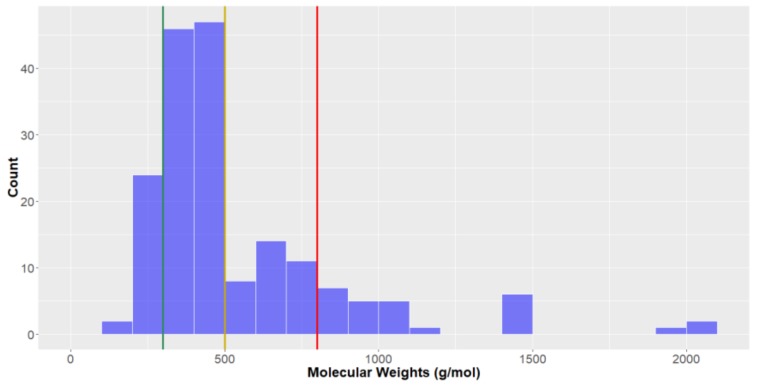
The statistical distribution of the molecular weight of all analysed compounds (green = 300 g mol^−^^1^, compounds <300 g mol^−1^ are in the *lead-like* space; yellow = 500 g mol^−1^, compounds <500 g mol^−1^ are in the *drug-like* space; red= 800 g mol^−1^, compounds <800 g mol^−1^are in the KDS. Total number of compounds = 179.

*Molecular weight.* Analysing the molecular weights of the studied compounds, it can be seen that they are right-skewed (Figure 6) with an average of 545.6 g mol^−1^ and median of 436.5 g mol^−1^. While the majority of the studied compounds have a molecular weight below 1000 g mol^−1^, there were some large compounds with high molecular weights, contributing to a large overall standard deviation (327.5 g mol^−1^). There were notable differences between the average molecular weights of natural products from the different kingdoms—those from the Animalia Kingdom were largest on average (611.8 ± 390.4 g mol^−1^), then bacteria (491.2 ± 196.4 g mol^−1^) and fungi (403.4 ± 132.7 g mol^−1^) natural products. To be considered *lead-like* in terms of molecular weight, compounds should be <300 g mol^−1^; only approximately 15% of deep-sea natural products have sufficiently low molecular weights to be classified as such. In contrast, 2/3 of the natural products are *drug-like* (<500 g mol^−1^) and 85% in KDS.

**Figure 7:**
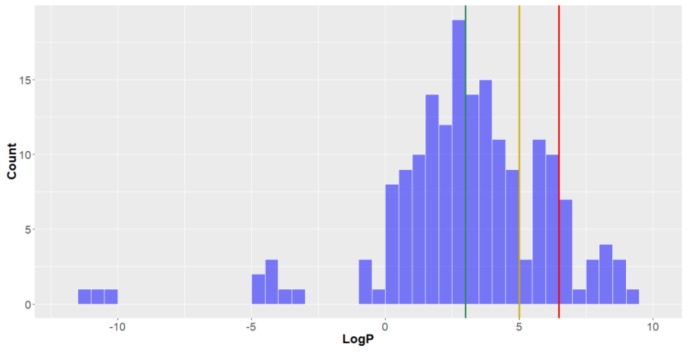
The statistical distribution of the octanol—water partition coefficient (LogP) of all analysed compounds (green = 3, compounds < 3 are in the *lead-like* space; yellow = 5, compounds < 5 are in the *drug-like* space; red = 6.5, compounds < 6.5 are in the KDS. Total number of compounds = 179.

*The octanol—water partition coefficient (LogP)*. Looking at the distribution of the lipophilicities (LogP, the octanol-water partition coefficient) of the studied compounds it can be seen that, with the exception of a small subset of compounds with very low values for this parameter, they are roughly normally distributed (mean = 3.1, standard deviation = 3.2, Figure 7). Approximately half of all the compounds have LogP < 3, qualifying them to be considered lead-like. Furthermore, 75% of the compounds are drug-like and ~90% have a lipophilicity low enough to exist in KDS. The deep-sea natural products from the Animalia Kingdom had the highest lipophilicities, while those from Bacteria and Fungi were lower (mean = 3.7 for Animalia, vs. 2.2 and 2.3 for Bacteria and Fungi, respectively), thereby demonstrating a high affinity for non-aqueous systems and a lower degree of hydrophilicity for deep-sea natural products isolated from microorganisms.

**Figure 8:**
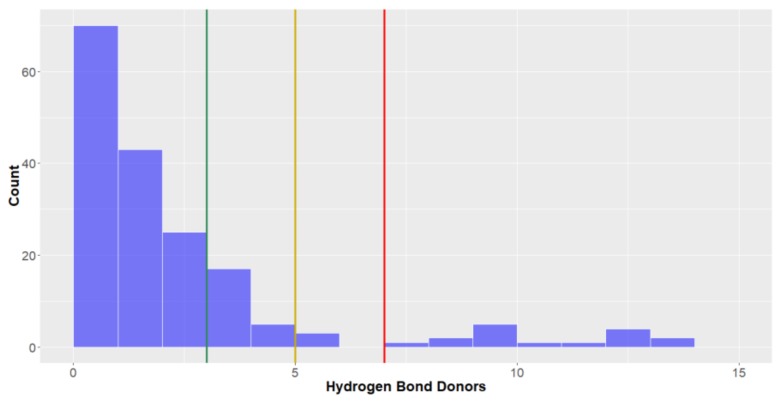
The statistical distribution of the hydrogen bond donors of all analysed compounds (green = 3, compounds < 3 are in the *lead-like* space; yellow = 5, compounds < 5 are in the *drug-like* space; red = 7, compounds < 7 are in the KDS. Total number of compounds = 179.

*Hydrogen bond donors and acceptors.* When analysing the number of hydrogen bond donors within a molecule, they should be lower than three, five and seven to be considered to be in *lead-like*, *drug-like* space and KDS, respectively. Of all the molecular descriptors analysed in this study, this was the parameter that was most frequently met for inclusion into the various chemical spaces—the compounds in this study conform reasonably well with the three aforementioned definitions used for the chemical spaces (mean = 2.8, standard deviation = 2.0, Figure 8, Table 2) with most compounds having three or less hydrogen bond donors (77.1%), allowing them to be classified in *lead-like* space.

Similar to the inter-kingdom trends seen for molecular weights of the molecules, it can be seen that natural products isolated from Animalia Kingdom organisms exhibited the highest number of hydrogen bond donors (mean = 3.2), closely followed by those isolated from Bacteria (mean = 2.7). Natural products from deep-sea fungi had a notably lower mean hydrogen bond donors, averaging less than two in each molecule.

**Figure 9:**
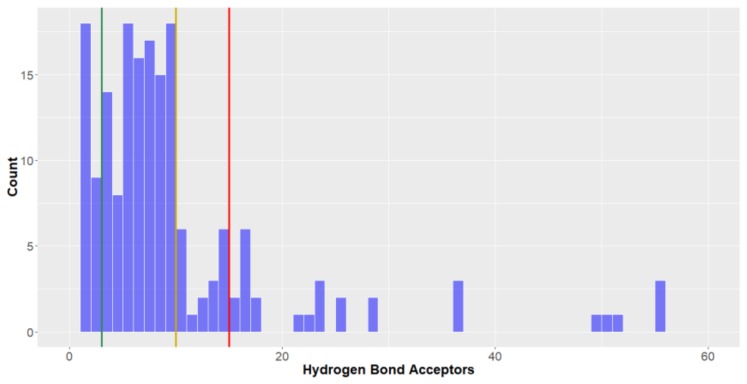
The statistical distribution of the hydrogen bond acceptors of all analysed compounds (green = 3, compounds < 3 are in the *lead-like* space; yellow = 5, compounds < 5 are in the *drug-like* space; red = 15, compounds < 15 are in the KDS. Total number of compounds = 179.

Like the hydrogen bond donors, ideally compounds should not have too many hydrogen bond acceptors, although the benchmarks for inclusion into the various chemical spaces for this descriptor are higher, with limits of three, ten and 15 hydrogen bond acceptors for *lead-like*, *drug-like* and KDS. While most deep-sea natural products (circa 75%) can be considered *drug-like* for this parameter, not many (15.1%) had <3 hydrogen bond donors and therefore *lead-like*. After molecular weight, the number of hydrogen bond acceptors was the most discerning parameter for the deep-sea natural products to be classified as *lead-like* (15.1%). Akin to the results seen for the number of hydrogen bond donors, Fungi natural products had the lowest number of hydrogen bond acceptors (mean = 7.7, Figure 9), while Animalia natural products had the most (mean = 11.0), followed closely by Bacteria-derived compounds (mean = 10.1). A small number of the compounds boast a very high number of hydrogen bond acceptors (six compounds have >40 hydrogen bond acceptors).

**Figure 10:**
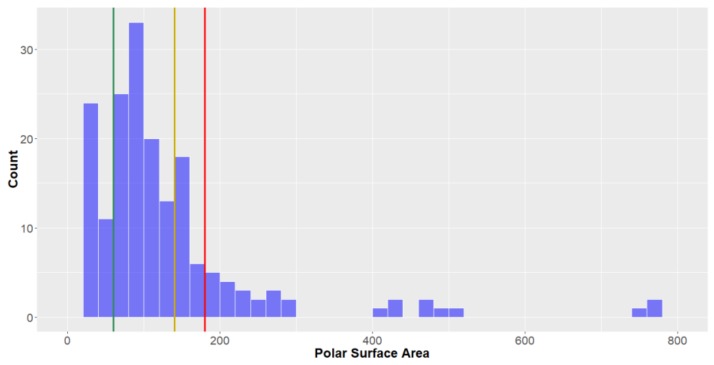
The statistical distribution of the polar surface area (PSA) of all analysed compounds (green = 60, compounds < 60 Å^2^ are in the *lead-like* space; yellow = 140, compounds < 140 Å^2^ are in the *drug-like* space; red = 180, compounds < 180 Å^2^ are in the KDS. Total number of compounds = 179.

*Polar surface area (PSA).* The polar surface area (PSA) of a compound is inherently linked to the number of hydrogen bond donors and acceptors—it is therefore unsurprising that the distribution of PSA for the molecules is right-skewed and natural products from Animalia have the highest PSA (mean = 140.2 Å^2^, Figure 10), followed by Bacteria (mean = 131.4 Å^2^) and Fungi (mean = 101.3 Å^2^). The overall mean PSA for all compounds was 131.1 ± 122.3 Å^2^. It has been reported that the highest PSA at which oral absorption can take place is 140 Å^2^, thereby benchmarking this parameter for *drug-like* space [39,40]; 70% of the compounds in this study were *drug-like*. Only 20% of deep-sea natural products were within the strict bounds of PSA for *lead-like* space (PSA ≤ 60 Å^2^), however in contrast 84% of compounds were in KDS for this molecular descriptor.

**Figure 11:**
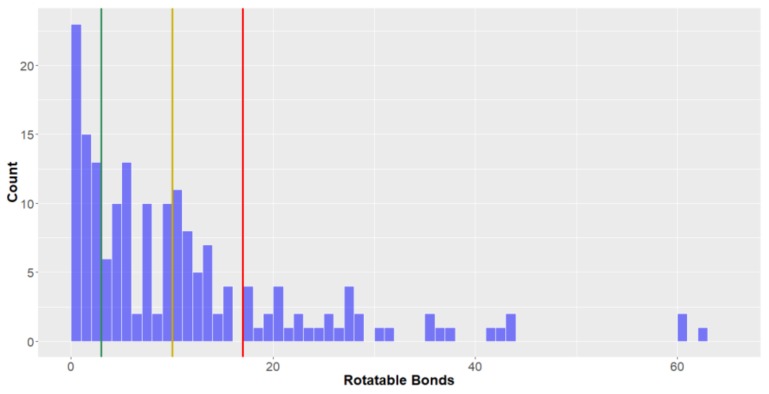
The statistical distribution of the rotatable bonds of all analysed compounds (green = 3, compounds < 3 are in the lead-like space; yellow = 10, compounds < 10 are in the drug-like space; red = 17, compounds < 17 are in the known drug space. Total number of compounds = 179.

*Rotatable bonds*. The number of rotatable bonds in deep-sea natural products can be seen to be highly variable (Figure 11), ranging from 0 to >60 (mean = 11.6, standard deviation = 11.9). It has been previously reported that compounds are considered to be in *privileged property space* if they have ≤10 rotatable bonds which is therefore the upper limit for a compound to be in *drug-like* for this parameter [40]. For *drug-like* space, the number of rotatable bonds was the most discerning factor where it was found that only 58.1% of deep-sea natural products were *drug-like* when considering this parameter. The proportion of compounds that could be classified in KDS was also the lowest for number of rotatable bonds compared to the other parameters studied (78.8% ≤ 17 rotatable bonds). On average, Fungi-derived deep-sea natural products had far fewer rotatable bonds (mean = 3.5) than compounds from both bacteria (mean = 8.4) and animals (mean = 15.4).

**Figure 12:**
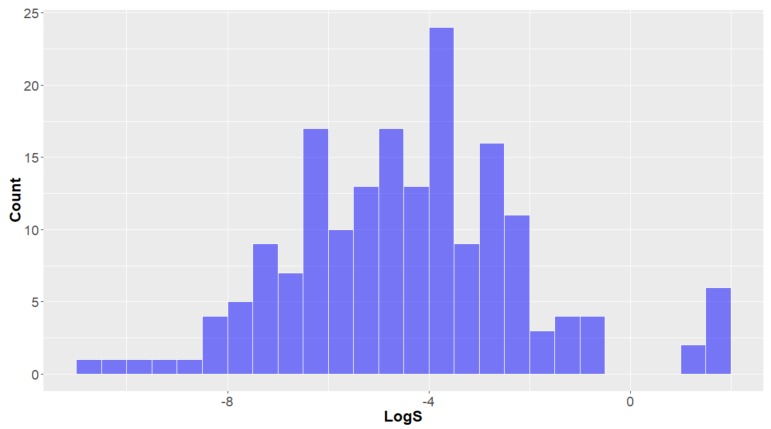
The statistical distribution of the LogS of all analysed compounds. Total number of compounds = 179.

*Other molecular descriptors*. The water solubility (LogS) of a compound is an important property that is a good indicator of the oral availability of a compound—research has demonstrated that most orally-available drugs have a LogS between 0 and −7 and mean between −4 and −3 [39,41]. This distribution looks to be emulated by the studied compounds (normal distribution, overall mean = −4.4 ± 2.4, Figure 12), indicating that most of these deep-sea natural products would be orally available, based on this parameter. Water solubility is inherently (inversely) linked to the lipophilicity (LogP) of a compound, therefore it is no surprise that natural products from animalia have, on average, lower hydrophilicity (mean LogS = −4.9), where those sourced from Bacteria (mean = −3.9) and Fungi (mean = −3.7) have a greater affinity for aqueous systems—this was the opposite trend to that seen for lipophilicity (LogP).

**Figure 13:**
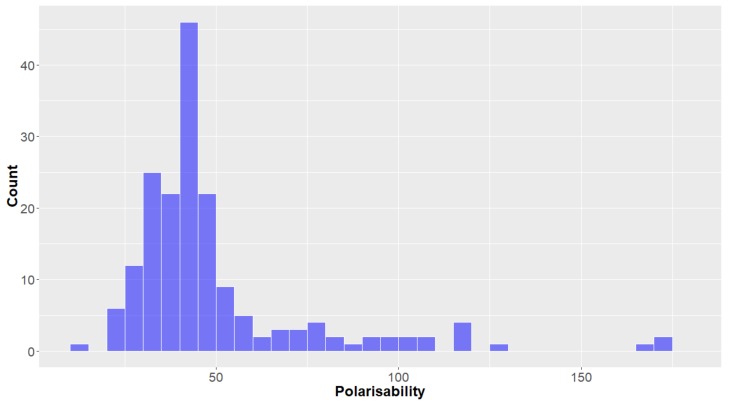
The statistical distribution of the polarisability of all analysed compounds. Total number of compounds = 179.

The polarisability of a compound has been closely associated with the cell permeability of a drug and it has been shown that a compound should have a polarisability ≤68 Å^3^ to be in KDS [42]. Not all of the deep-sea natural products fulfil this criterion, with 16% of compounds having a polarisability value greater than this benchmark. The polarisability of the compounds can be seen to have a right-skewed distribution (mean = 49.8 Å^3^, Figure 13). Of all the parameters studied, the differences between this molecular descriptor for compounds originated from organisms of the different Kingdoms seemed to be the least notable, although it can be observed that the polarisability of natural products from animals was the highest, followed by bacteria and then fungi (mean = 54.7, 46.0 and 38.7 for animals, bacteria and fungi, respectively).

**PCA (Principal Component Analysis).** Subsequent to analysing the molecular descriptors separately, it was also decided to analyse their interrelationships through both a correlation analysis as well as PCA (Principal Component Analysis). PCA is a form of analysing the data by transforming all the data into principal components of a set of new, uncorrelated, dimensions. Principal components are a combination of the variables (in this study, molecular descriptors) studied, designed to maximise the variation that is explained by the principal component. When one performs and analyses a PCA, they can comprehensively explore the oft-complex interactions and interrelationships between all the variables, simultaneously. Furthermore, PCA can investigate if groups of samples (in this investigation, compounds) can be distinguished based on the many variables and which of these variables are important for distinguishing differences between them.

**Figure 14:**
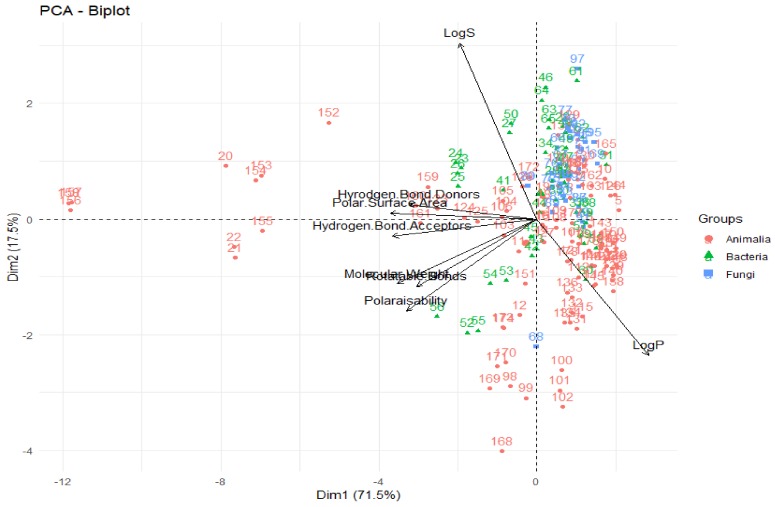
Biplot representing the PCA analysis on the studied compounds and molecular descriptors. The arrows represent molecular descriptors and the direction in which they hold influence. Each point represents a molecule in this study (red = animalia, green = bacteria, blue = fungi).

One of the most informative ways to view the results of a PCA is to plot the principal components against each other (i.e., principal component 1 vs. principal component 2) to give a biplot, as shown in Figure 14. There are a number of interpretations that can be made from a biplot; firstly, that the vectors/arrows on the plot signify the direction of influence of each molecular descriptor studied and the data points represent each deep-sea natural product in the investigation. In the biplot in Figure 14, the first two principal components are represented—the first principal component on the *x*-axis and the second on the *y*-axis. In PCA, the first principal component accounts for the greatest variability in the data (in this case, 71.5%), while the second principal component is the dimension in which the data are the second-most variable (explaining 17.5% of the variability in molecular descriptors for deep-sea natural products). As ~90% of the variability in the data in this study can be explained by the first two principal components, one can claim that PCA is a very effective technique for this analysis—it captures and represents a significant proportion of the differences and similarities of the compounds in terms of their molecular descriptors and can provide many useful conclusions and observations.

**Figure 15:**
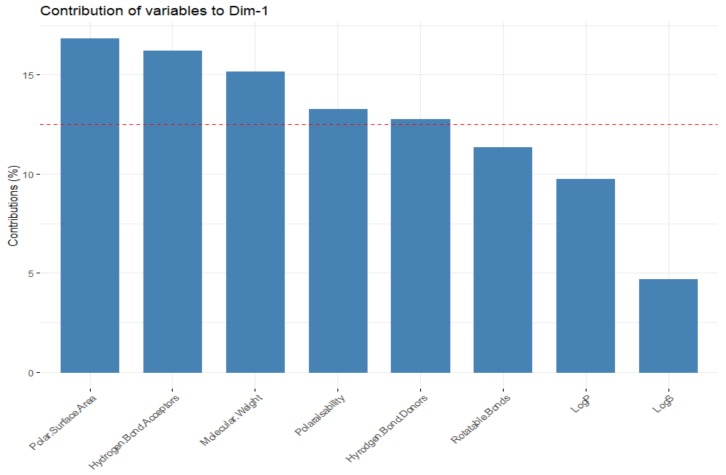
Representation of the contributors to the first principal component. The red reference line corresponds to the expected contribution value for each dimension if the contribution were uniform.

**Figure 16:**
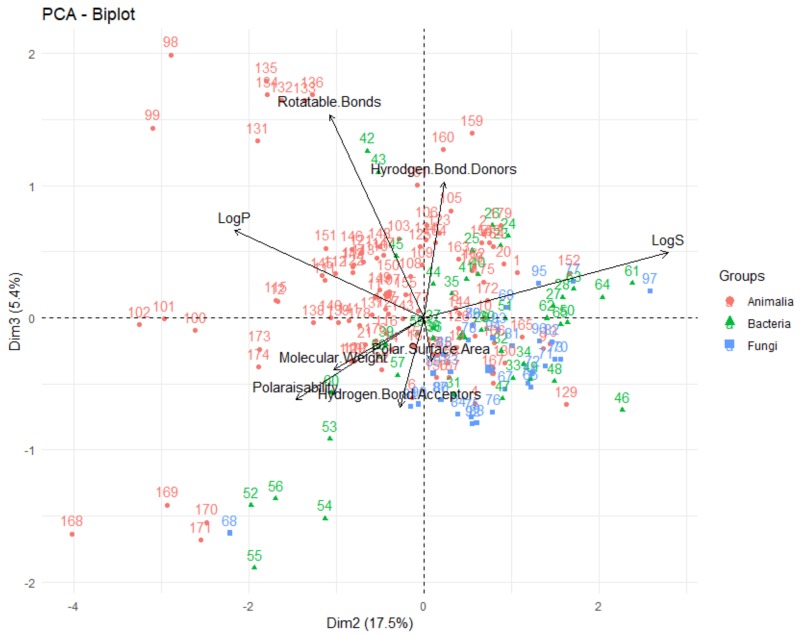
Biplot representing the PCA analysis on the studied compounds and molecular descriptors. The arrows represent molecular descriptors and the direction in which they hold influence. Each point represents a molecule in this study (red = animalia, green = bacteria, blue = fungi).

Firstly, looking at the interrelationships between variables (molecular descriptors), it can be seen that principal component one (corresponding to the dimension of the data that explains the greatest amount of variability, on the *x*-axis of Figure 14) is largely influenced by the polar surface area, number of hydrogen bond donors and acceptors, molecular weight and polarisability. This can be seen by the fact that these variables have a significant horizontal component to their direction (i.e., the direction of the arrows representing the variables are mainly horizontal) in Figure 14, with Figure 15 quantifying this contribution. Along with contributing largely to the first principal component, the polarisability of the compound contributes significantly to principal component 2 (i.e., is orientated mostly in the vertical direction), along with LogP and LogS. The number of rotatable bonds is the largest contributor to principal component 3 (vertical direction in biplot in Figure 16).

It can also be seen from this PCA, that in general, for these deep-sea natural products, the greater the LogP value (i.e., the greater the lipophilicity), the lower the number of hydrogen bond donors, acceptors and polar surface area for these compounds, as these vectors are in the opposite direction to the LogP, signifying an inverse relationship. This concurs with what one would expect—substrates with more hydrogen bond donors, acceptors and polar surface area are anticipated to be less lipophillic and have lower LogP values. Furthermore, the number of hydrogen bond donors, acceptors and polar surface area are highly correlated, as is signified through them acting in very similar directions in the biplot. This again coincides with what one would predict—a compound with the more hydrogen bond donors and acceptors would be envisaged to have a greater polar surface area. It has been shown that there is a high degree of correlation (r^2^ = 0.90) between polarisability and the molecular weight of a compound, which can account for why molecular weight is a crucial parameter for the definition of chemical spaces [43]. It can be seen that this correlation is true for these deep-sea natural products—the PCA shows very close alignment of the polarisability and molecular weight vectors, signifying a high correlation between these parameters. Furthermore, it is clear that LogS (hydrophilicity) is aligned in the opposite orientation to the LogP (lipophilicity) vector, as would be anticipated for these two inversely-related metrics.

**Figure 17:**
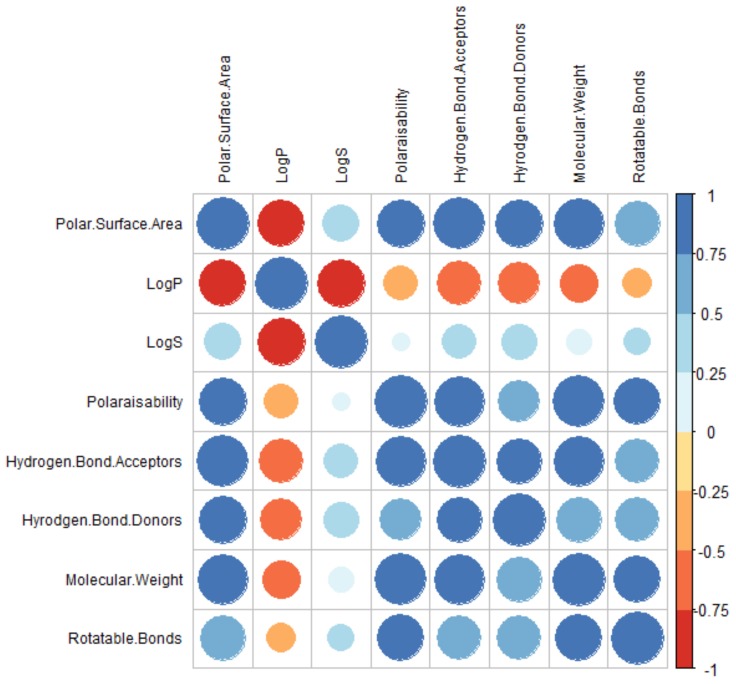
Correlation matrix to represent the relationships between the molecular descriptors.

The correlations between variables can also be represented using a correlation matrix, as seen in Figure 17. It can be seen that all variables are, overall, positively correlated with one another, except for the lipophilicity (LogP), which has a negative correlation with all other variables, i.e., as these other variables increase, the LogP decreases, and vice versa. All the other variables have strong intercorrelations (>0.5), except for LogS, which have weaker, but positive, relationships.

The relative positions of points situated on a PCA plot are a culmination of their values for the various parameters and any observed groupings indicate similarities in the physicochemical properties between compounds in that group. It can be seen that PCA with the eight molecular descriptors that have been analysed in this study has been able to show that there are general physicochemical differences in deep-sea natural products isolated from organisms from different kingdoms—Animalia (red), Bacteria (green) and Fungi (blue). Therefore, this analysis can give general indications about the groupings. Studying Figure 14 and Figure 16, it can be seen that natural products from members of the Animalia Kingdom are more varied than compounds from Bacteria and Fungi. Bacteria and Fungi-derived natural products generally have lower molecular weights, number of hydrogen bond donors and acceptors, polarisability, hydrophilicity (LogS) and polar surface area than those from animals (Figure 14). Conversely, compounds sourced from animals have higher molecular weights, number of hydrogen bond donors and acceptors, polarisability, polar surface area and lower hydrophilicity (and higher lipophilicity). Principal component three (Figure 16) demonstrates that metabolites derived from fungi have a lower number of rotatable bonds, then bacteria, with those from animals generally having more rotatable bonds.

**Deep-Sea Natural Products in Chemical Space.** The proportion of all deep-sea natural products in this study that lies within the benchmarks for *lead-like*, *drug-like* and KDS as specified in Table 1, is shown, for each molecular descriptor and when all molecular descriptors are taken into account (Table 3). As can be seen, a very small percentage (0.5%, 1 of the 179 compounds) is considered to be *lead-like* when all of the molecular descriptors are taken into account. Looking at the criteria, most (77.1%) of compounds had ≤3 hydrogen bond donors and approximately half had a LogP value sufficiently low enough (i.e., was appropriately hydrophilic), most compounds were too large, had too many hydrogen bond acceptors and rotatable bonds and had a polar surface area greater than the low limiting threshold for *lead-like* space. For compounds to be classified as *lead-like,* structures should possess low molecular complexity and the limiting values for the physicochemical descriptors for *lead-like* space reflect this—*lead-like* compounds should have low molecular weights, minimal numbers of hydrogen bond acceptors, hydrogen bond donors and rotatable bonds. The strictest criterion for the chemical spaces is those that define *lead-like* space [30]. Lead structures need to be simple, thereby offering a scaffold to which further complexity can then be added, to provide *drug-like* compounds.

Approximately 40% of the deep-sea natural products are *drug-like* in that they already have structures with greater complexity than one would expect from a lead compound and, by definition, possess properties and characteristics that indicate they would be appropriate for use as therapeutics. An even higher proportion (~2/3) of the compounds studied are in KDS, thus are in the chemical space that is defined by known drugs.

Furthermore, SEA (Similarity Ensemble Approach) analysis was conducted on each of the *drug-like* compounds in this study to identify possible enzyme targets [44]. SEA compares the compound(s) of interest against an extensive set of ligands that bind to known targets to assess if the compound(s) in question are similar to the ligand and could potentially interact with the associated target. For some of the compounds in this study, there were no significant hits, however for others, several putative enzyme targets were identified and offer a starting point for biological investigation of these natural products and associated analogues (see Table S2).

**Animalia Kingdom.** As noted in the PCA analysis, the 104 compounds derived from Animalia were the most varied in their attributes, but generally had higher LogP values (i.e., higher lipophilicities), polarisability, polar surface area and number of rotatable bonds, hydrogen bond donors and acceptors (Table 2, Figure 14 and Figure 16). The statistical distributions of all the studied molecular descriptors of these compounds can be found in the Supporting Information (Figures S1–S8).

**Figure 18:**
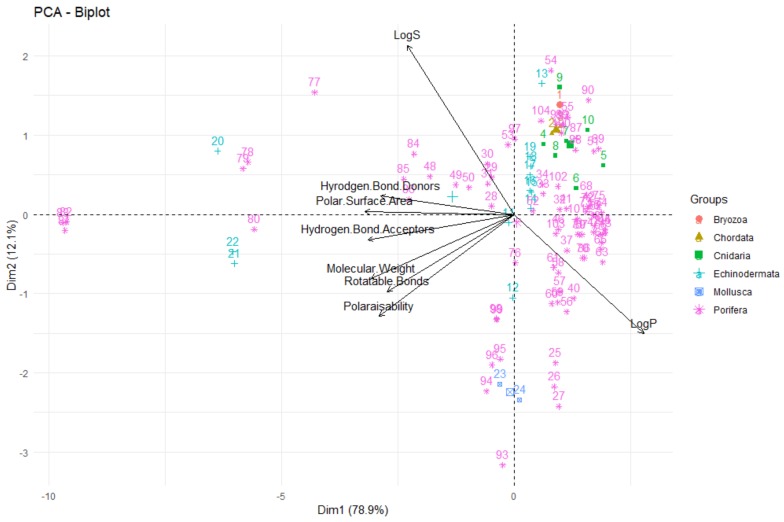
Biplot representing the PCA analysis on the compounds isolated from organisms in the Animalia Kingdom and their molecular descriptors (PC1 vs. PC2). The arrows represent molecular descriptors and the direction in which they hold influence. Each point represents a molecule.

**Figure 19:**
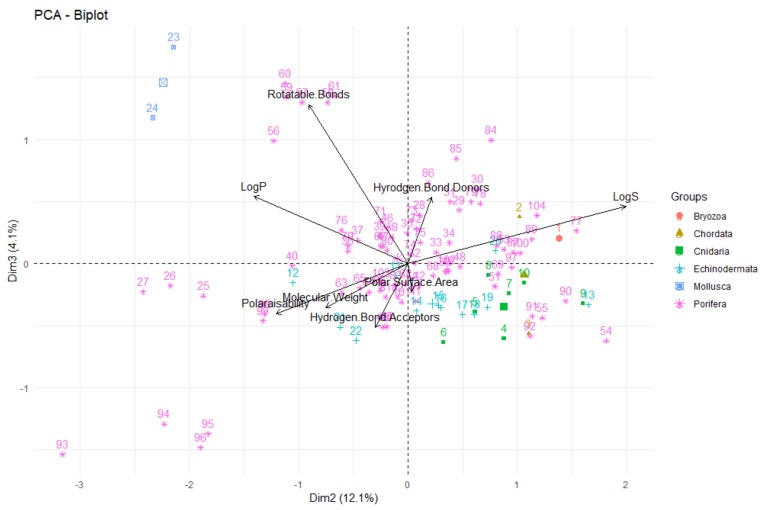
Biplot representing the PCA analysis on the compounds isolated from organisms in the Animalia Kingdom and their molecular descriptors (PC2 vs. PC3). The arrows represent molecular descriptors and the direction in which they hold influence. Each point represents a molecule.

To investigate any apparent differences in the compounds isolated from different Phylum in the Animalia Kingdom, a PCA was conducted (see Figure 18 and Figure 19 for the associated biplots). As was previously noted, the most common Phylum from which deep-sea natural products are derived from are Porifera (sea sponges) and it is apparent that these compounds are highly variable in their structural composition. This high variability could be due to the fact that microorganisms are known to coexist with sea sponges and the natural products isolated from sea sponges could in fact be produced by these microorganisms. The next populous source of compounds was organisms of the Echinodermata Phylum—in general the compounds tend to have lower lipophilicity (LogP). In contrast, the two compounds obtained from molluscs had a very high lipophilic character and large numbers of rotatable bonds. The compounds isolated from the other Phyla, namely Bryozoa, Chordata and Cnidaria, were all very structurally similar, typically with low molecular weights, polarisability, polar surface area and numbers of hydrogen bond donors and acceptors and rotatable bonds than that seen from compounds from Porifera, Echinodermata and Mollusca organisms. It should be noted, however, that the low numbers of compounds isolated from organisms in some of the Phyla (Bryozoa, Chordata, Cnidaria and Mollusca) limits the strength of the conclusions that can be made about these underrepresented groups.

**Figure 20:**
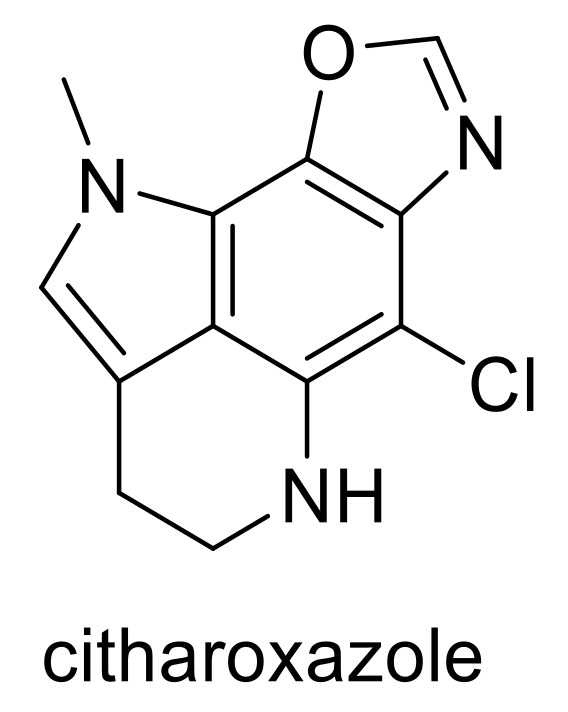
Structure of citharoxazole, a *lead-like* compound isolated from the deep-sea Mediterranean sponge, *Latrunculia (Biannulata) citharistae* [45].

The only compound in the study that fulfilled all of the criteria to be considered *lead-like* was from an organism of the Animalia Kingdom, citharoxazole, which was isolated from the deep-sea Mediterranean sponge, *Latrunculia (Biannulata) citharistae* from a depth of 103 m off La Ciotat, Banc de Banquiere in France by Genta-Jouve et al. (Figure 20) [45]. No biological assessment was performed at the time and subsequent to its isolation, no further research has been reported on this interesting compound, presenting it as an attractive option for further investigation. Unfortunately, SEA analysis did not offer any potential biological targets, possibly due to unique features in its structure, rendering it difficult to classify as analogous and similar to existing ligands.

**Figure 21:**
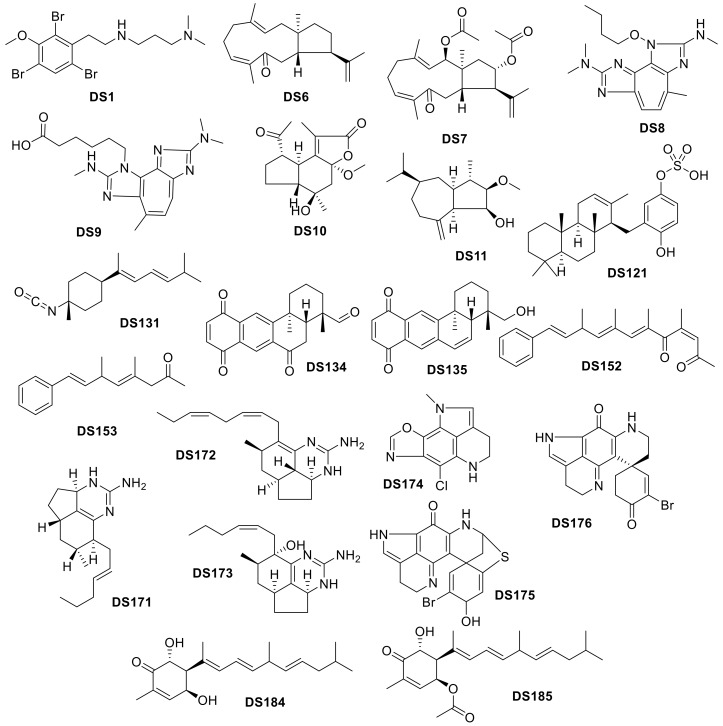
*Drug-like* compounds isolated from deep-sea Animalia organisms.

The majority of deep-sea natural products isolated from Animalia were not *lead-like* for most of the criterion, with the exception of the number of hydrogen bond donors (75% had ≤3 hydrogen bond donors, Table 4). In contrast, greater than half of the compounds met the benchmarks for *drug-likeness* for the descriptors analysed (the exception being that only ~40% of the compounds had ≤10 rotatable bonds), and when the criterion were taken collectively ~20% of the natural products isolated from Animalia species are considered *drug-like* (Figure 21). In total, 54.8% of Animalia-derived deep-sea natural products were in KDS.

**Bacteria Kingdom.** While deep-sea natural products isolated from bacteria were also found to be variable in their molecular composition, they were not as variable as those found in animals, particularly not reaching the high values for certain descriptors (Figure 16). In contrast to Animalia-derived natural products, those from Bacteria were found to be typically smaller with notably lower molecular weights, lipophilicities and rotatable bonds, and similar (but lower) number of hydrogen bond donors, acceptors, polar surface area and polarisability (see Figures S9–S16 for the statistical distributions of all the studied molecular descriptors of these compounds).

**Figure 22:**
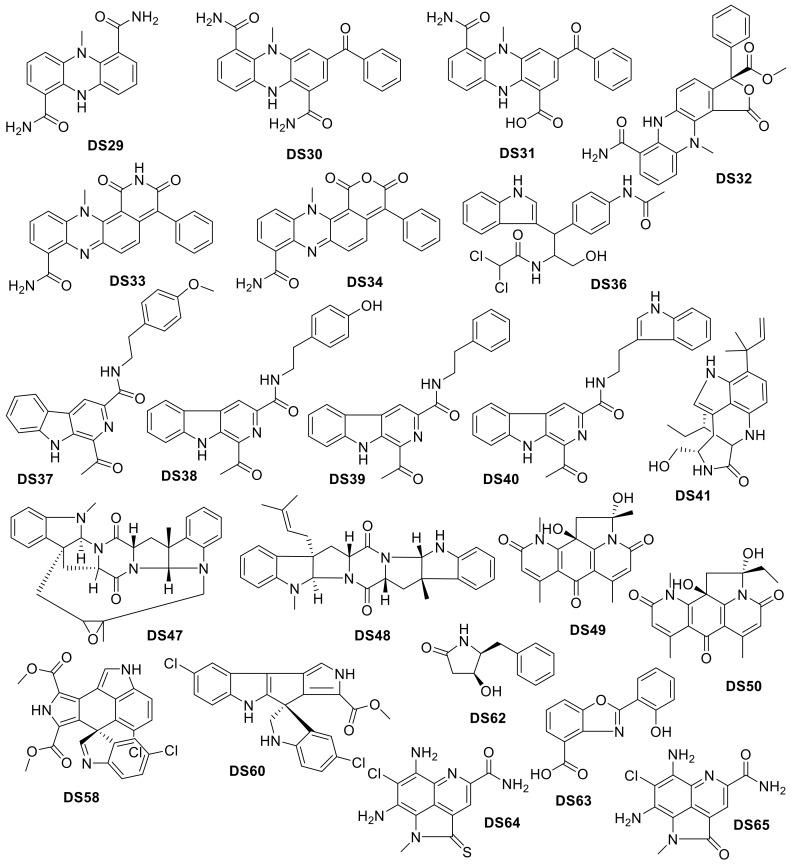
*Drug-like* compounds isolated from deep-sea Bacteria.

These general differences are apparent when studying the proportion of these compounds in each of the studied chemical spaces; while no bacteria-derived deep-sea natural products were *lead-like* when taking all of the studied molecular descriptors into account, greater than half (53.5%) are *drug-like* (Figure 22) and nearly ¾ (72.1%) are in KDS (Table 5). The most discriminating features for these compounds that led them to not be considered *lead-like* was their polar surface areas—only one of the 43 compounds had a polar surface area ≤60 Å^2^, and that only 3 of the 43 compounds had three or less hydrogen bond acceptors.

**Figure 23:**
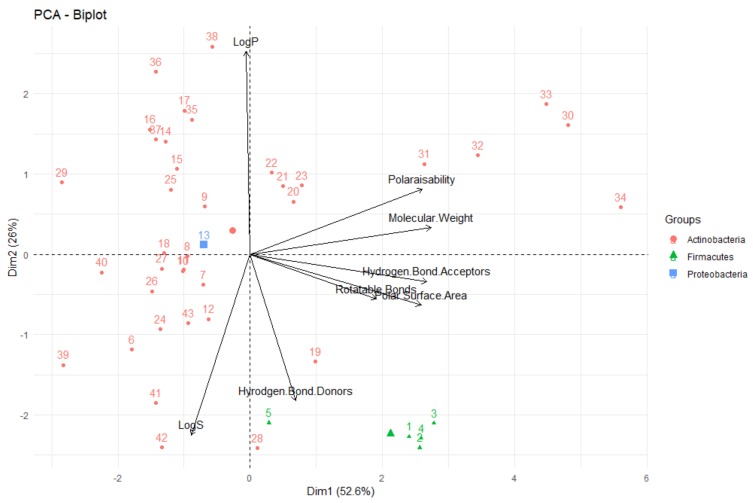
Biplot representing the PCA analysis on the compounds isolated from organisms in the Bacteria Kingdom and their molecular descriptors (PC1 vs. PC2). The arrows represent molecular descriptors and the direction in which they hold influence. Each point represents a molecule.

**Figure 24:**
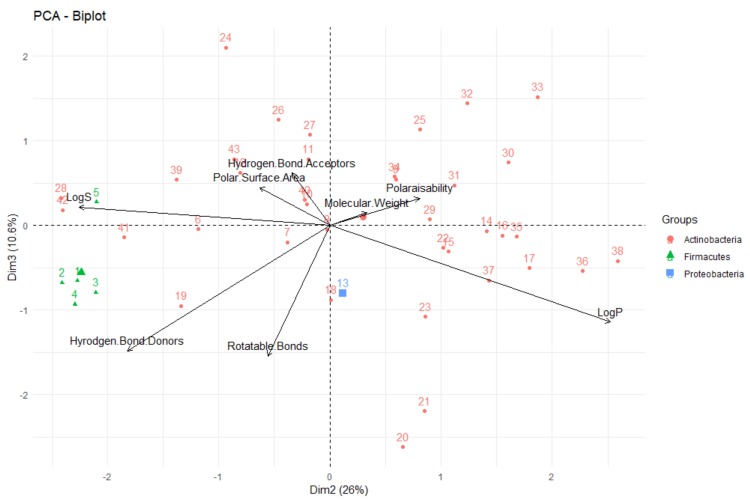
Biplot representing the PCA analysis on the compounds isolated from organisms in the Bacteria Kingdom and their molecular descriptors (PC2 vs. PC3). The arrows represent molecular descriptors and the direction in which they hold influence. Each point represents a molecule.

Analysing the Bacteria-derived compounds by Phylum through PCA (Figure 23 and Figure 24), it is apparent that most of the compounds were from Actinobacteria organisms and that these compounds were more structurally-diverse than the small group from the Firmacutes Phylum (from the one marine bacterium). This group of five natural products had low lipophilicity (and high water solubility) and a large number of hydrogen bond donors, compared to the other compounds isolated from Bacteria, but due to all of these compounds all being isolated from the same organism, it could be that this is not representative of all compounds from this Phylum.

**Fungi Kingdom.** The natural products isolated from Fungi were by far the least variable in terms of their molecular descriptors, compared to those isolated from animals and bacteria (Figure 14 and Figure 16). Fungi-derived compounds were consistently lower in molecular weight, number of hydrogen bond donors, acceptors and number of rotatable bonds, polar surface area and polarisability, with mean values notably less than those calculated for their counterparts from Animalia and Bacteria (Table 2, see Figures S17–S24 for the statistical distributions of all the studied molecular descriptors of these compounds). However, Fungi-derived isolates boasted the highest calculated water solubility (mean LogS = −3.7), much greater than the more lipophilic compounds from the Animalia organisms (mean LogS = −4.9) although they were followed closely by those from Bacteria (mean LogS = −3.9). There were no discernible differences between compounds isolated from the three classes of Fungi represented in this study, but this may have been due to a smaller sample size (see Figure S25).

**Figure 25:**
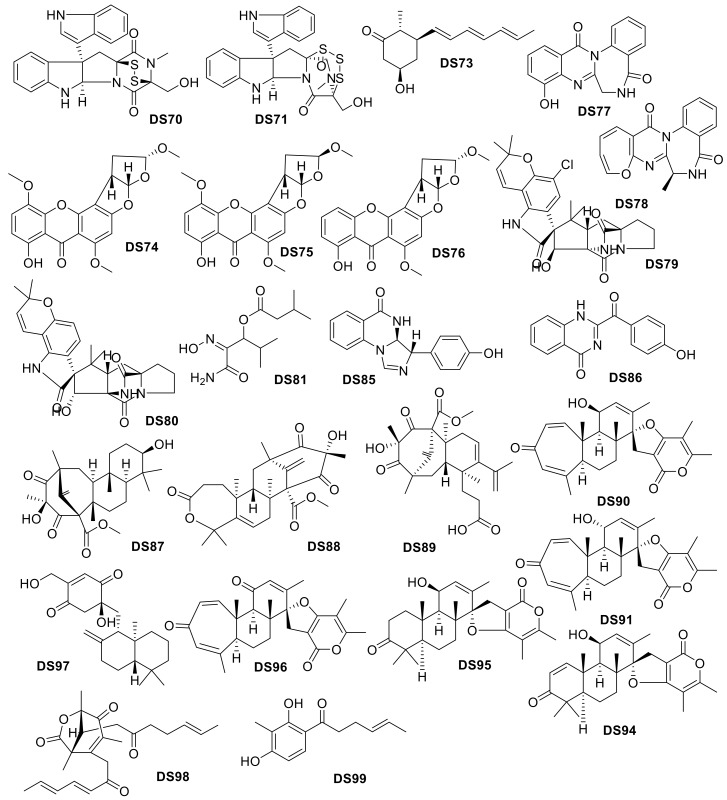
*Drug-like* compounds isolated from deep-sea Fungi.

The lower values for the guidelines that dictate chemical space translates into much higher proportions of Fungi-derived compounds being *drug-like* and in KDS (Table 6), with ~80% of compounds classified as *drug-like* (Figure 25) and 90.6% in KDS. As for those isolated from Bacteria, it was the very low-value benchmarks for polar surface area and hydrogen bond donors that limited their inclusion into *lead-like* space. Nevertheless, with a vast majority of compounds in *drug-like* and KDS, Fungi are clearly a rich source of compounds with great potential as therapeutics.

## 3. Conclusions

In this study, 179 deep-sea natural products were investigated, analysing their physicochemical properties, general trends and the variability in these parameters between and within compounds isolated from different Kingdoms; Animalia, Bacteria and Fungi. The study and analysis of these molecular descriptors and characteristics also enabled the defining of these compounds in various chemical spaces, particularly as an indication of their *drug-likeness.* Only one natural product, citharoxazole, isolated from the deep-sea Mediterranean sponge, *Latrunculia (Biannulata) citharistae* was found to be *lead-like* in that it fulfilled requirements to be in *lead-like* space for all six molecular descriptors (*lead-like* structures are those with low molecular complexity with the view of being a useful and promising scaffold, to which further moieties can be added to create a *drug-like* compound). Citharoxazole has had no assessment of its biological activity, neither upon isolation nor subsequently, and presents an attractive option for further investigation. It was found, however, that approximately 40% of all deep-sea natural products are in *drug-like* chemical space and 2/3 were within KDS. These results strongly advocate for the *drug-likeness* of a significant proportion of deep-sea natural products, many of which have already been shown to have notable biological activities that should be further investigated as potential therapeutics.

Multi-variate statistical analysis of the molecular descriptors investigated in this study, using the unsupervised technique of PCA, was particularly useful in highlighting the various interrelationships between these physicochemical properties. It was seen that the number of hydrogen bond donors, acceptors and the polar surface area were highly correlated, and these molecular descriptors had an inverse relationship with lipophilicity (LogP). Hydrophilicity (LogS) was also clearly negatively correlated to LogP, as one would expect. Furthermore, a strongly positively correlated relationship was observed between polarisability and the molecular weight of a compound.

PCA was also very useful in observing the general differences between deep-sea natural products by their source (Kingdom: Animalia, Bacteria and Fungi). It was observed that natural products from members of the Animalia Kingdom are more varied than compounds from Bacteria and Fungi. Bacteria and fungi-derived natural products, generally, have lower molecular weights, number of hydrogen bond donors and acceptors, polarisability, hydrophilicity (LogS) and polar surface area than those from animals. Furthermore, it was demonstrated that natural products isolated from Fungi have a lower number of rotatable bonds, then Bacteria and those from animals generally have more rotatable bonds.

The differences seen between the different groups in the PCA were also reflected in the differing proportions of deep-sea natural products isolated from various organism Kingdoms, that were included in the chemical spaces, particularly in the *drug-like* chemical space. Only ~20% of natural products from animals were *drug-like*, while ~50% from Bacteria and >80% from Fungi were classified as such. This pattern of Fungi-derived compounds overall occupying a more favourable position in chemical space was also shown when looking at the proportion in KDS; ~90% of Fungi-derived products, vs. 81% and 55% of those isolated from Bacteria and Animalia species, respectively. This particularly highlights deep-sea Fungi species as a rich and promising source of biologically active natural products for the purposes of drug development and therapeutic application. However, the results presented here reveal the overall *drug-likeness* that deep-sea natural products possess. Coupled with their potent biological activities, their physicochemical properties indicate that there is significant value in their study as promising future drug leads. This study also showcases a methodology by which natural products can be assessed for their viability as potential therapeutics and presents a strategy for determining future directions for research in this area of drug discovery.

## 4. Materials and Methods

The 3D structures of the compounds were drawn using the ChemBioOffice 2010 [46] software package. The structures were then optimised using the MM2 [47] force field in Chem3D 15.1 [46]. The molecular descriptors of the optimised structures were calculated using QikProp 4.42 [48] which has been shown to be an accurate and reliable tool for the calculation of the molecular descriptors [43].

After calculation of the molecular descriptors for the compounds, the mean, median and standard deviation of each descriptor was calculated for all the compounds and also for the compounds classified by the Kingdom of the organism from which they were originated (Table 2). Graphs of the distributions of these molecular descriptors were generated with R (version 3.2.2) [49] and R Studio (version 0.99.486) [50] using the ggplot2 package [51]. Compounds were classified as *lead-like*, *drug-like* and in KDS for each of the molecular descriptors by comparing the values for the descriptors against the benchmarks stated in Table 1 and including them in the chemical space if the calculated value was less than or equal to the stipulated value.

PCA (Principal Component Analysis) was carried out using all compounds and parameters included in this study using R (version 3.2.2) [49] and R Studio (version 0.99.486) [50]. PCA analysis was performed using the prcomp function as part of the stats package, by singular value decomposition of the centred and scaled data matrix [49]. Results of this analysis were visualised using the factoextra package (version 1.0.5) [52].

## Figures and Tables

**Table 1:** Definition of *lead-like*, *drug-like* and known drug space (KDS) in terms of molecular descriptors.

Molecular Descriptor	*Lead-Like* Space	*Drug-Like* Space	Known Drug Space
Molecular weight (g mol^−1^)	300	500	800
Lipophilicity (Log P)	3	5	6.5
Hydrogen bond donors	3	5	7
Hydrogen bond acceptors	3	10	15
Polar surface area (Å^2^)	60	140	180
Rotatable bonds	3	10	17

**Table 2:** Mean, standard deviation (std dev) and median values of the compound types for the eight molecular descriptors analysed in this study.

Molecular Descriptor		Animalia (*n* = 104)	Bacteria (*n* = 43)	Fungi (*n* = 32)	Overall (*n* = 179)
**Molecular weight (g mol^−1^)**	**Mean**	611.8	491.2	403.4	545.6
	**Std Dev**	390.4	196.4	132.7	327.5
	**Median**	441.1	432.4	436.5	436.5
**Lipophilicity (LogP)**	**Mean**	3.7	2.2	2.3	3.1
	**Std Dev**	4.0	1.8	1.1	3.3
	**Median**	4.1	2.4	2.1	3.2
**Hydrogen bond donors**	**Mean**	3.2	2.7	1.3	2.8
	**Std Dev**	3.5	1.6	0.8	2.9
	**Median**	2.0	2.0	1.0	2.0
**Hydrogen bond acceptors**	**Mean**	11.0	10.1	7.7	10.2
	**Std Dev**	12.9	6.4	2.3	10.4
	**Median**	7.0	8.5	7.3	7.5
**Polar surface area (Å^2^)**	**Mean**	140.2	131.4	101.3	131.1
	**Std Dev**	154.1	60.0	31.4	122.3
	**Median**	88.0	115.3	93.2	96.3
**Rotatable bonds**	**Mean**	15.4	8.4	3.5	11.6
	**Std Dev**	13.3	7.9	3.1	11.9
	**Median**	11.5	5.0	3.0	8.0
**Water solubility (LogS)**	**Mean**	−4.9	−3.9	−3.7	−4.4
	**Std Dev**	2.6	2.1	1.5	2.4
	**Median**	−5.2	−3.8	−3.7	−4.4
**Polarisability**	**Mean**	54.7	46.0	38.7	49.8
	**Std Dev**	30.9	17.7	12.4	26.3
	**Median**	43.3	41.8	41.7	42.9

**Table 3:** All compounds in this study and their inclusion within the defined chemical spaces.

Overall	*Lead-like* Space	*Drug-like* Space	Known Drug Space
Molecular weight (g mol^−1^)	14.5%	66.5%	84.9%
Lipophilicity (LogP)	48.0%	75.4%	88.8%
Hydrogen bond donors	77.1%	89.4%	91.1%
Hydrogen bond acceptors	15.1%	74.3%	84.4%
Polar surface area (Å^2^)	19.6%	70.4%	83.8%
Rotatable bonds	28.5%	58.1%	78.8%
All criteria	0.5%	39.7%	64.8%

**Table 4:** Compounds from Animalia and their inclusion within the defined chemical spaces.

Overall	*Lead-Like* Space	*Drug-Like* Space	Known Drug Space
Molecular weight (g mol^−1^)	12.5%	58.7%	78.8%
Lipophilicity (Log P)	31.7%	61.5%	80.8%
Hydrogen bond donors	75.0%	84.6%	84.6%
Hydrogen bond acceptors	22.1%	75.0%	81.7%
Polar surface area (Å^2^)	31.7%	65.4%	82.7%
Rotatable bonds	12.5%	42.3%	71.2%
All criteria	1.0%	21.2%	54.8%

**Table 5:** Compounds from Bacteria and their inclusion within the defined chemical spaces.

Overall	*Lead-Like* Space	*Drug-Like* Space	Known Drug Space
Molecular weight (g mol^−1^)	11.6%	67.4%	90.7%
Lipophilicity (Log P)	67.4%	93.0%	100.0%
Hydrogen bond donors	65.1%	93.0%	100.0%
Hydrogen bond acceptors	7.0%	67.4%	79.1%
Polar surface area (Å^2^)	2.3%	70.0%	79.1%
Rotatable bonds	41.9%	67.4%	81.4%
All criteria	0.0%	53.5%	72.1%

**Table 6:** Compounds from fungi and their inclusion within the defined chemical spaces.

Overall	*Lead-Like* Space	*Drug-Like* Space	Known Drug Space
Molecular weight (g mol^−1^)	25.0%	90.6%	96.9%
Lipophilicity (Log P)	78.1%	78.1%	100.0%
Hydrogen bond donors	100.0%	100.0%	100.0%
Hydrogen bond acceptors	3.1%	84.4%	100.0%
Polar surface area (Å^2^)	3.1%	87.5%	93.8%
Rotatable bonds	62.5%	100.0%	100.0%
All criteria	0.0%	81.3%	90.6%

## Supplementary Materials

The following are available online at https://www.mdpi.com/1420-3049/24/21/3942/s1, Figures S1–S8: Statistical distributions of each of the studied molecular descriptors for compounds isolated from organisms in the Animalia Kingdom, Figures S9–S16: Statistical distributions of each of the studied molecular descriptors for compounds isolated from organisms in the Bacteria Kingdom, Figures S17–S24: Statistical distributions of each of the studied molecular descriptors for compounds isolated from organisms in the Fungi Kingdom, Figures S25–S29: Additional biplot and scree plots for the PCA analysis, Table S1: Recently-isolated Deep Sea compounds analysed in this study, their identifiers, species isolated from and references, Table S2: Results of the SEA (Similarity Ensemble Approach) analysis conducted on each of the *drug-like* compounds in this study to identify possible enzyme targets.
