# Supplementary material for: A Chemometric Analysis of Deep-Sea Natural Products
Source: Molecules. 2019 Oct 31;24(21):3942. doi: 10.3390/molecules24213942 (PMC6865307; doi:10.3390/molecules24213942)
Supplement: Supplementary file 1 [file molecules-24-03942-s001.pdf]

Supporting Information for:

A Chemometric Analysis of Deep-Sea Natural Products

Lisa I. Pilkington[*]

School of Chemical Sciences, The University of Auckland, Auckland, New Zealand.

*To whom correspondence should be addressed: School of Chemical Sciences, The University of Auckland, Private Bag 92019, Auckland 1142, New Zealand.
E-mail: lisa.pilkington@auckland.ac.nz, Tel. 64-9-373-7599 ext. 86776

Contents:

Statistical distributions of the molecular descriptors for Animalia-derived compounds	S2-S5
Statistical distributions of the molecular descriptors for Bacteria-derived compounds	S6-S9
Statistical distributions of the molecular descriptors for Fungi-derived compounds	S10-S13
Additional PCA plots	S14-S16
Details of compounds analysed in this study	S17-S24
Results of SEA analysis of drug-like compounds	S26-S32
References	S33-S37

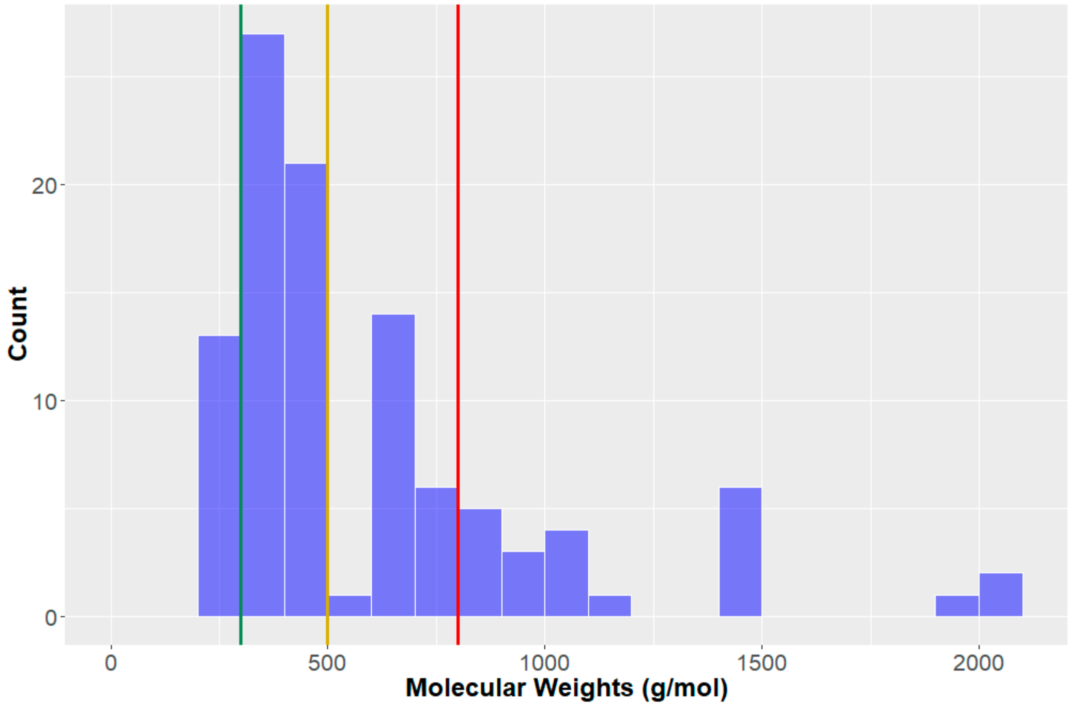

Figure S1. The statistical distribution of the molecular weight of analysed compounds isolated from organisms in the Animalia Kingdom (green = 300 g mol^{-1}, compounds < 300 g $mol^{-1}$are in the *lead-like* space; yellow = 500 g mol^{-1}, compounds < 500 g mol^{-1} are in the *drug-like* space; red= 800 g mol^{-1}, compounds < 800 g $mol^{-1}$are in the KDS. Total number of compounds = 104.

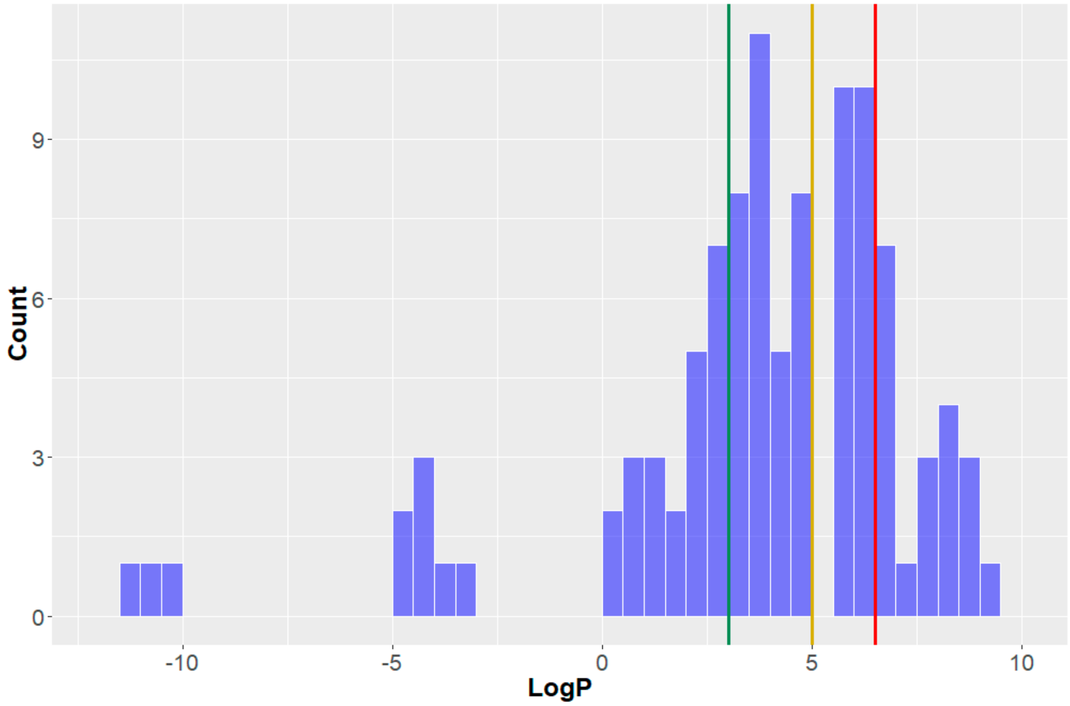

Figure S2. The statistical distribution of the octanol – water partition coefficient (LogP) of analysed compounds isolated from organisms in the Animalia Kingdom (green = 3, compounds < 3 are in the *lead-like* space; yellow = 5, compounds < 5 are in the *drug-like* space; red= 6.5, compounds < 6.5 are in the KDS. Total number of compounds = 104.

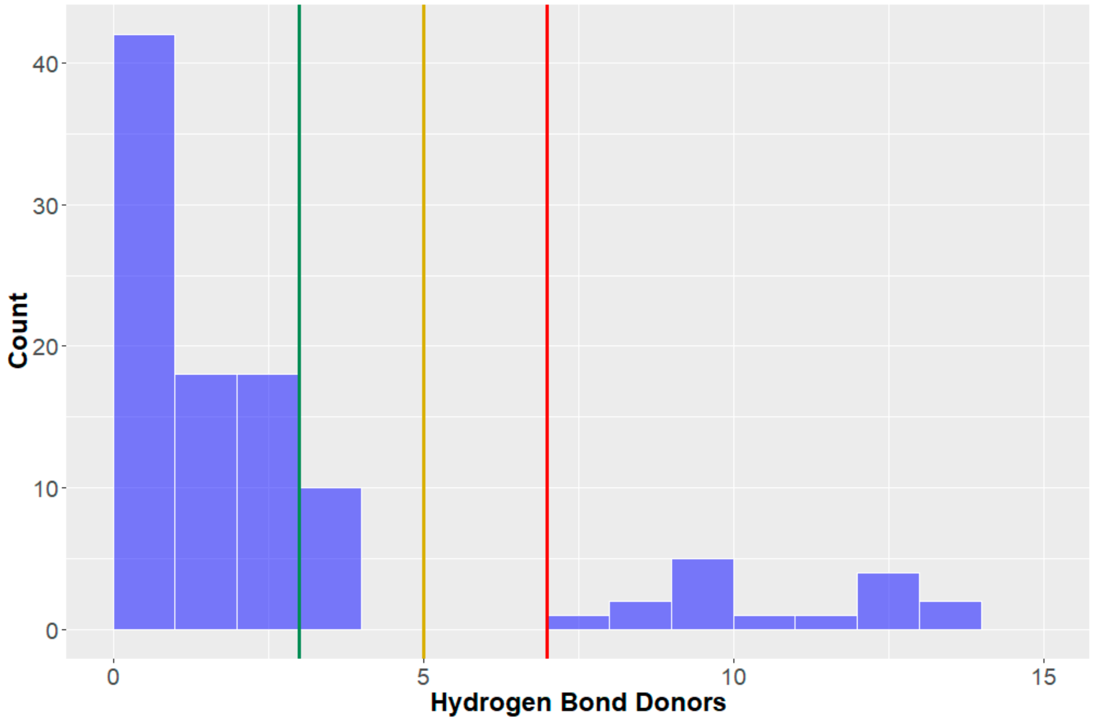

Figure S3. The statistical distribution of the hydrogen bond donors of analysed compounds isolated from organisms in the Animalia Kingdom (green = 3, compounds < 3 are in the *lead-like* space; yellow = 5, compounds < 5 are in the *drug-like* space; red= 7, compounds < 7 are in the KDS. Total number of compounds = 104.

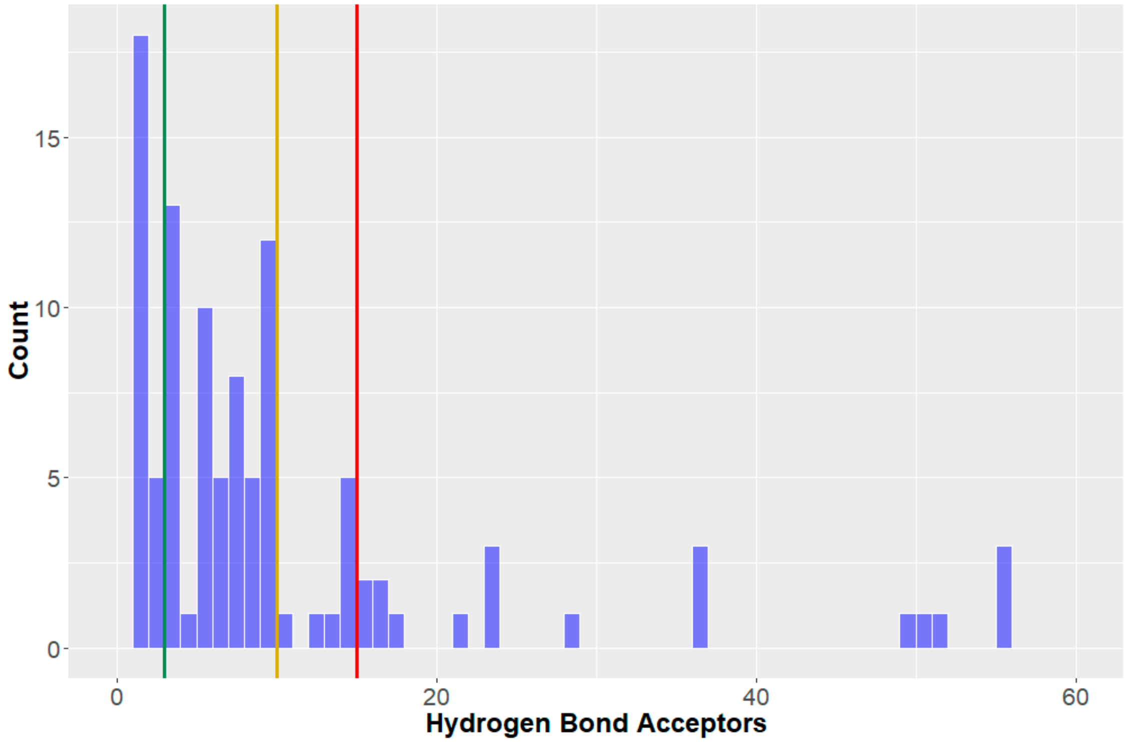

Figure S4. The statistical distribution of the hydrogen bond acceptors of analysed compounds isolated from organisms in the Animalia Kingdom (green = 3, compounds < 3 are in the *lead-like* space; yellow = 5, compounds < 5 are in the *drug-like* space; red = 15, compounds < 15 are in the KDS. Total number of compounds = 104.

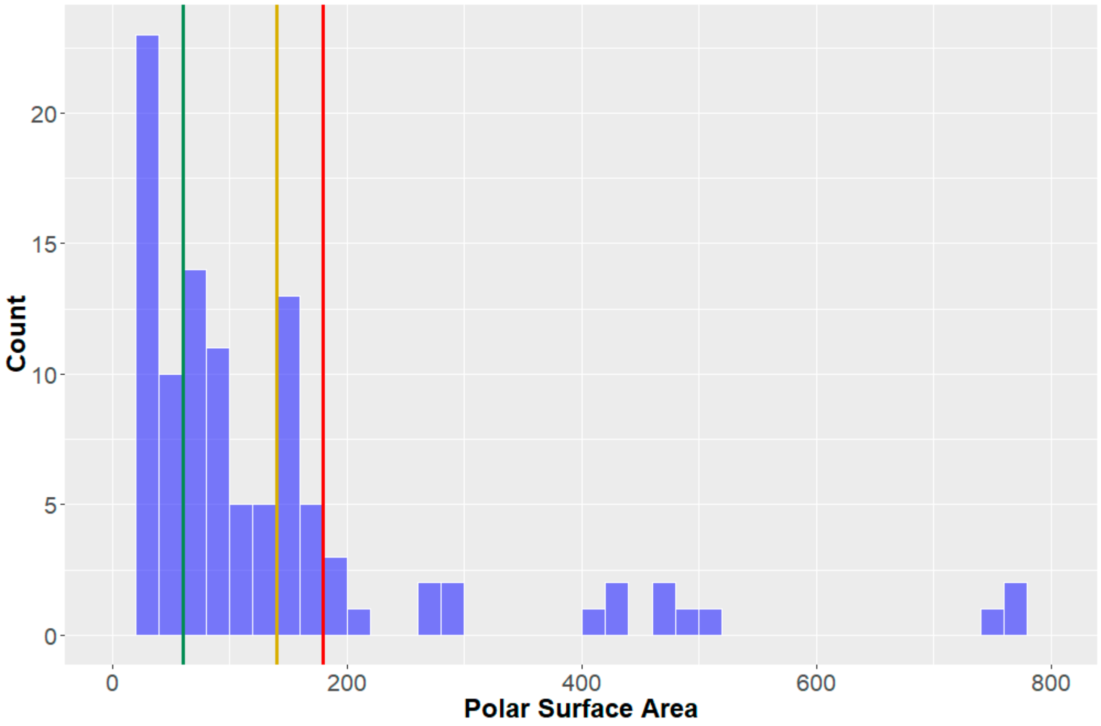

Figure S5. The statistical distribution of the polar surface area (PSA) of analysed compounds isolated from organisms in the Animalia Kingdom (green = 60, compounds < 60 Å^2 are in the *lead-like* space; yellow = 140, compounds < 140 Å^2 are in the *drug-like* space; red= 180, compounds < 180 Å^2 are in the KDS. Total number of compounds = 104.

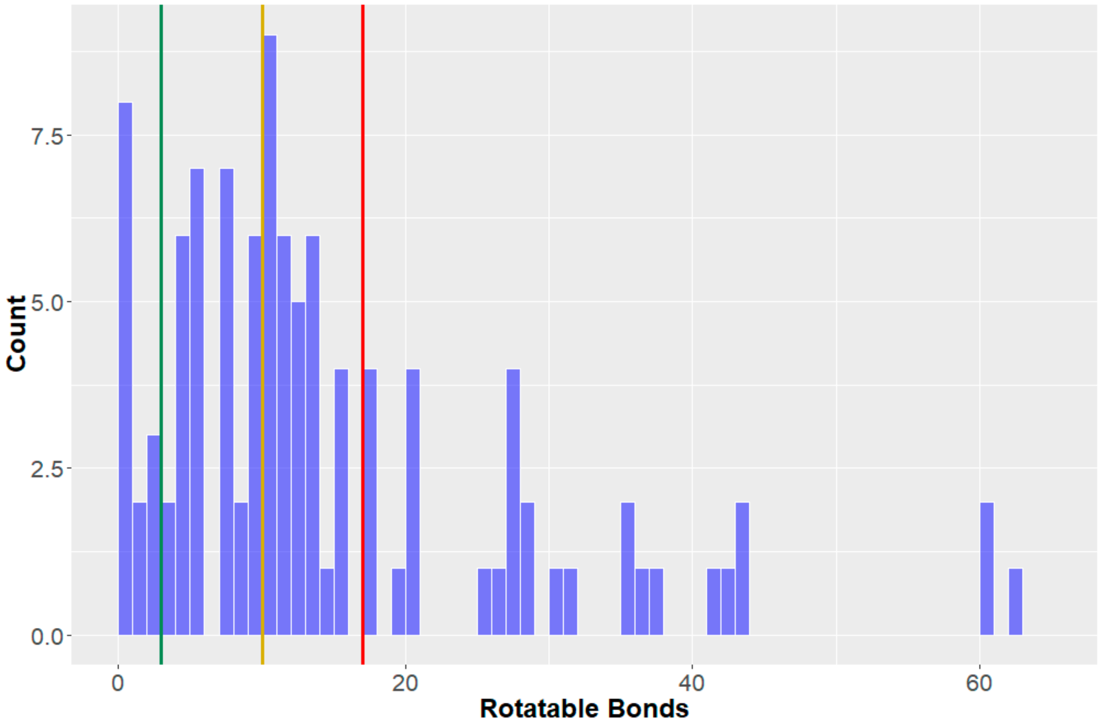

Figure S6. The statistical distribution of the rotatable bonds of all analysed compounds isolated from organisms in the Animalia Kingdom (green = 3, compounds < 3 are in the lead-like space; yellow = 10, compounds < 10 are in the drug-like space; red= 17, compounds < 17 are in the known drug space. Total number of compounds = 104.

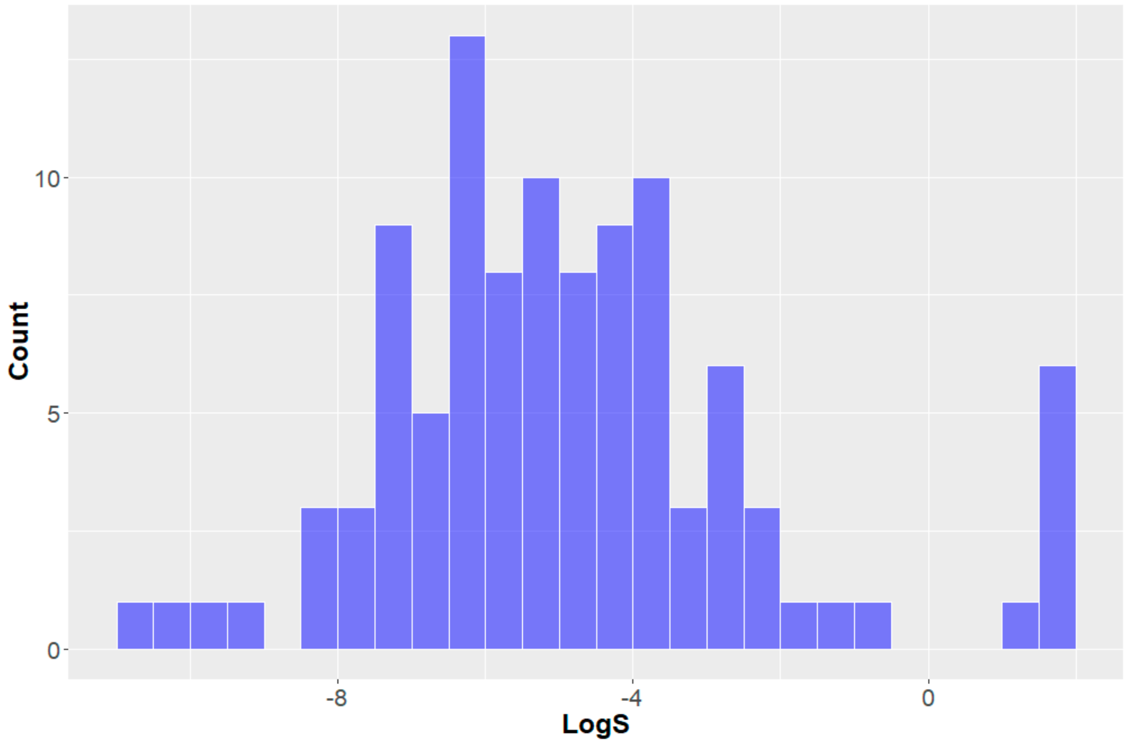

Figure S7. The statistical distribution of the LogS of all analysed compounds isolated from organisms in the Animalia Kingdom. Total number of compounds = 104.

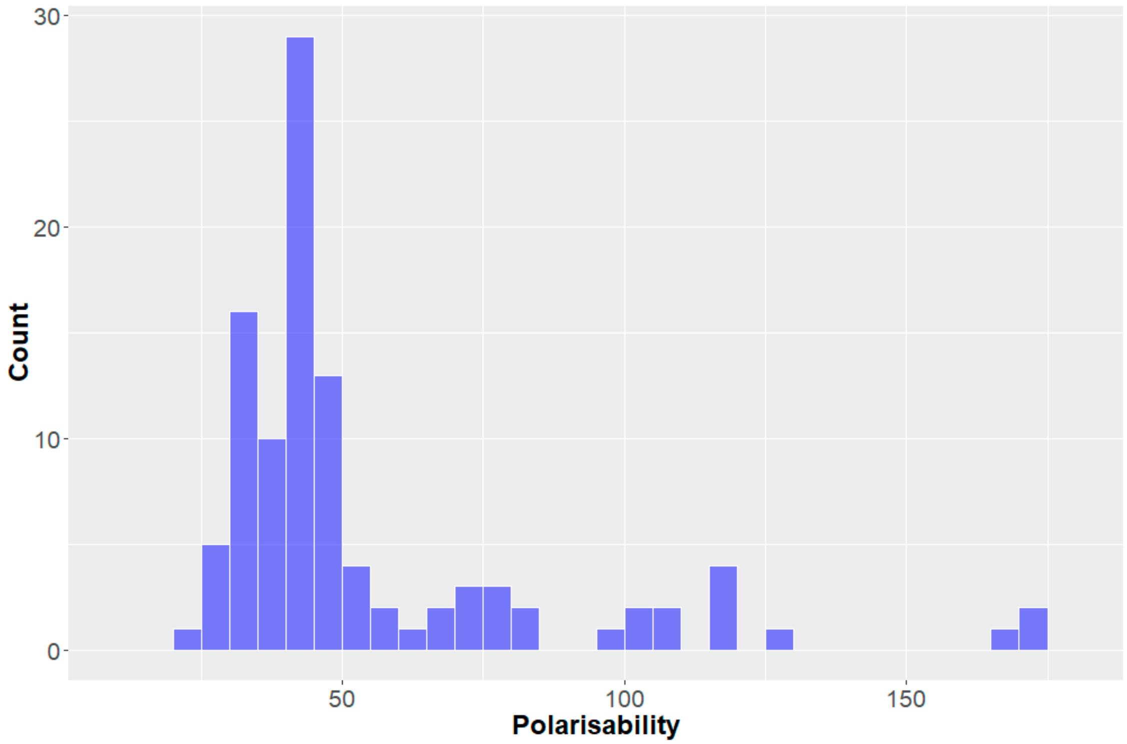

Figure S8. The statistical distribution of the polarisability of all analysed compounds isolated from organisms in the Animalia Kingdom. Total number of compounds = 104.

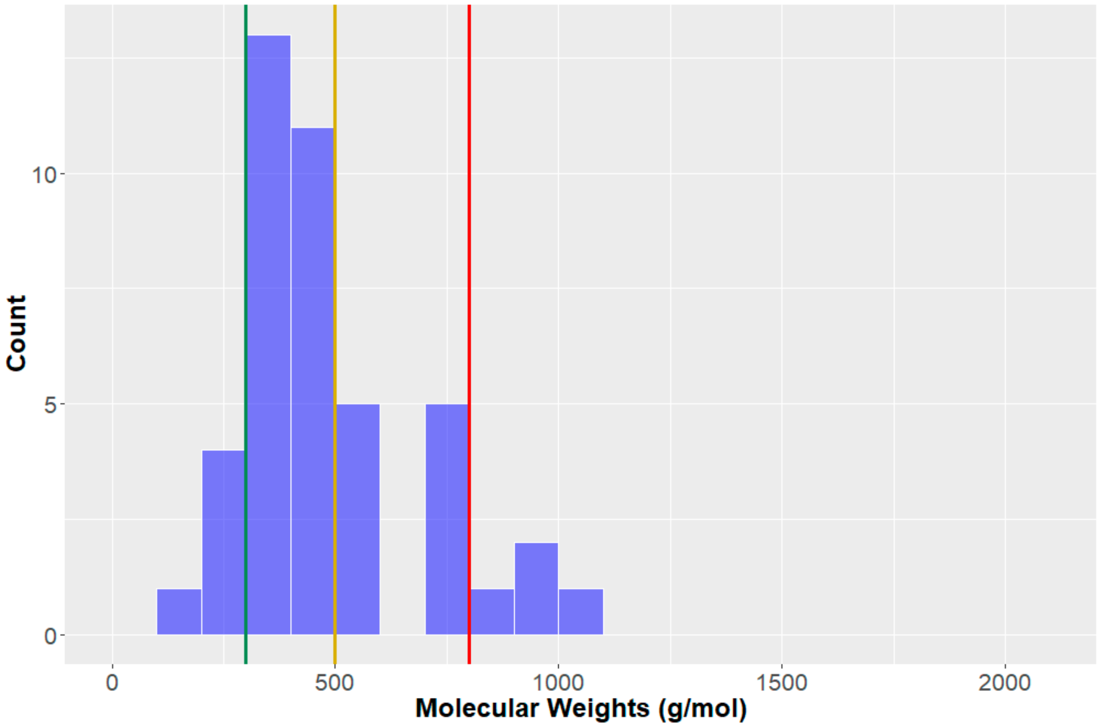

Figure S9. The statistical distribution of the molecular weight of analysed compounds isolated from organisms in the Bacteria Kingdom (green = 300 g mol^{-1}, compounds < 300 g $mol^{-1}$are in the *lead-like* space; yellow = 500 g mol^{-1}, compounds < 500 g mol^{-1} are in the *drug-like* space; red= 800 g mol^{-1}, compounds < 800 g $mol^{-1}$are in the KDS. Total number of compounds = 43.

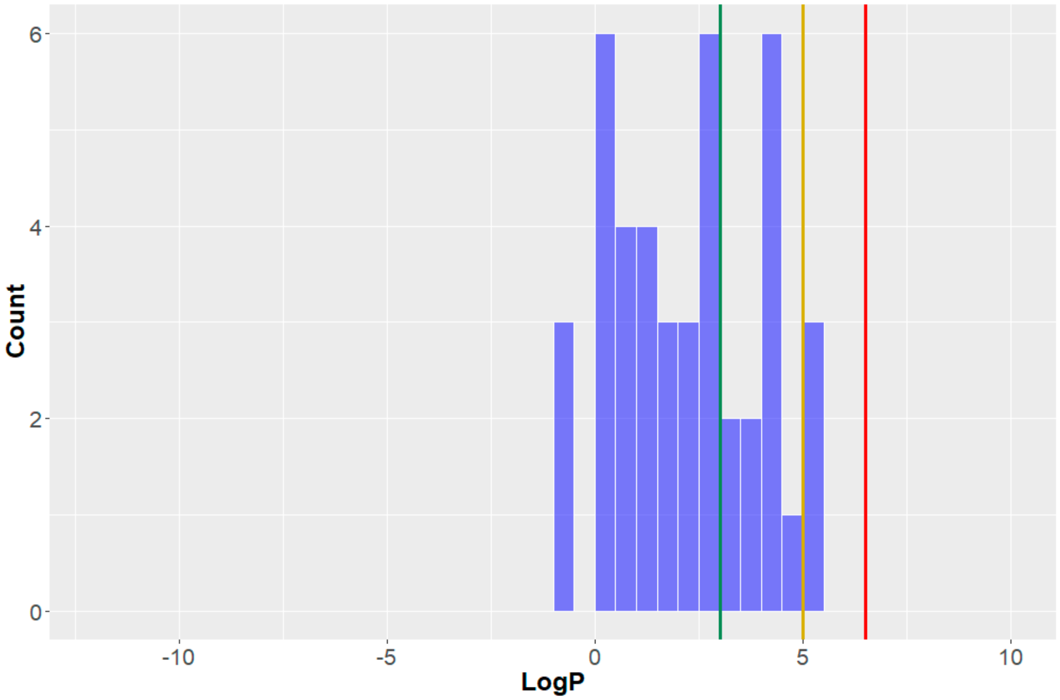

Figure S10. The statistical distribution of the octanol – water partition coefficient (LogP) of analysed compounds isolated from organisms in the Bacteria Kingdom (green = 3, compounds < 3 are in the *lead-like* space; yellow = 5, compounds < 5 are in the *drug-like* space; red= 6.5, compounds < 6.5 are in the KDS. Total number of compounds = 43.

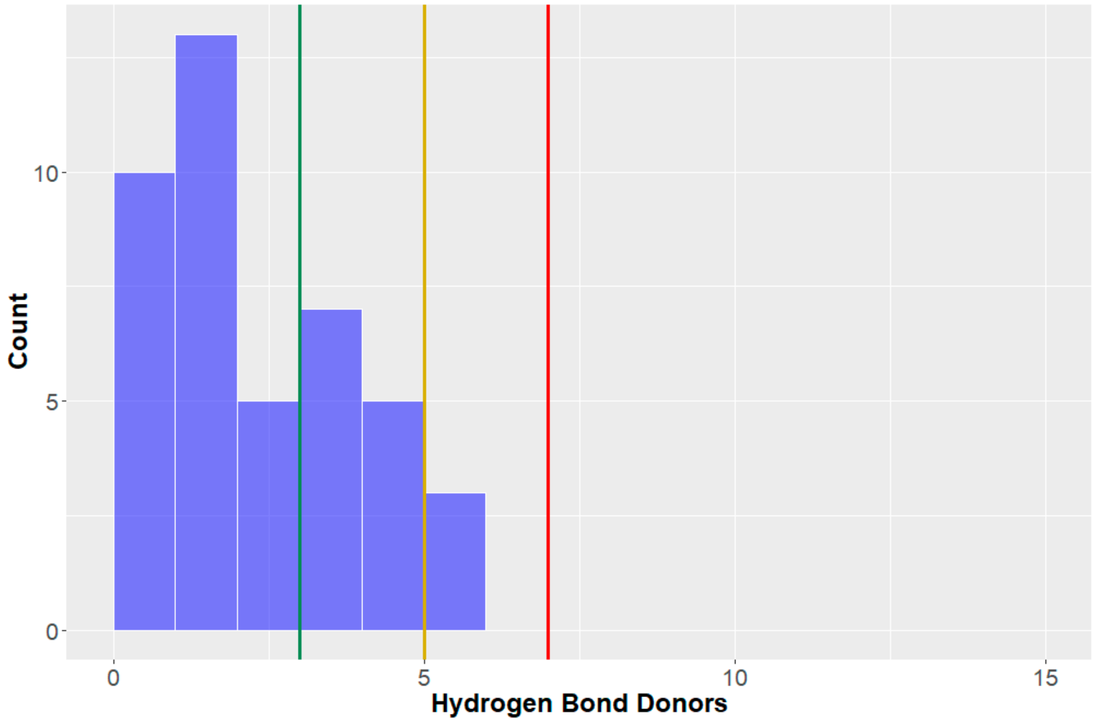

Figure S11. The statistical distribution of the hydrogen bond donors of analysed compounds isolated from organisms in the Bacteria Kingdom (green = 3, compounds < 3 are in the *lead-like* space; yellow = 5, compounds < 5 are in the *drug-like* space; red= 7, compounds < 7 are in the KDS. Total number of compounds = 43.

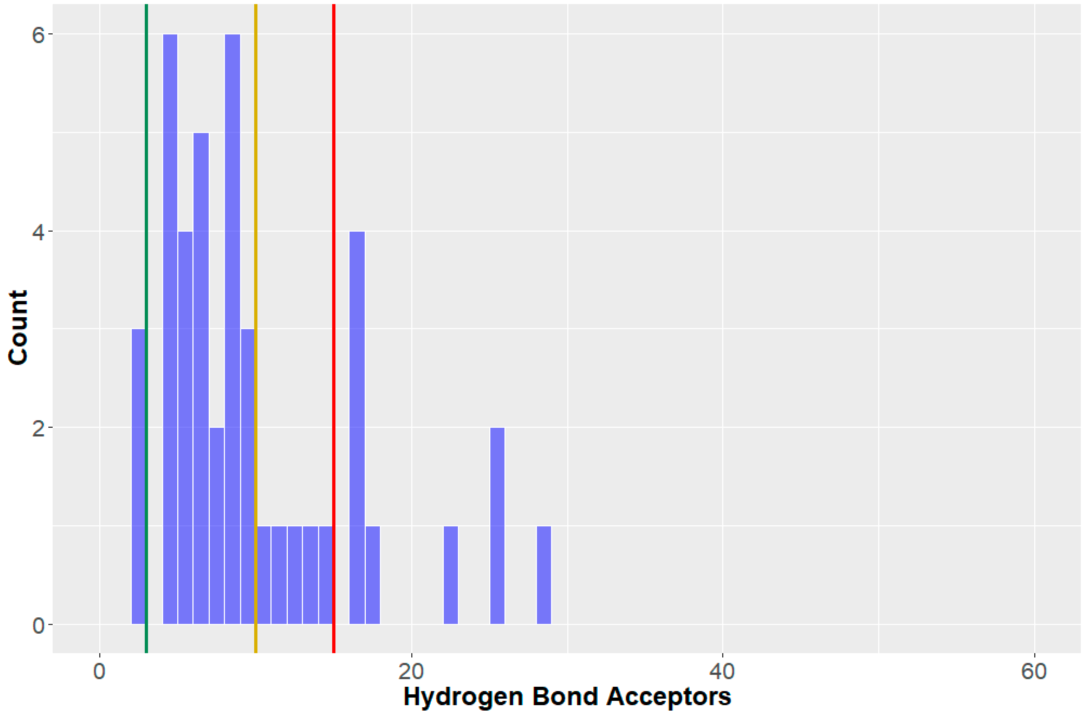

Figure S12. The statistical distribution of the hydrogen bond acceptors of analysed compounds isolated from organisms in the Bacteria Kingdom (green = 3, compounds < 3 are in the *lead-like* space; yellow = 5, compounds < 5 are in the *drug-like* space; red = 15, compounds < 15 are in the KDS. Total number of compounds = 43.

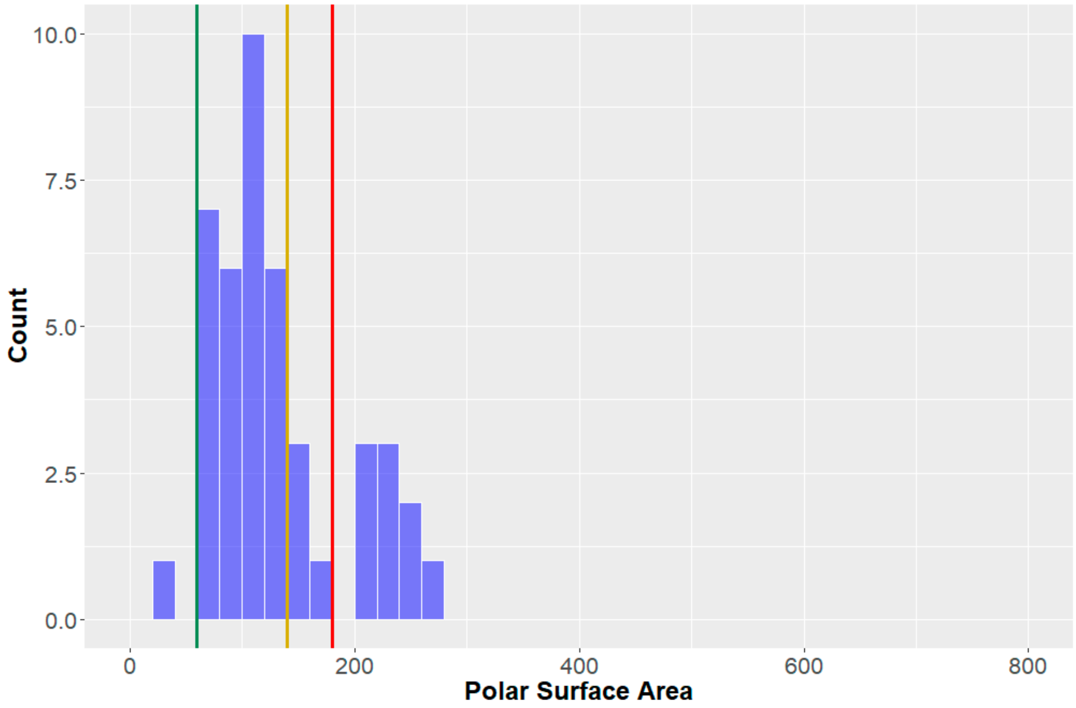

Figure S13. The statistical distribution of the polar surface area (PSA) of analysed compounds isolated from organisms in the Bacteria Kingdom (green = 60, compounds < 60 Å^2 are in the *lead-like* space; yellow = 140, compounds < 140 Å^2 are in the *drug-like* space; red= 180, compounds < 180 Å^2 are in the KDS. Total number of compounds = 43.

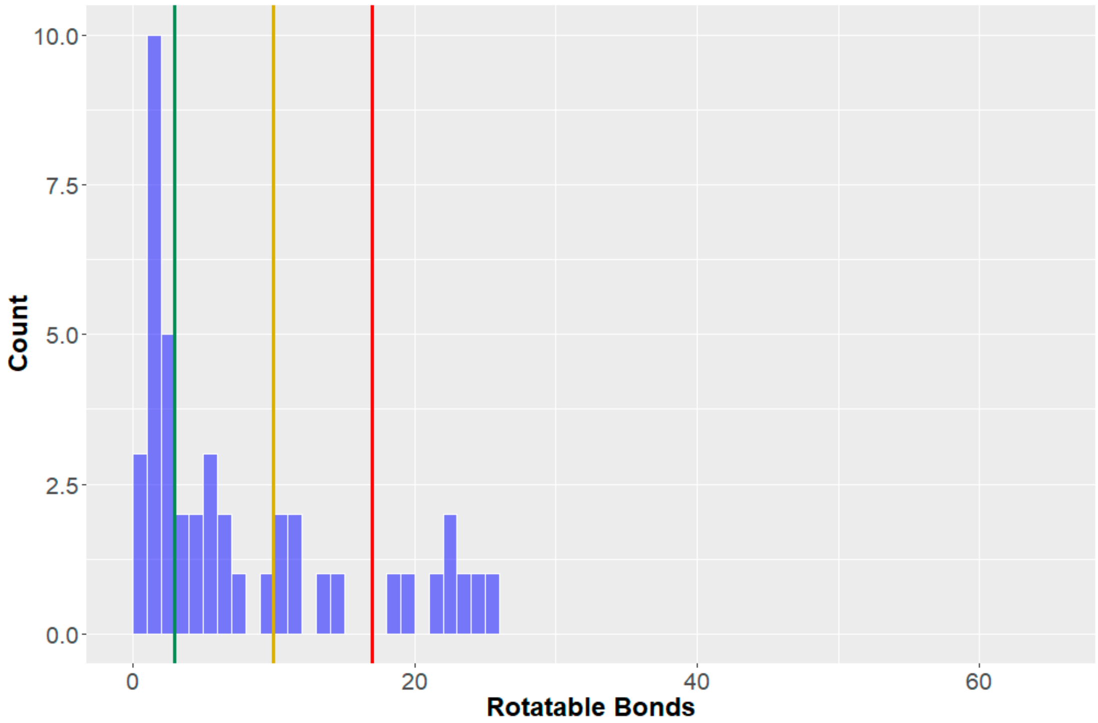

Figure S14. The statistical distribution of the rotatable bonds of all analysed compounds isolated from organisms in the Bacteria Kingdom (green = 3, compounds < 3 are in the lead-like space; yellow = 10, compounds < 10 are in the drug-like space; red= 17, compounds < 17 are in the known drug space. Total number of compounds = 43.

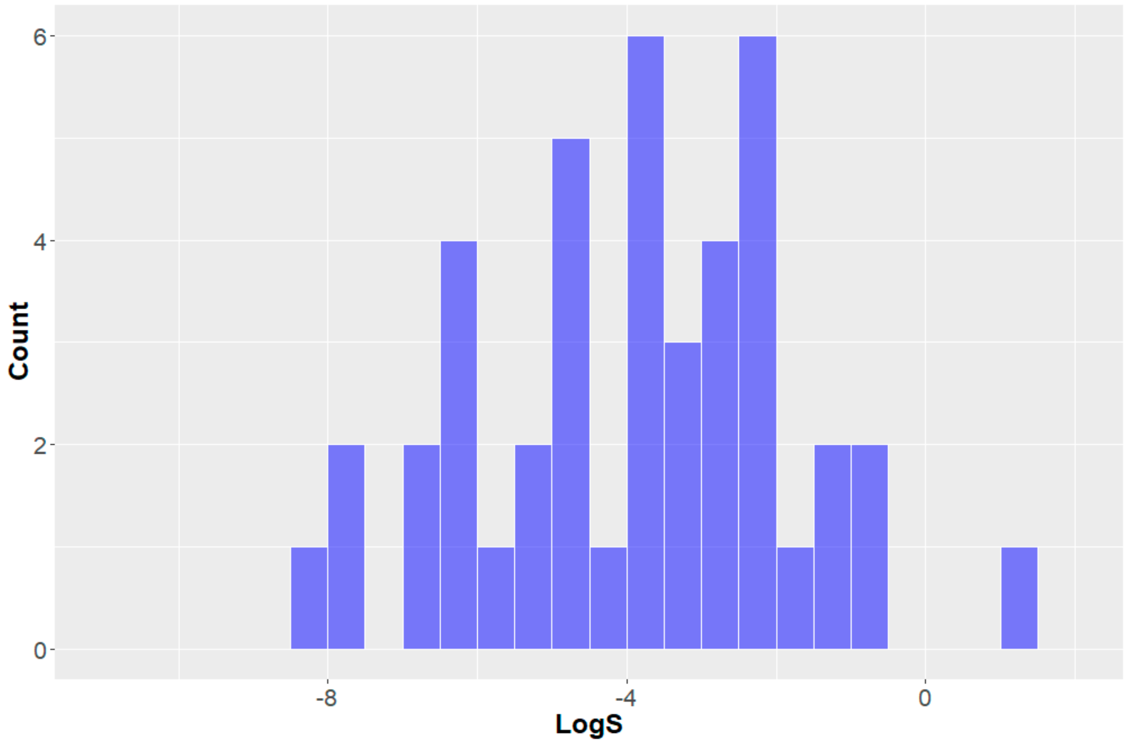

Figure S15. The statistical distribution of the LogS of all analysed compounds isolated from organisms in the Bacteria Kingdom. Total number of compounds = 43.

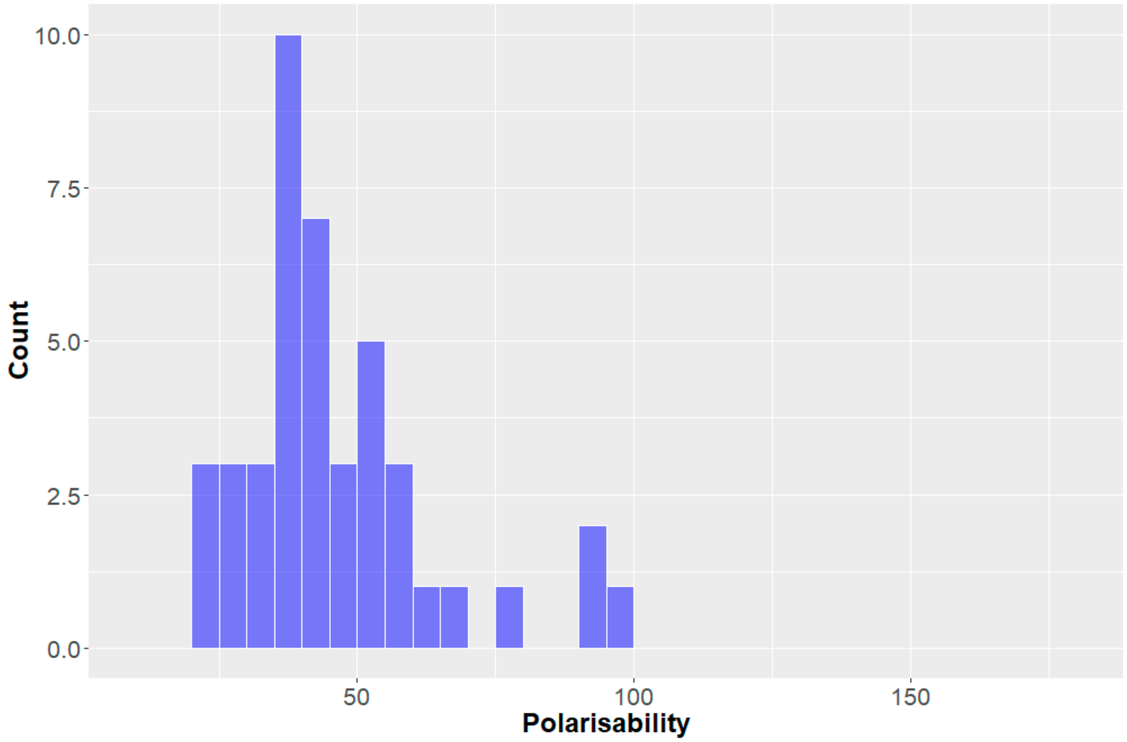

Figure S16. The statistical distribution of the polarisability of all analysed compounds isolated from organisms in the Bacteria Kingdom. Total number of compounds = 43.

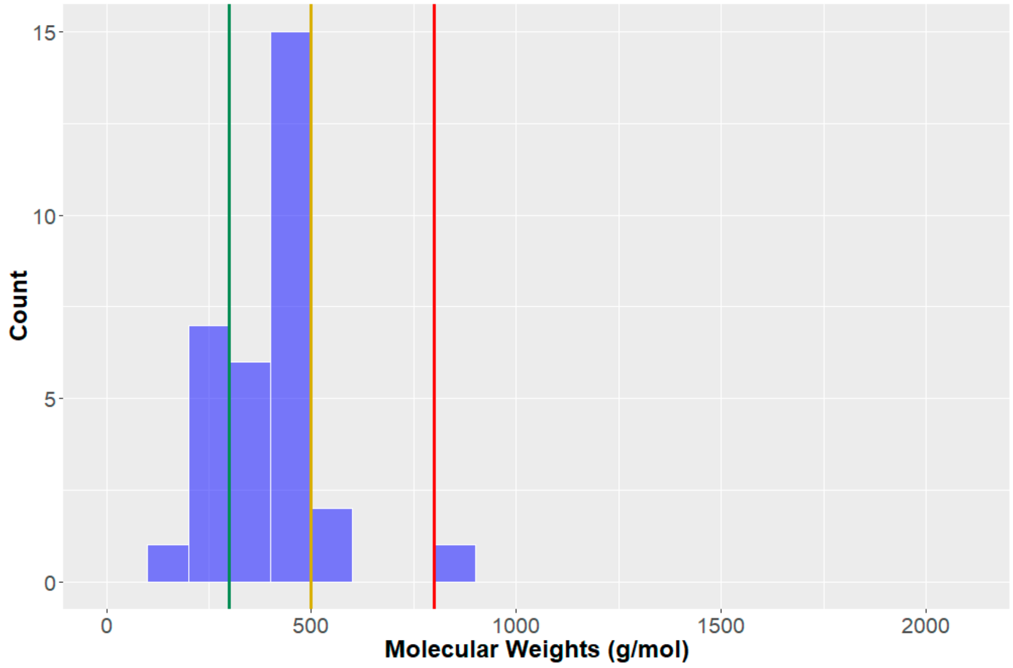

Figure S17. The statistical distribution of the molecular weight of analysed compounds isolated from organisms in the Fungi Kingdom (green = 300 g mol^{-1}, compounds < 300 g $mol^{-1}$are in the *lead-like* space; yellow = 500 g mol^{-1}, compounds < 500 g mol^{-1} are in the *drug-like* space; red= 800 g mol^{-1}, compounds < 800 g $mol^{-1}$are in the KDS. Total number of compounds = 32.

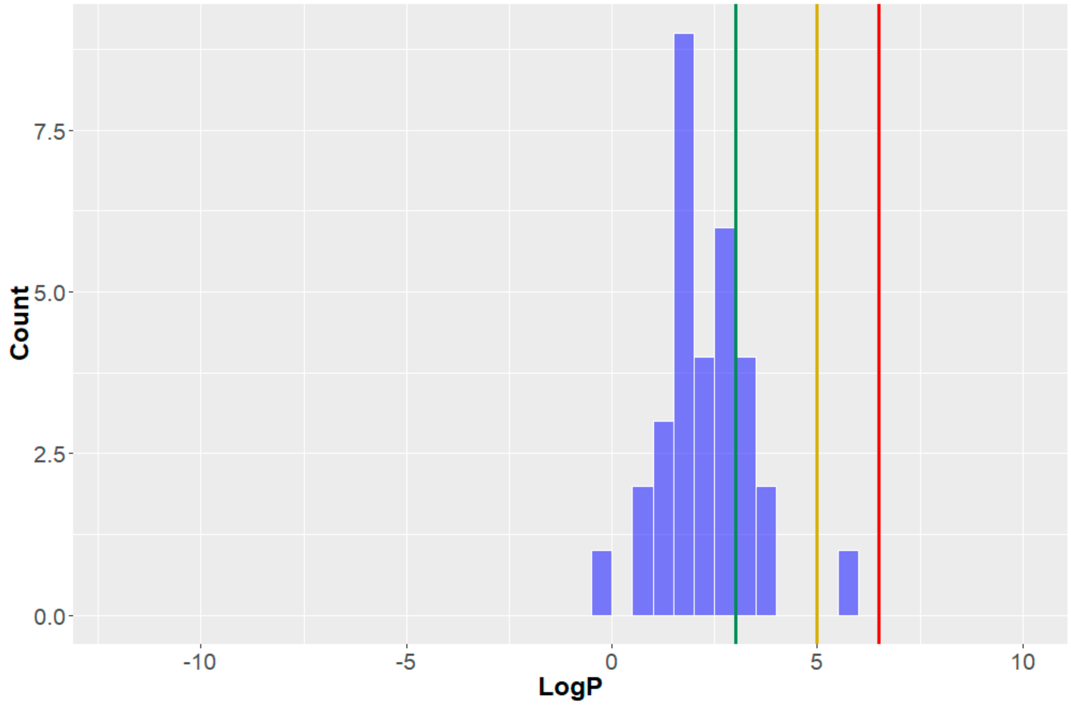

Figure S18. The statistical distribution of the octanol – water partition coefficient (LogP) of analysed compounds isolated from organisms in the Bacteria Kingdom (green = 3, compounds < 3 are in the *lead-like* space; yellow = 5, compounds < 5 are in the *drug-like* space; red= 6.5, compounds < 6.5 are in the KDS. Total number of compounds = 32.

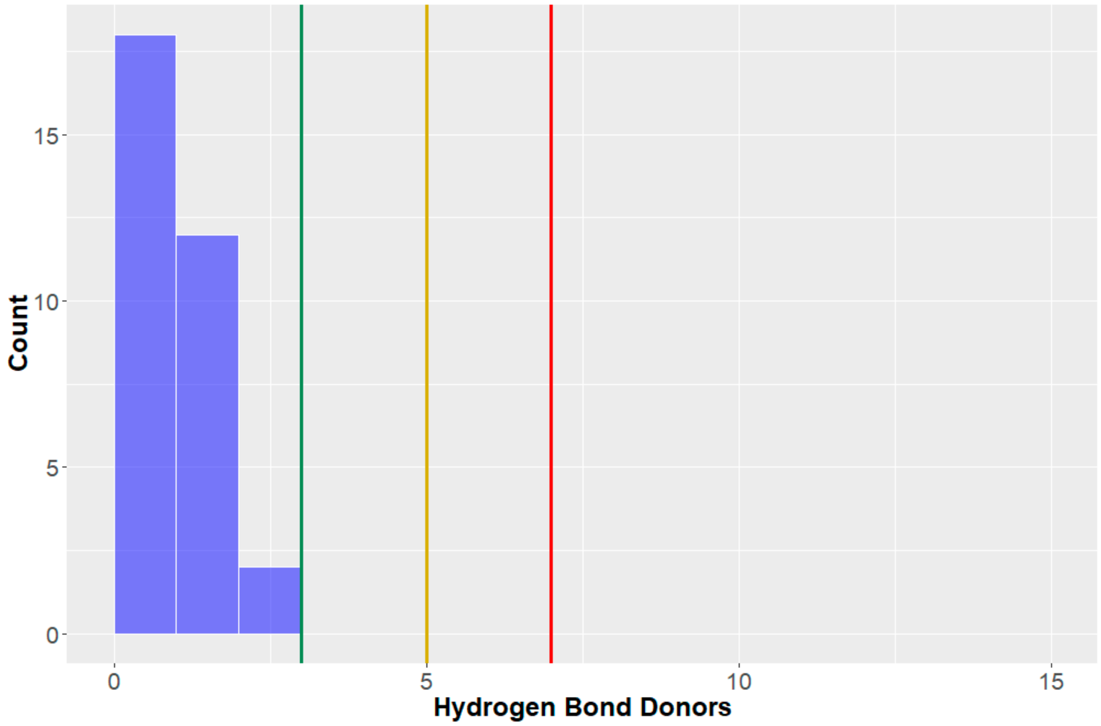

Figure S19. The statistical distribution of the hydrogen bond donors of analysed compounds isolated from organisms in the Fungi Kingdom (green = 3, compounds < 3 are in the *lead-like* space; yellow = 5, compounds < 5 are in the *drug-like* space; red= 7, compounds < 7 are in the KDS. Total number of compounds = 32.

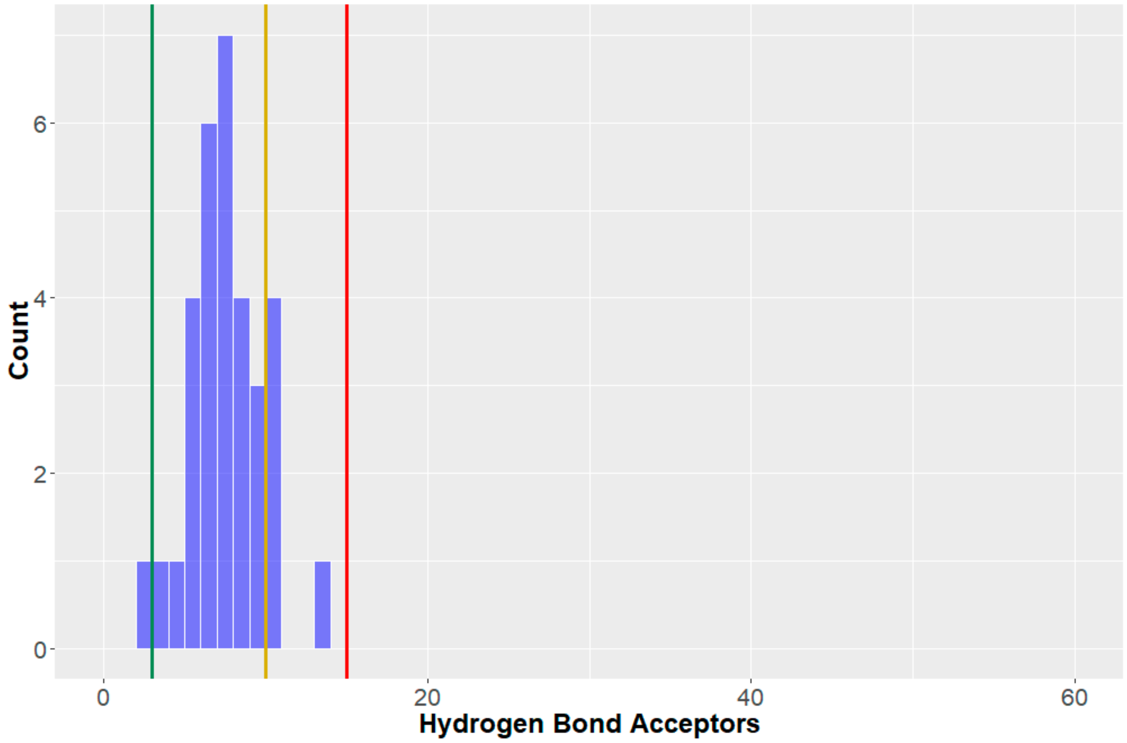

Figure S20. The statistical distribution of the hydrogen bond acceptors of analysed compounds isolated from organisms in the Fungi Kingdom (green = 3, compounds < 3 are in the *lead-like* space; yellow = 5, compounds < 5 are in the *drug-like* space; red = 15, compounds < 15 are in the KDS. Total number of compounds = 32.

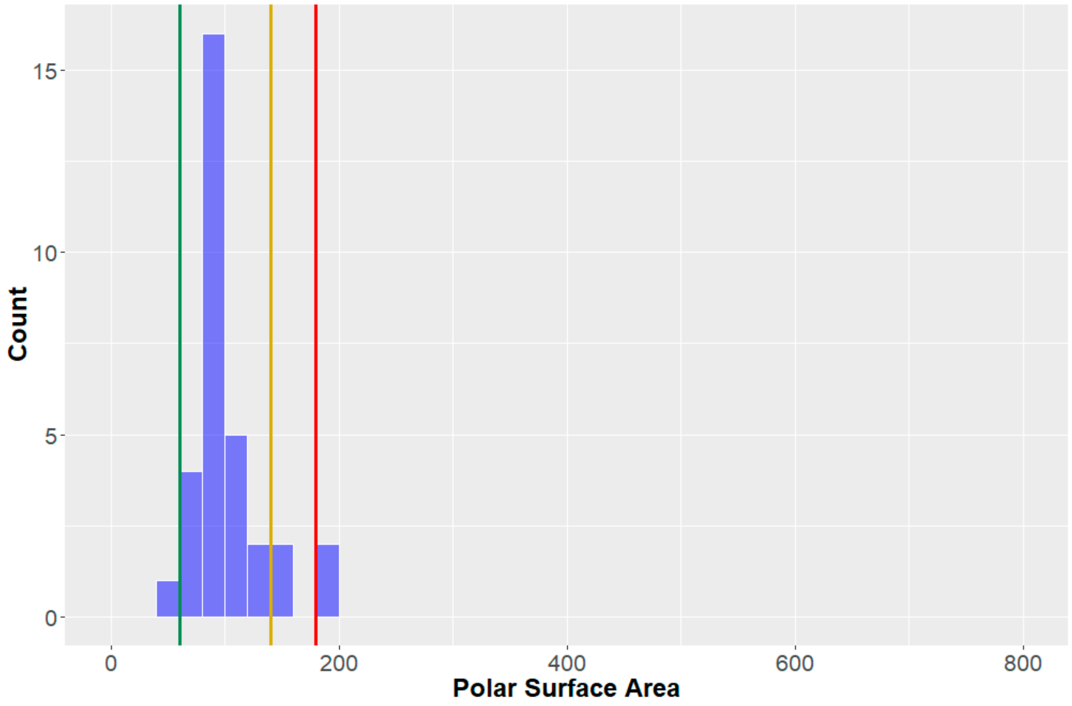

Figure S21. The statistical distribution of the polar surface area (PSA) of analysed compounds isolated from organisms in the Fungi Kingdom (green = 60, compounds < 60 Å^2 are in the *lead-like* space; yellow = 140, compounds < 140 Å^2 are in the *drug-like* space; red= 180, compounds < 180 Å^2 are in the KDS. Total number of compounds = 32.

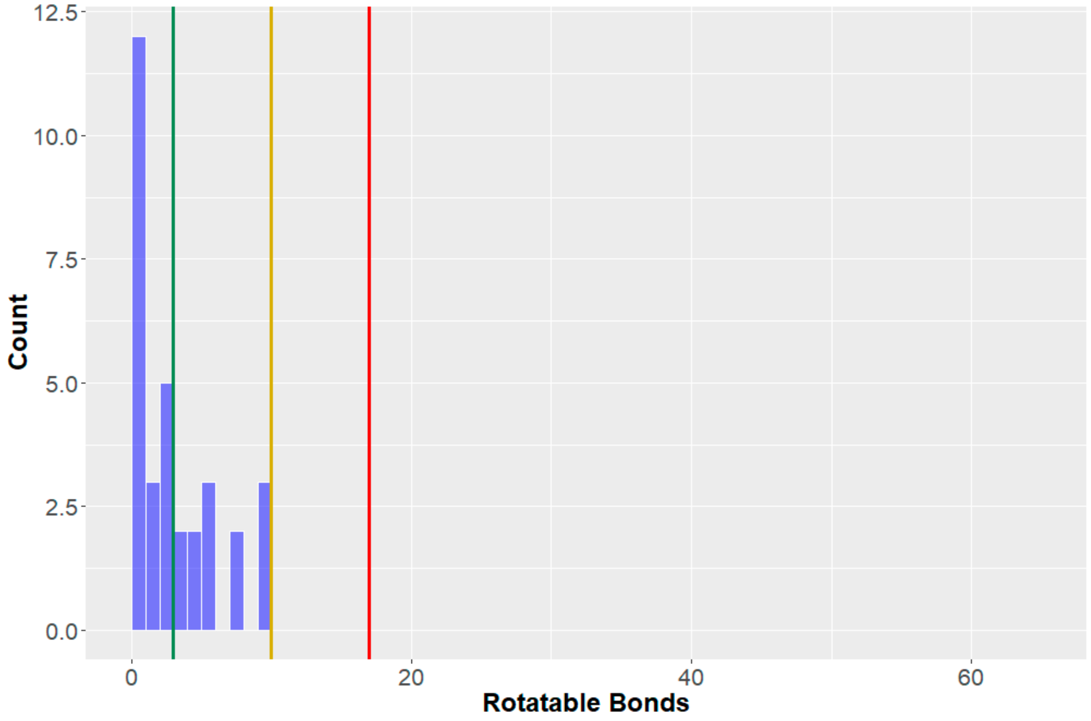

Figure S22. The statistical distribution of the rotatable bonds of all analysed compounds isolated from organisms in the Fungi Kingdom (green = 3, compounds < 3 are in the lead-like space; yellow = 10, compounds < 10 are in the drug-like space; red= 17, compounds < 17 are in the known drug space. Total number of compounds = 32.

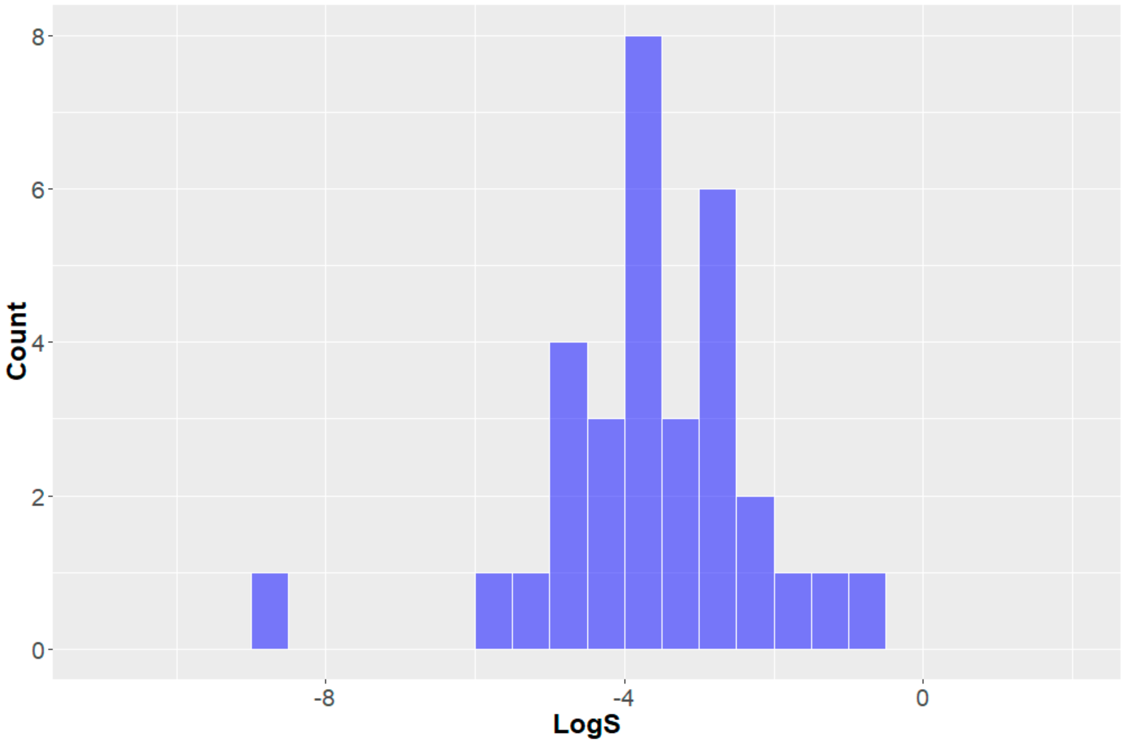

Figure S23. The statistical distribution of the LogS of all analysed compounds isolated from organisms in the Fungi Kingdom. Total number of compounds = 32.

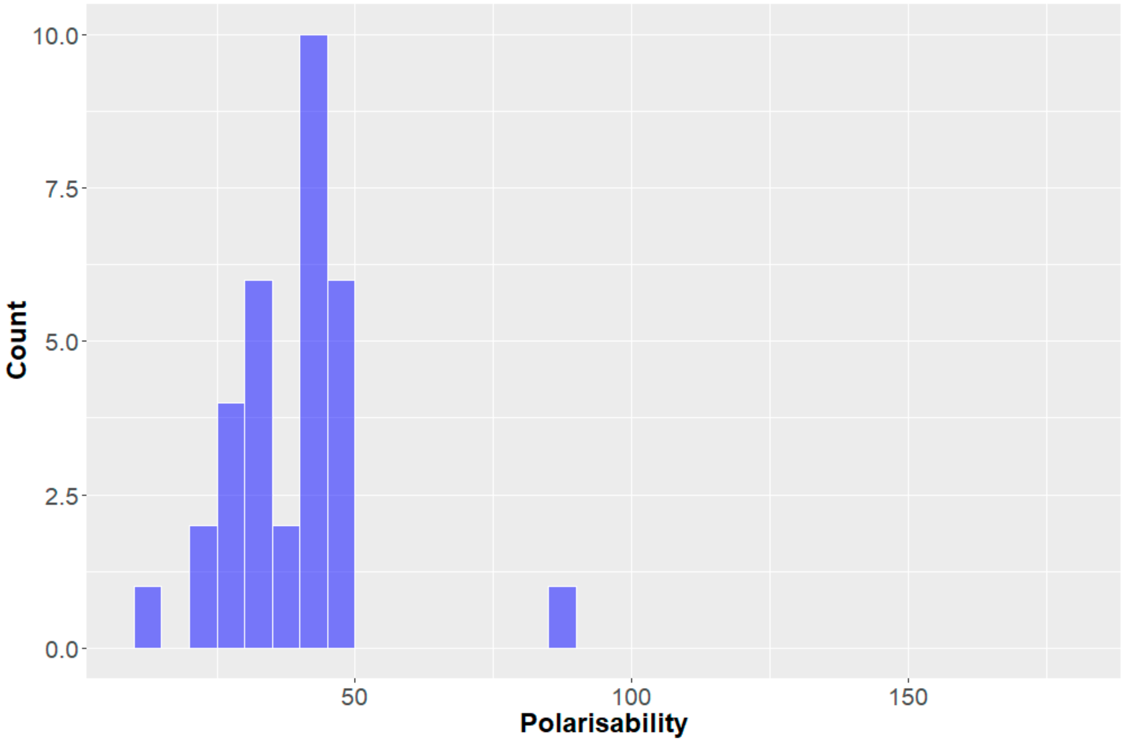

Figure S24. The statistical distribution of the polarisability of all analysed compounds isolated from organisms in the Fungi Kingdom. Total number of compounds = 32.

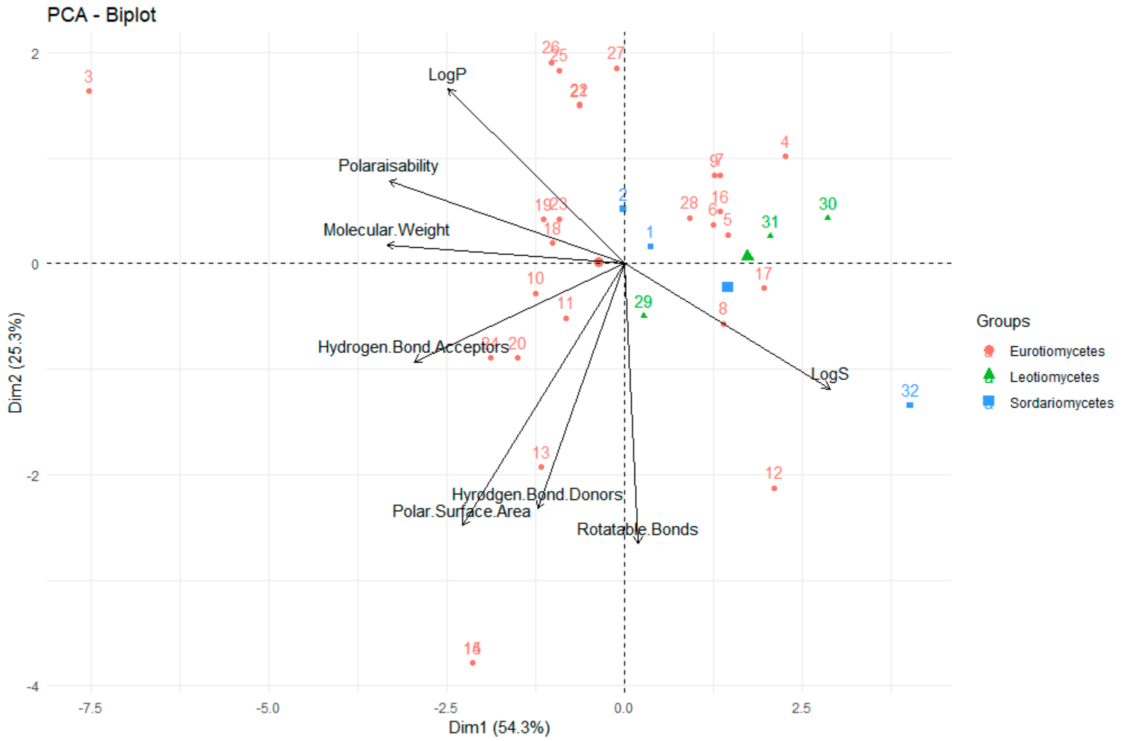

Figure S25: Biplot representing the PCA analysis on the compounds isolated from organisms in the Fungi Kingdom and their molecular descriptors (PC1 vs PC2). The arrows represent molecular descriptors and the direction in which they hold influence. Each point represents a molecule.

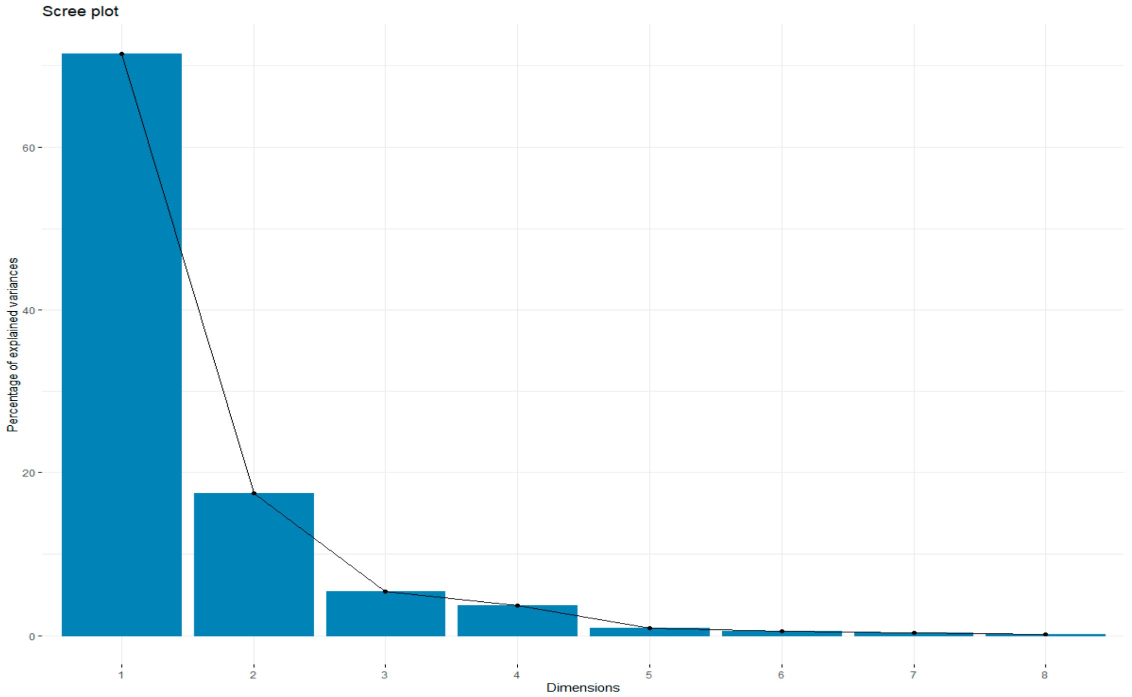

Figure S26: Scree representing the PCA analysis on all the studied compounds from all Kingdoms.

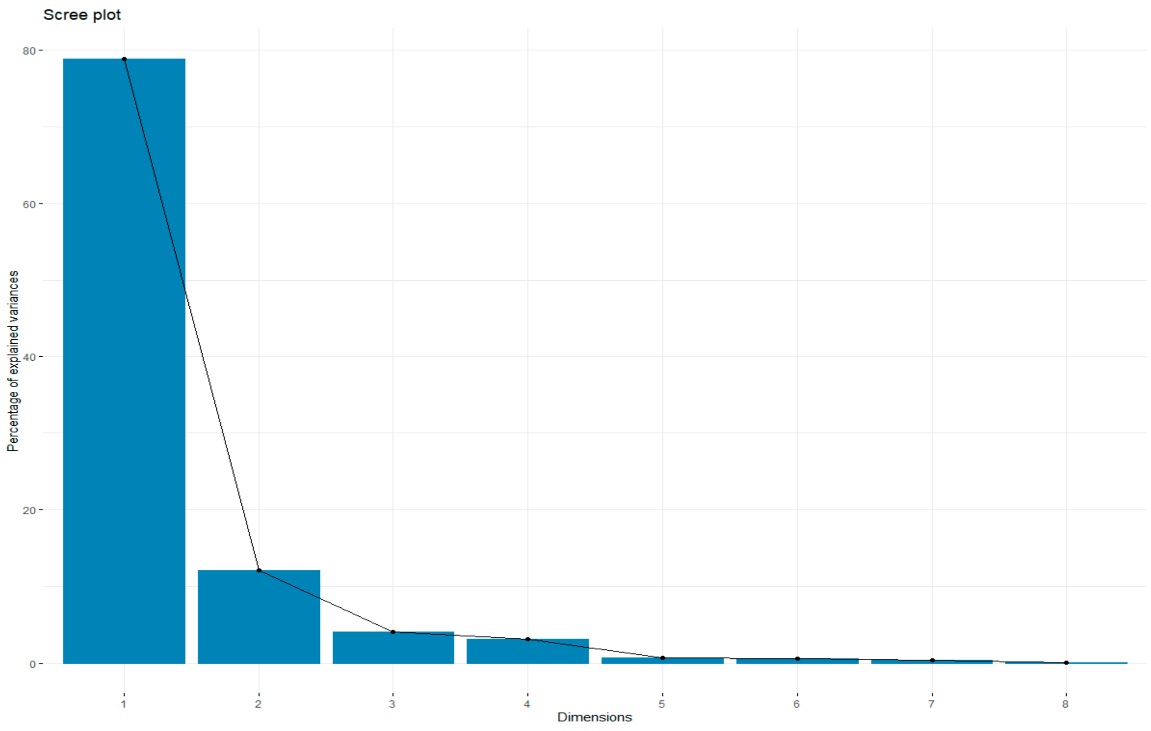

Figure S27: Scree plot representing the PCA analysis on the compounds isolated from organisms from the Animalia Kingdom.

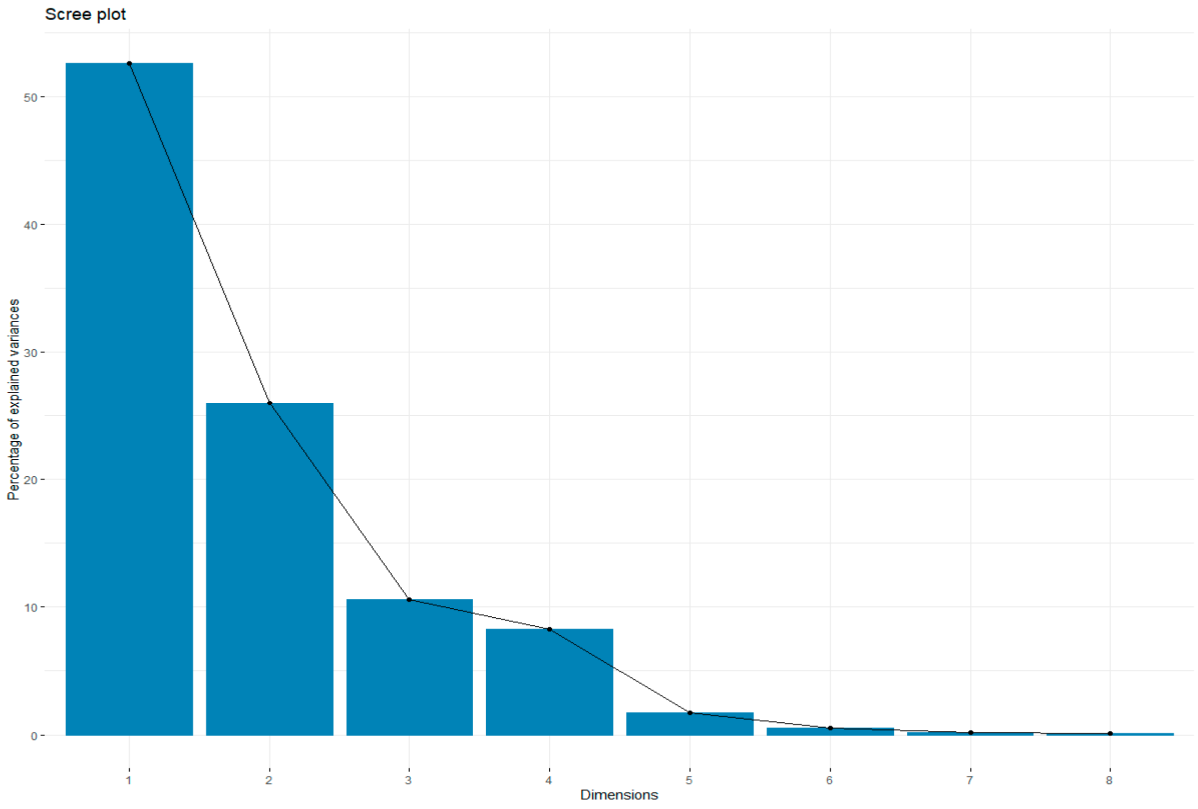

Figure S28: Scree plot representing the PCA analysis on the compounds isolated from organisms from the Bacteria Kingdom.

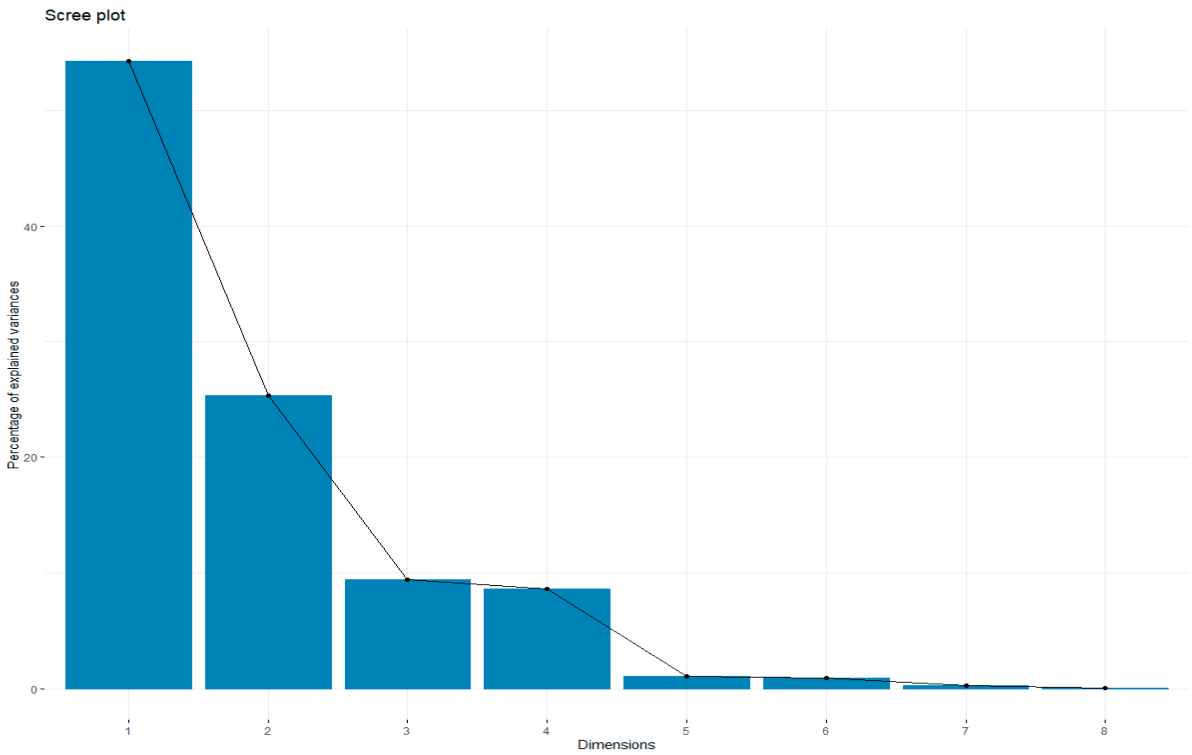

Figure S29: Scree plot representing the PCA analysis on the compounds isolated from organisms from the Fungi Kingdom.

Table S1: Recently-isolated Deep Sea compounds analysed in this study, their identifiers, species isolated from and references.

Title	CAS number	Name	Kingdom	Phylum	Class	Species	Ref.
**DS1**	1350430-04-4	Convolutamine I	Animalia	Bryozoa	Ctenostomata	*Amathia tortusa*	1
**DS3**	1196466-12-2	Rossinone A	Animalia	Chordata	Ascidiacea	*Aplidium* sp.	2
**DS4**	1196466-13-3	Rossinone B	Animalia	Chordata	Ascidiacea	*Aplidium* sp.	2
**DS5**	1333876-25-7	Cristaxencin A	Animalia	Cnidaria	Anthozoa	*Acanthoprimnoa cristata*	3
**DS6**	1248699-03-7	5(1*H*)-Cyclopentacycloundecenone, 2,3,3a,4,8,9,12,12a-octahydro-6,10,12a-trimethyl-3-(1-methylethenyl)-, (3*R*,3a*S*,6*E*,10*E*,12a*R*)-	Animalia	Cnidaria	Anthozoa	*Convexella magelhaenica*	4
**DS7**	1248699-06-0	5(1*H*)-Cyclopentacycloundecenone, 2,12-bis(acetyloxy)-2,3,3a,4,8,9,12,12a-octahydro-6,10,12a-trimethyl-3-(1-methylethenyl)-, (2*S*,3*R*,3a*S*,6*E*,10*E*,12*R*,12a*S*)-	Animalia	Cnidaria	Anthozoa	*Convexella magelhaenica*	4
**DS8**	1255215-72-5	Pseudozoanthoxanthin III	Animalia	Cnidaria	Anthozoa	*Echinogorgia pseudossapo*	5
**DS9**	1255215-73-6	Pseudozoanthoxanthin IV	Animalia	Cnidaria	Anthozoa	*Echinogorgia pseudossapo*	5
**DS10**	1370347-77-5	2H-Indeno[5,4-*b*]furan-2-one, 8-acetyl-3a,4,5,5a,6,7,8,8a-octahydro-5-hydroxy-3a-methoxy-1,5-dimethyl-, (3a*S*,5*R*,5a*R*,8*S*,8a*S*)-	Animalia	Cnidaria	Anthozoa	*Echinogorgia pseudossapo*	5
**DS11**	1370347-78-6	1-Azulenol, decahydro-2-methoxy-3-methyl-8-methylene-5-(1-methylethyl)-, (1*S*,2*R*,3*S*,3a*S*,5*R*,8a*R*)-	Animalia	Cnidaria	Anthozoa	*Echinogorgia pseudossapo*	5

**DS12**	1219114-43-8	Gymnochrome E	Animalia	Echinodermata	Crinoidea	*Holopus rangii*	6
**DS13**	137490-26-7	Gymnochrome F	Animalia	Echinodermata	Crinoidea	*Holopus rangii*	6
**DS14**	1215074-98-8	7-Bromoemodic acid	Animalia	Echinodermata	Crinoidea	*Holopus rangii*	6
**DS15**	1192037-26-5	Proisocrinin A	Animalia	Echinodermata	Crinoidea	*Proisocrinus ruberrimus*	7
**DS16**	1192037-31-2	Proisocrinin B	Animalia	Echinodermata	Crinoidea	*Proisocrinus ruberrimus*	7
**DS17**	1192037-35-6	Proisocrinin C	Animalia	Echinodermata	Crinoidea	*Proisocrinus ruberrimus*	7
**DS18**	1192037-39-0	Proisocrinin D	Animalia	Echinodermata	Crinoidea	*Proisocrinus ruberrimus*	7
**DS19**	1192037-43-6	Proisocrinin E	Animalia	Echinodermata	Crinoidea	*Proisocrinus ruberrimus*	7
**DS20**	1192037-45-8	Proisocrinin F	Animalia	Echinodermata	Crinoidea	*Proisocrinus ruberrimus*	7
**DS21**	1094279-52-3	Achlioniceoside A1	Animalia	Echinodermata	Holothuroidea	*Achlionice violaecuspidata*	8
**DS22**	1094279-54-5	Achlioniceoside A2	Animalia	Echinodermata	Holothuroidea	*Achlionice violaecuspidata*	8
**DS23**	1094279-56-7	Achlioniceoside A3	Animalia	Echinodermata	Holothuroidea	*Achlionice violaecuspidata*	8
**DS24**	1421689-98-6	Lipoamicoumasin A	Bacteria	Firmacutes	Bacilli	*Bacillus subtilis*	9
**DS25**	1422363-40-3	Lipoamicoumasin B	Bacteria	Firmacutes	Bacilli	*Bacillus subtilis*	9
**DS26**	1421689-99-7	Lipoamicoumasin C	Bacteria	Firmacutes	Bacilli	*Bacillus subtilis*	9
**DS27**	1422363-41-4	Lipoamicoumasin D	Bacteria	Firmacutes	Bacilli	*Bacillus subtilis*	9
**DS28**	1421690-00-7	Bacilosarcin C	Bacteria	Firmacutes	Bacilli	*Bacillus subtilis*	9
**DS29**	1233490-98-6	Dermacozine A	Bacteria	Actinobacteria	Actinobacteria	*Dermacoccus abyssi*	10
**DS30**	1233490-99-7	Dermacozine B	Bacteria	Actinobacteria	Actinobacteria	*Dermacoccus abyssi*	10
**DS31**	1233491-00-3	Dermacozine C	Bacteria	Actinobacteria	Actinobacteria	*Dermacoccus abyssi*	10
**DS32**	1233491-01-4	Dermacozine D	Bacteria	Actinobacteria	Actinobacteria	*Dermacoccus abyssi*	10
**DS33**	1233491-03-6	Dermacozine E	Bacteria	Actinobacteria	Actinobacteria	*Dermacoccus abyssi*	10

**DS34**	1233491-04-7	Dermacozine F	Bacteria	Actinobacteria	Actinobacteria	*Dermacoccus abyssi*	10
**DS35**	1233491-05-8	Dermacozine G	Bacteria	Actinobacteria	Actinobacteria	*Dermacoccus abyssi*	10
**DS36**	1334143-01-9	Acetamide, *N*-[2-[4-(acetylamino) phenyl]-1-(hydroxymethyl)-2-(1*H*-indol-3-yl)ethyl]-2,2-dichloro-, (+)-	Bacteria	Proteobacteria	Gammaproteobacteria	Clone-derived *E. coli*	11
**DS37**	1338578-37-2	Marinacarboline A	Bacteria	Actinobacteria	Actinobacteria	*Marinactinospora thermotolerans*	12
**DS38**	1338578-38-3	Marinacarboline B	Bacteria	Actinobacteria	Actinobacteria	*Marinactinospora thermotolerans*	12
**DS39**	1338578-39-4	Marinacarboline C	Bacteria	Actinobacteria	Actinobacteria	*Marinactinospora thermotolerans*	12
**DS40**	1338578-40-7	Marinacarboline D	Bacteria	Actinobacteria	Actinobacteria	*Marinactinospora thermotolerans*	12
**DS41**	1338474-29-5	3*H*-Pyrrolo[4,3,2-*gh*]-1,4-benzodiazonin-3-one, 9-(1,1-dimethyl-2-propen-1-yl)-1,2,4,5,6,8-hexahydro-5-(hydroxymethyl)-2-[(1*S*)-1-methylpropyl]-, (2*S*,5*S*)-	Bacteria	Actinobacteria	Actinobacteria	*Marinactinospora thermotolerans*	12
**DS42**	1338474-30-8	3*H*-Pyrrolo[4,3,2-*gh*]-1,4-benzodiazonin-3-one, 9-(1,1-dimethyl-2-propen-1-yl)-5-[(α-D-glucopyranosyloxy)methyl]-1,2,4,5,6,8-hexahydro-1-methyl-2-[(1*S*)-1-methylpropyl]-, (2*S*,5*S*)-	Bacteria	Actinobacteria	Actinobacteria	*Marinactinospora thermotolerans*	12
**DS43**	1334304-56-1	Levantilide A	Bacteria	Actinobacteria	Actinomycetales	*Micromonospora* sp.	13
**DS44**	1334304-57-2	Levantilide B	Bacteria	Actinobacteria	Actinomycetales	*Micromonospora* sp.	13
**DS45**	1225203-42-8	Nocardiopsin A	Bacteria	Actinobacteria	Actinobacteria	*Nocardiopsis* sp.	14
**DS46**	1225203-43-9	Nocardiopsin B	Bacteria	Actinobacteria	Actinobacteria	*Nocardiopsis* sp.	14
**DS47**	1300744-64-2	Nocardioazine A	Bacteria	Actinobacteria	Actinobacteria	*Nocardiopsis* sp.	15
**DS48**	1300744-65-3	Nocardioazine B	Bacteria	Actinobacteria	Actinobacteria	*Nocardiopsis* sp.	15

**DS49**	1354010-43-7	Pseudocardian A	Bacteria	Actinobacteria	Actinobacteria	*Pseudonocardia* sp.	16
**DS50**	1354010-45-9	Pseudocardian B	Bacteria	Actinobacteria	Actinobacteria	*Pseudonocardia* sp.	16
**DS51**	1354010-46-0	Pseudocardian C	Bacteria	Actinobacteria	Actinobacteria	*Pseudonocardia* sp.	16
**DS52**	1487410-38-7	1*H*-Indole, 2-[(6-methyl-2-pyrazinyl)methyl]-	Bacteria	Actinobacteria	Actinobacteria	*Serinicoccus profundi*	17
**DS53**	1358965-79-3	Grincamycin B	Bacteria	Actinobacteria	Actinobacteria	*Streptomyces lusitanus*	18
**DS54**	1358965-81-7	Grincamycin C	Bacteria	Actinobacteria	Actinobacteria	*Streptomyces lusitanus*	18
**DS55**	1358965-83-9	Grincamycin D	Bacteria	Actinobacteria	Actinobacteria	*Streptomyces lusitanus*	18
**DS56**	1358965-85-1	Grincamycin E	Bacteria	Actinobacteria	Actinobacteria	*Streptomyces lusitanus*	18
**DS57**	1358965-87-3	Grincamycin F	Bacteria	Actinobacteria	Actinobacteria	*Streptomyces lusitanus*	18
**DS58**	1380717-81-6	Spiroindimicin A	Bacteria	Actinobacteria	Actinobacteria	*Streptomyces* sp.	19
**DS59**	1380717-82-7	Spiroindimicin B	Bacteria	Actinobacteria	Actinobacteria	*Streptomyces* sp.	19
**DS60**	1380717-83-8	Spiroindimicin C	Bacteria	Actinobacteria	Actinobacteria	*Streptomyces* sp.	19
**DS61**	1380717-84-9	Spiroindimicin D	Bacteria	Actinobacteria	Actinobacteria	*Streptomyces* sp.	19
**DS62**	129029-32-9	Streptopyrrolidine	Bacteria	Actinobacteria	Actinobacteria	*Streptomyces* sp.	20
**DS63**	906095-21-4	Carboxamycin	Bacteria	Actinobacteria	Actinobacteria	*Streptomyces* sp.	21
**DS64**	1098023-63-2	Ammosamide A	Bacteria	Actinobacteria	Actinobacteria	*Streptomyces* sp.	22
**DS65**	1096365-01-3	Ammosamide B	Bacteria	Actinobacteria	Actinobacteria	*Streptomyces* sp.	22
**DS66**	1345002-07-4	Benzoxacystol	Bacteria	Actinobacteria	Actinobacteria	*Streptomyces* sp.	23
**DS70**	1414774-32-5	Luteoalbusin A	Fungi	Ascomycota	Sordariomycetes	*Acrostalagmus luteoalbus*	24
**DS71**	1414774-33-6	Luteoalbusin B	Fungi	Ascomycota	Sordariomycetes	*Acrostalagmus luteoalbus*	24
**DS72**	1363394-27-7	Waikialoid A	Fungi	Ascomycota	Eurotiomycetes	*Aspergillus* sp.	25
**DS73**	1363394-28-8	Waikialide A	Fungi	Ascomycota	Eurotiomycetes	*Aspergillus* sp.	25
**DS74**	1344087-42-8	Oxisterigmatocystin A	Fungi	Ascomycota	Eurotiomycetes	*Aspergillus versicolor*	26
**DS75**	1344087-43-9	Oxisterigmatocystin B	Fungi	Ascomycota	Eurotiomycetes	*Aspergillus versicolor*	26
**DS76**	1344087-44-0	Oxisterigmatocystin C	Fungi	Ascomycota	Eurotiomycetes	*Aspergillus versicolor*	26
**DS77**	1433960-41-8	Circumdatin K	Fungi	Ascomycota	Eurotiomycetes	*Aspergillus westerdijkiae*	27

**DS78**	1433960-42-9	Circumdatin L	Fungi	Ascomycota	Eurotiomycetes	*Aspergillus westerdijkiae*	27
**DS79**	1431865-15-4	5-Chlorosclerotiamide	Fungi	Ascomycota	Eurotiomycetes	*Aspergillus westerdijkiae*	27
**DS80**	1433960-43-0	10-*epi*-Sclerotiamide	Fungi	Ascomycota	Eurotiomycetes	*Aspergillus westerdijkiae*	27
**DS81**	1433960-44-1	Aspergilliamide B	Fungi	Ascomycota	Eurotiomycetes	*Aspergillus westerdijkiae*	27
**DS82**	1403482-26-7	Penilactone A	Fungi	Ascomycota	Eurotiomycetes	*Penicillium crustosum*	28
**DS83**	1403482-27-8	Penilactone B	Fungi	Ascomycota	Eurotiomycetes	*Penicillium crustosum*	28
**DS84**	1637437-89-8	Penipanoid A	Fungi	Ascomycota	Eurotiomycetes	*Penicillium paneum*	29
**DS85**	Not assigned	Penipanoid B	Fungi	Ascomycota	Eurotiomycetes	*Penicillium paneum*	29
**DS86**	1442381-95-4	Penipanoid C	Fungi	Ascomycota	Eurotiomycetes	*Penicillium paneum*	29
**DS87**	1334495-41-8	Berkeleyone A	Fungi	Ascomycota	Eurotiomycetes	*Penicillium rubrum*	30
**DS88**	1334495-42-9	Berkeleyone B	Fungi	Ascomycota	Eurotiomycetes	*Penicillium rubrum*	30
**DS89**	1334495-43-0	Berkeleyone C	Fungi	Ascomycota	Eurotiomycetes	*Penicillium rubrum*	30
**DS90**	1145681-71-5	Brevione F	Fungi	Ascomycota	Eurotiomycetes	*Penicillium* sp.	31
**DS91**	1145681-72-6	Brevione G	Fungi	Ascomycota	Eurotiomycetes	*Penicillium* sp.	31
**DS92**	1145681-73-7	Brevione H	Fungi	Ascomycota	Eurotiomycetes	*Penicillium* sp.	31
**DS93**	1422517-77-8	Sterolic acid	Fungi	Ascomycota	Eurotiomycetes	*Penicillium* sp.	31
**DS94**	1422517-78-9	Brevione I	Fungi	Ascomycota	Eurotiomycetes	*Penicillium* sp.	32
**DS95**	1422517-79-0	Brevione J	Fungi	Ascomycota	Eurotiomycetes	*Penicillium* sp.	32
**DS96**	1422517-80-3	Brevione K	Fungi	Ascomycota	Eurotiomycetes	*Penicillium* sp.	32
**DS97**	1393131-81-1	Panicilliumin A	Fungi	Ascomycota	Eurotiomycetes	*Penicillium* sp.	33
**DS98**	1311204-67-7	Dihydrotrichodermolide	Fungi	Ascomycota	Leotiomycetes	*Phialocephala malorum*	34
**DS99**	1311204-68-8	Dihydrodemethylsorbicillin	Fungi	Ascomycota	Leotiomycetes	*Phialocephala malorum*	34
**DS100**	1311379-65-3	Phialofurone	Fungi	Ascomycota	Leotiomycetes	*Phialocephala malorum*	34
**DS101**	1206199-47-4	Trichoderone	Fungi	Ascomycota	Sordariomycetes	*Trichoderma* sp.	35

**DS102**	1266399-63-6	Bathmodiolamide A	Animalia	Mollusca	Bivalvia	*Bathymodiolus thermophilus*	36
**DS103**	1266399-64-7	Bathmodiolamide B	Animalia	Mollusca	Bivalvia	*Bathymodiolus thermophilus*	36
**DS104**	1058645-82-1	Bistellettazine A	Animalia	Porifera	Demospongiae	*Stelletta* sp.	37
**DS105**	1058645-83-2	Bistellettazine B	Animalia	Porifera	Demospongiae	*Stelletta* sp.	37
**DS106**	1058645-84-3	Bistellettazine C	Animalia	Porifera	Demospongiae	*Stelletta* sp.	37
**DS108**	1265632-55-0	Franklinolide A	Animalia	Porifera	Demospongiae	*Geodia* sp.	38
**DS109**	1265194-73-7	Franklinolide A methyl ester	Animalia	Porifera	Demospongiae	*Geodia* sp.	38
**DS110**	1265632-60-7	Franklinolide B	Animalia	Porifera	Demospongiae	*Geodia* sp.	38
**DS111**	1265632-62-9	Franklinolide C	Animalia	Porifera	Demospongiae	*Geodia* sp.	38
**DS112**	1038397-16-8	Aplysinoplide A	Animalia	Porifera	Demospongiae	*Aplysinopsis digitata*	39
**DS113**	1038397-17-9	Aplysinoplide B	Animalia	Porifera	Demospongiae	*Aplysinopsis digitata*	39
**DS114**	1038397-18-0	Aplysinoplide C	Animalia	Porifera	Demospongiae	*Aplysinopsis digitata*	39
**DS115**	1245782-79-9	Heterofibrin A1	Animalia	Porifera	Demospongiae	*Spongia* sp.	40
**DS116**	1245782-80-2	Heterofibrin A2	Animalia	Porifera	Demospongiae	*Spongia* sp.	40
**DS117**	1245782-82-4	Heterofibrin A3	Animalia	Porifera	Demospongiae	*Spongia* sp.	40
**DS118**	1242136-13-5	Heterofibrin B1	Animalia	Porifera	Demospongiae	*Spongia* sp.	40
**DS119**	1245782-83-5	Heterofibrin B2	Animalia	Porifera	Demospongiae	*Spongia* sp.	40
**DS120**	1245782-84-6	Heterofibrin B3	Animalia	Porifera	Demospongiae	*Spongia* sp.	40
**DS121**	1296884-63-3	Fascioquinol A	Animalia	Porifera	Demospongiae	*Fasciospongia* sp.	41
**DS122**	1296884-64-4	Fascioquinol B	Animalia	Porifera	Demospongiae	*Fasciospongia* sp.	41
**DS123**	1296884-65-5	Fascioquinol C	Animalia	Porifera	Demospongiae	*Fasciospongia* sp.	41
**DS124**	1296884-66-6	Fascioquinol D	Animalia	Porifera	Demospongiae	*Fasciospongia* sp.	41
**DS125**	648883-30-1	Strongylophorine-22	Animalia	Porifera	Demospongiae	*Fasciospongia* sp.	41
**DS126**	1296884-67-7	Fascioquinol E	Animalia	Porifera	Demospongiae	*Fasciospongia* sp.	41
**DS127**	123086-41-9	Fascioquinol F	Animalia	Porifera	Demospongiae	*Fasciospongia* sp.	41
**DS128**	1384178-01-1	14-O-Sulfate massadine	Animalia	Porifera	Demospongiae	*Axinella* sp.	42
**DS129**	1384178-02-2	14-O-Methyl massadine	Animalia	Porifera	Demospongiae	*Axinella* sp.	42
**DS130**	1384178-03-3	3-O-Methyl massadine chloride	Animalia	Porifera	Demospongiae	*Axinella* sp.	42

**DS131**	1354782-07-2	Theonellin isocyanate	Animalia	Porifera	Demospongiae	*Rhaphoxya* sp.	43
**DS132**	1354782-08-3	Psammaplysin I	Animalia	Porifera	Demospongiae	*Rhaphoxya* sp.	43
**DS133**	1354782-09-4	Psammaplysin J	Animalia	Porifera	Demospongiae	*Rhaphoxya* sp.	43
**DS134**	1350449-55-6	Neopetrosiquinone A	Animalia	Porifera	Demospongiae	*Neopetrosia proxima*	44
**DS135**	1350449-56-7	Neopetrosiquinone B	Animalia	Porifera	Demospongiae	*Neopetrosia proxima*	44
**DS136**	1007592-39-3	(-)-Duryne	Animalia	Porifera	Demospongiae	*Petrosia* sp.	45
**DS137**	1304776-83-7	(-)-Duryne B	Animalia	Porifera	Demospongiae	*Petrosia* sp.	45
**DS138**	1304776-84-8	(-)-Duryne C	Animalia	Porifera	Demospongiae	*Petrosia* sp.	45
**DS139**	1304776-85-9	(-)-Duryne D	Animalia	Porifera	Demospongiae	*Petrosia* sp.	45
**DS140**	1304776-86-0	(-)-Duryne E	Animalia	Porifera	Demospongiae	*Petrosia* sp.	45
**DS141**	1304776-87-1	(-)-Duryne F	Animalia	Porifera	Demospongiae	*Petrosia* sp.	45
**DS146**	1246037-12-6	23-*nor*-Spiculoic acid	Animalia	Porifera	Demospongiae	*Plakortis angulospiculatus*	46
**DS147**	1246037-14-8	Zyggomphic acid B	Animalia	Porifera	Demospongiae	*Plakortis angulospiculatus*	46
**DS148**	1246037-17-1	27-*no*r-Zyggomphic acid B	Animalia	Porifera	Demospongiae	*Plakortis angulospiculatus*	46
**DS149**	1246037-19-3	22-*nor*-Zyggomphic acid B	Animalia	Porifera	Demospongiae	*Plakortis angulospiculatus*	46
**DS150**	1246037-22-8	22,27-*dinor*-Zyggomphic acid B	Animalia	Porifera	Demospongiae	*Plakortis angulospiculatus*	46
**DS151**	1246037-24-0	4,8,10,13-Tetradecatetraene-3,7-diol, 4,6,8,10,12-pentamethyl-14-phenyl-, (4*E*,8*E*,10*E*,13*E*)-(+)-	Animalia	Porifera	Demospongiae	*Plakortis halichondrioides*	46
**DS152**	1246037-25-1	3,6,8,11-Dodecatetraene-2,5-dione, 4,6,8,10-tetramethyl-12-phenyl-, (3*Z*,6*E*,8*E*,11*E*)-(+)-	Animalia	Porifera	Demospongiae	*Plakortis halichondrioides*	46
**DS153**	1246037-27-3	4,7-Octadien-2-one, 4,6-dimethyl-8-phenyl-, (4*E*,7*E*)-(+)-	Animalia	Porifera	Demospongiae	*Plakortis halichondrioides*	46
**DS154**	1174171-20-0	1,2-Dioxane-3-acetic acid, 3-hydroxy-4,6-dimethyl-6-[(9*E*)-2-	Animalia	Porifera	Demospongiae	*Plakortis* sp.	47

		methyl-10-phenyl-9-decen-1-yl]-, (3*R*,4*S*,6*R*)-rel-(+)-					
**DS155**	1174171-23-3	1,2-Dioxane-3-acetic acid, 3-hydroxy-4,6-dimethyl-6-(2-methyl-10-phenyldecyl)-, (3*R*,4*S*, 6*R*)-rel-(+)-	Animalia	Porifera	Demospongiae	*Plakortis* sp.	47
**DS156**	1174171-24-4	1,2-Dioxan-3-ol, 3,4,6-trimethyl-6-[(9*E*)-2-methyl-10-phenyl-9-decen-1-yl]-, (3*R*,4*S*,6*R*)-rel-(+)-	Animalia	Porifera	Demospongiae	*Plakortis* sp.	47
**DS157**	1174171-25-5	1,2-Dioxan-3-ol, 3,4,6-trimethyl-6-(2-methyl-10-phenyldecyl)-, (3*R*,4*S*,6*R*)-rel-(+)-	Animalia	Porifera	Demospongiae	*Plakortis* sp.	47
**DS158**	1174171-26-6	11-Dodecen-2-one, 4-methyl-12-phenyl-, (11*E*)-(-)-	Animalia	Porifera	Demospongiae	*Plakortis* sp.	47
**DS159**	1174171-27-7	2-Dodecanone, 4-methyl-12-phenyl-, (-)-	Animalia	Porifera	Demospongiae	*Plakortis* sp.	47
**DS160**	1006046-07-6	Leiodermatolide	Animalia	Porifera	Demospongiae	*Leiodermatium* sp.	48
**DS161**	1207066-37-2	Mutremdamide A	Animalia	Porifera	Demospongiae	*Theonella swinhoei*	49
**DS162**	1227466-12-7	Koshikamide C	Animalia	Porifera	Demospongiae	*Theonella cupola*	49
**DS163**	1227466-13-8	Koshikamide D	Animalia	Porifera	Demospongiae	*Theonella cupola*	49
**DS164**	1227466-14-9	Koshikamide E	Animalia	Porifera	Demospongiae	*Theonella cupola*	49
**DS165**	1227466-15-0	Koshikamide F	Animalia	Porifera	Demospongiae	*Theonella cupola*	49
**DS166**	1227466-16-1	Koshikamide G	Animalia	Porifera	Demospongiae	*Theonella cupola*	49
**DS167**	1227466-17-2	Koshikamide H	Animalia	Porifera	Demospongiae	*Theonella cupola*	49
**DS168**	1207875-34-0	Paltolide A	Animalia	Porifera	Demospongiae	*Theonella swinhoei*	50
**DS169**	1207875-35-1	Paltolide B	Animalia	Porifera	Demospongiae	*Theonella swinhoei*	50
**DS170**	1207875-36-2	Paltolide C	Animalia	Porifera	Demospongiae	*Theonella swinhoei*	50
**DS171**	1210424-24-0	Mirabilin H	Animalia	Porifera	Demospongiae	*Clathria* sp.	51
**DS172**	1210424-26-2	Mirabilin I	Animalia	Porifera	Demospongiae	*Clathria* sp.	51
**DS173**	1210424-29-5	Mirabilin J	Animalia	Porifera	Demospongiae	*Clathria* sp.	51
**DS174**	1330169-59-9	Citharoxazole	Animalia	Porifera	Demospongiae	*Latrunculia* sp.	52

**DS175**	1187966-70-6	Dihydrodiscorhabdin B	Animalia	Porifera	Demospongiae	*Latrunculia* sp.	53
**DS176**	1187966-71-7	Discorhabdin Y	Animalia	Porifera	Demospongiae	*Latrunculia* sp.	53
**DS177**	1156537-19-7	Halichondrin B-1140	Animalia	Porifera	Demospongiae	*Lissodendoryx* sp.	54
**DS178**	1156537-20-0	Halichondrin B-1092	Animalia	Porifera	Demospongiae	*Lissodendoryx* sp.	54
**DS179**	1156537-21-1	Halichondrin B-1020	Animalia	Porifera	Demospongiae	*Lissodendoryx* sp.	54
**DS180**	1155874-81-9	Halichondrin B-1076	Animalia	Porifera	Demospongiae	*Lissodendoryx* sp.	54
**DS181**	1023993-33-0	Phorbasin D	Animalia	Porifera	Demospongiae	*Phorbas* sp.	55
**DS182**	1023993-34-1	Phorbasin E	Animalia	Porifera	Demospongiae	*Phorbas* sp.	55
**DS183**	1023993-35-2	Phorbasin F	Animalia	Porifera	Demospongiae	*Phorbas* sp.	55
**DS184**	1082509-07-6	Phorbasin G	Animalia	Porifera	Demospongiae	*Phorbas* sp.	56
**DS185**	1082509-08-7	Phorbasin H	Animalia	Porifera	Demospongiae	*Phorbas* sp.	56
**DS186**	1082509-09-8	Phorbasin I	Animalia	Porifera	Demospongiae	*Phorbas* sp.	56
**DS187**	1082509-10-1	Phorbasin J	Animalia	Porifera	Demospongiae	*Phorbas* sp.	56
**DS188**	1082509-11-2	Phorbasin K	Animalia	Porifera	Demospongiae	*Phorbas* sp.	56

Table S2: Results of the SEA (Similarity Ensemble Approach) analysis conducted on each of the *drug-like* compounds in this study to identify possible enzyme targets.[57]

Compound	Target Key	Target Name	Description	p-value	MaxTC
Citharoxazole			No significant hits		
**DS1**	FOXO1_HUMAN+5	FOXO1	Forkhead box protein O1	5.09E-35	0.35
	5HT2C_RAT+5	Htr2c	5-hydroxytryptamine receptor 2C	3.18E-11	0.38
	NMDE1_HUMAN+5	GRIN2A	Glutamate receptor ionotropic, NMDA 2A	5.60E-11	0.3
	5HT1D_CAVPO+5	HTR1D	5-hydroxytryptamine receptor 1D	6.43E-10	0.29
	O61059_LEIME+5	LmGT2	Glucose transporter	1.03E-09	0.36
**DS6**	Q9GRG7_9TRYP+5	g6pd	Glucose-6-phosphate 1-dehydrogenase	1.19E-19	0.3
	GLI2_HUMAN+5	GLI2	Zinc finger protein GLI2	2.00E-15	0.42
	XBP1_RAT+5	Xbp1	X-box-binding protein 1	1.59E-11	0.31
	GLI1_HUMAN+5	GLI1	Zinc finger protein GLI1	1.39E-09	0.42
	MRP4_HUMAN+5	ABCC4	Multidrug resistance-associated protein 4	3.32E-08	0.29
**DS7**	MYB_CHICK+5	MYB	Transcriptional activator Myb	1.98E-11	0.36
	GLI2_HUMAN+5	GLI2	Zinc finger protein GLI2	6.37E-11	0.29
	XBP1_RAT+5	Xbp1	X-box-binding protein 1	7.88E-11	0.29
	GLI1_HUMAN+5	GLI1	Zinc finger protein GLI1	7.19E-07	0.29
**DS8**			No significant hits		
**DS9**	PA24A_BOVIN+5	PLA2G4A	Cytosolic phospholipase A2	4.31E-18	0.29
	GBRA1_MOUSE+5	Gabra1	Gamma-aminobutyric acid receptor subunit alpha-1	5.55E-16	0.31
	FABP5_HUMAN+5	FABP5	Fatty acid-binding protein, epidermal	2.54E-11	0.31
**DS10**			No significant hits		

**DS11**			No significant hits		
**DS29**	PARP1_RAT+5	Parp1	Poly [ADP-ribose] polymerase 1	3.26E-42	0.36
	PARP2_HUMAN+5	PARP2	Poly [ADP-ribose] polymerase 2	7.34E-28	0.32
	PARP1_HUMAN+5	PARP1	Poly [ADP-ribose] polymerase 1	2.89E-21	0.35
**DS30**	PAR10_HUMAN+5	PARP10	Poly [ADP-ribose] polymerase 10	7.07E-21	0.3
	S22A7_RAT+5	Slc22a7	Solute carrier family 22 member 7	3.20E-20	0.31
	PAR14_HUMAN+5	PARP14	Poly [ADP-ribose] polymerase 14	3.21E-19	0.29
	Q9K2N0_PSEAI+5	blaVIM-2	Beta-lactamase class B VIM-2	1.22E-15	0.31
	PAR15_HUMAN+5	PARP15	Poly [ADP-ribose] polymerase 15	2.44E-15	0.3
**DS31**	Q9K2N0_PSEAI+5	blaVIM-2	Beta-lactamase class B VIM-2	8.97E-33	0.35
	COX1_SHEEP+5	MT-CO1	Cytochrome c oxidase subunit 1	8.14E-29	0.29
	S22A7_RAT+5	Slc22a7	Solute carrier family 22 member 7	1.53E-19	0.3
	PAR14_HUMAN+5	PARP14	Poly [ADP-ribose] polymerase 14	1.31E-18	0.28
	COX2_SHEEP+5	MT-CO2	Cytochrome c oxidase subunit 2	1.78E-15	0.29
**DS32**			No significant hits		
**DS33**	PARP1_RAT+5	Parp1	Poly [ADP-ribose] polymerase 1	8.86E-97	0.39
	PARP1_HUMAN+5	PARP1	Poly [ADP-ribose] polymerase 1	2.93E-60	0.44
	PARP2_HUMAN+5	PARP2	Poly [ADP-ribose] polymerase 2	4.29E-46	0.37
	CD38_HUMAN+5	CD38	ADP-ribosyl cyclase/cyclic ADP-ribose hydrolase 1	2.22E-16	0.3
	PYRD_HUMAN+5	DHODH	Dihydroorotate dehydrogenase (quinone), mitochondrial	4.40E-13	0.41
**DS34**	PARP1_RAT+5	Parp1	Poly [ADP-ribose] polymerase 1	1.61E-78	0.37
	PARP1_HUMAN+5	PARP1	Poly [ADP-ribose] polymerase 1	4.94E-46	0.41
	PARP2_HUMAN+5	PARP2	Poly [ADP-ribose] polymerase 2	2.07E-37	0.37
	GRM7_RAT+5	Grm7	Metabotropic glutamate receptor 7	1.11E-16	0.31
	KS6B1_HUMAN+5	RPS6KB1	Ribosomal protein S6 kinase beta-1	1.55E-15	0.34
**DS36**	FPRS1_MOUSE+5	Fpr-s1	Formyl peptide receptor-related sequence 1	1.21E-72	0.33
	EDNRB_RAT+5	Ednrb	Endothelin B receptor	8.35E-58	0.33

	ECE1_BOVIN+5	ECE1	Endothelin-converting enzyme 1	3.57E-38	0.31
	CP51_TRYCC+5	CYP51	Sterol 14-alpha demethylase	1.81E-33	0.33
	RUVB1_HUMAN+5	RUVBL1	RuvB-like 1	1.81E-33	0.33
**DS37**	MTR1A_HUMAN+5	MTNR1A	Melatonin receptor type 1A	2.11E-110	0.46
	MTR1B_HUMAN+5	MTNR1B	Melatonin receptor type 1B	4.72E-97	0.41
	SMS1_HUMAN+5	SGMS1	Phosphatidylcholine:ceramide cholinephosphotransferase 1	2.51E-49	0.38
	MTR1C_XENLA+5	mtnr1c	Melatonin receptor type 1C	1.28E-44	0.38
	NU1M_BOVIN+5	MT-ND1	NADH-ubiquinone oxidoreductase chain 1	1.41E-33	0.33
**DS38**	PA_I000X+5	PA	Polymerase acidic protein	1.01E-32	0.38
	GNRHR_RAT+5	Gnrhr	Gonadotropin-releasing hormone receptor	2.90E-27	0.41
	SC6A5_HUMAN+5	SLC6A5	Sodium- and chloride-dependent glycine transporter 2	8.33E-26	0.34
	BRS3_HUMAN+5	BRS3	Bombesin receptor subtype-3	2.74E-24	0.35
	GBRA1_RAT+5	Gabra1	Gamma-aminobutyric acid receptor subunit alpha-1	6.44E-20	0.32
**DS39**	BRS3_HUMAN+5	BRS3	Bombesin receptor subtype-3	2.81E-31	0.4
	TAAR1_HUMAN+5	TAAR1	Trace amine-associated receptor 1	5.02E-30	0.42
	STAT6_MOUSE+5	Stat6	Signal transducer and transcription activator 6	1.21E-28	0.28
	GBRA6_HUMAN+5	GABRA6	Gamma-aminobutyric acid receptor subunit alpha-6	3.98E-27	0.35
	MTR1A_HUMAN+5	MTNR1A	Melatonin receptor type 1A	5.67E-24	0.43
**DS40**	GRPR_MOUSE+5	Grpr	Gastrin-releasing peptide receptor	5.83E-87	0.32
	MSHR_MOUSE+5	Mc1r	Melanocyte-stimulating hormone receptor	1.20E-75	0.38
	ECE1_BOVIN+5	ECE1	Endothelin-converting enzyme 1	2.78E-60	0.33
	FPRS1_MOUSE+5	Fpr-s1	Formyl peptide receptor-related sequence 1	2.33E-54	0.29
	MTR1C_XENLA+5	mtnr1c	Melatonin receptor type 1C	1.71E-42	0.41
**DS41**	GRP1_RAT+5	Rasgrp1	RAS guanyl-releasing protein 1	1.13E-44	0.41
	GALR1_RAT+5	Galr1	Galanin receptor type 1	5.02E-41	0.33
	GALR2_RAT+5	Galr2	Galanin receptor type 2	5.02E-41	0.33
	KPCD_MOUSE+5	Prkcd	Protein kinase C delta type	1.64E-30	0.41

	KPCA_BOVIN+5	PRKCA	Protein kinase C alpha type	2.63E-20	0.31
**DS47**			No significant hits		
**DS48**	EHMT2_MOUSE+5	Ehmt2	Histone-lysine N-methyltransferase EHMT2	2.36E-31	0.31
	SUV39_DROME+5	Su(var)3-9	Histone-lysine N-methyltransferase Su(var)3-9	2.36E-31	0.31
	SUV91_HUMAN+5	SUV39H1	Histone-lysine N-methyltransferase SUV39H1	7.26E-19	0.33
	TRXR1_HUMAN+5	TXNRD1	Thioredoxin reductase 1, cytoplasmic	1.86E-10	0.31
**DS49**			No significant hits		
**DS50**			No significant hits		
**DS58**			No significant hits		
**DS60**	HST2_YEAST+5	HST2	NAD-dependent protein deacetylase HST2	2.11E-15	0.3
	NR1H3_HUMAN+5	NR1H3	Oxysterols receptor LXR-alpha	4.59E-08	0.33
	NR1H2_HUMAN+5	NR1H2	Oxysterols receptor LXR-beta	9.79E-07	0.33
	E2AK2_HUMAN+5	EIF2AK2	Interferon-induced, double-stranded RNA-activated protein kinase	2.68E-06	0.3
	BAZ2B_HUMAN+5	BAZ2B	Bromodomain adjacent to zinc finger domain protein 2B	3.18E-06	0.29
**DS62**	Q72874_9HIV1+5	pol	Pol polyprotein	2.56E-60	0.61
	GALR1_RAT+5	Galr1	Galanin receptor type 1	1.86E-52	0.36
	GALR2_RAT+5	Galr2	Galanin receptor type 2	1.86E-52	0.36
	ISPT_STRPN+5	uppS	Isoprenyl transferase	2.21E-47	0.37
	NMDE2_RAT+5	Grin2b	Glutamate receptor ionotropic, NMDA 2B	1.13E-42	0.34
**DS63**	Q9K2N0_PSEAI+5	blaVIM-2	Beta-lactamase class B VIM-2	1.82E-30	0.33
	TCMO_HELTU+5	CYP73A1	Trans-cinnamate 4-monooxygenase	9.10E-30	0.3
	Q8S3J0_BROTE+5	ALS	Acetolactate synthase	8.14E-29	0.29
	COX1_SHEEP+5	MT-CO1	Cytochrome c oxidase subunit 1	1.39E-28	0.28
	Q8T8E9_9TRYP+5	galE	UDP-galactose 4-epimerase	1.61E-27	0.34
**DS64**	NQO2_HUMAN+5	NQO2	Ribosyldihydronicotinamide dehydrogenase [quinone]	4.12E-12	0.76

**DS65**	NQO2_HUMAN+5	NQO2	Ribosyldihydronicotinamide dehydrogenase [quinone]	2.78E-15	1
**DS70**	EHMT2_MOUSE+5	Ehmt2	Histone-lysine N-methyltransferase EHMT2	1.16E-68	0.69
	SUV39_DROME+5	Su(var)3-9	Histone-lysine N-methyltransferase Su(var)3-9	1.16E-68	0.69
	SUV91_HUMAN+5	SUV39H1	Histone-lysine N-methyltransferase SUV39H1	1.13E-61	0.69
	TRXR1_HUMAN+5	TXNRD1	Thioredoxin reductase 1, cytoplasmic	2.99E-22	0.69
	EHMT2_HUMAN+5	EHMT2	Histone-lysine N-methyltransferase EHMT2	1.02E-19	0.69
**DS71**	EHMT2_MOUSE+5	Ehmt2	Histone-lysine N-methyltransferase EHMT2	1.35E-61	0.62
	SUV39_DROME+5	Su(var)3-9	Histone-lysine N-methyltransferase Su(var)3-9	1.35E-61	0.62
	SUV91_HUMAN+5	SUV39H1	Histone-lysine N-methyltransferase SUV39H1	7.91E-56	0.62
	TRXR1_HUMAN+5	TXNRD1	Thioredoxin reductase 1, cytoplasmic	5.12E-20	0.62
	EHMT2_HUMAN+5	EHMT2	Histone-lysine N-methyltransferase EHMT2	1.01E-17	0.62
**DS73**			No significant hits		
**DS74**	CP1B1_HUMAN+5	CYP1B1	Cytochrome P450 1B1	2.69E-51	0.35
	T2R31_HUMAN+5	TAS2R31	Taste receptor type 2 member 31	5.83E-38	0.31
	HIF1A_MOUSE+5	Hif1a	Hypoxia-inducible factor 1-alpha	6.01E-30	0.3
	Q3I4V7_CRYNV+5	CAN2	Carbonic anhydrase	2.17E-14	0.32
	ABCG2_HUMAN+5	ABCG2	ATP-binding cassette sub-family G member 2	4.86E-13	0.35
**DS75**	CP1B1_HUMAN+5	CYP1B1	Cytochrome P450 1B1	2.69E-51	0.35
	T2R31_HUMAN+5	TAS2R31	Taste receptor type 2 member 31	5.83E-38	0.31
	HIF1A_MOUSE+5	Hif1a	Hypoxia-inducible factor 1-alpha	6.01E-30	0.3
	Q3I4V7_CRYNV+5	CAN2	Carbonic anhydrase	2.17E-14	0.32
	ABCG2_HUMAN+5	ABCG2	ATP-binding cassette sub-family G member 2	4.86E-13	0.35
**DS76**	CP1B1_HUMAN+5	CYP1B1	Cytochrome P450 1B1	5.33E-38	0.33
	T2R31_HUMAN+5	TAS2R31	Taste receptor type 2 member 31	2.75E-37	0.31
	ABCG2_HUMAN+5	ABCG2	ATP-binding cassette sub-family G member 2	1.11E-16	0.39
	MDR1A_MOUSE+5	Abcb1a	Multidrug resistance protein 1A	2.25E-11	0.34
	ALDR_RAT+5	Akr1b1	Aldose reductase	3.10E-11	0.36
**DS77**	5HT6R_MOUSE+5	Htr6	5-hydroxytryptamine receptor 6	1.26E-18	0.28

	PARP1_MOUSE+5	Parp1	Poly [ADP-ribose] polymerase 1	1.01E-13	0.37
	HIF1A_HUMAN+5	HIF1A	Hypoxia-inducible factor 1-alpha	8.38E-12	0.31
	HPPD_HUMAN+5	HPD	4-hydroxyphenylpyruvate dioxygenase	7.45E-11	0.3
	FTO_HUMAN+5	FTO	Alpha-ketoglutarate-dependent dioxygenase FTO	1.14E-10	0.28
**DS78**			No significant hits		
**DS79**			No significant hits		
**DS80**	DCOR_HUMAN+5	ODC1	Ornithine decarboxylase	6.50E-07	0.31
**DS81**	KPCA_BOVIN+5	PRKCA	Protein kinase C alpha type	1.25E-24	0.41
**DS85**	ACM2_RAT+5	Chrm2	Muscarinic acetylcholine receptor M2	9.92E-11	0.33
	TNKS2_HUMAN+5	TNKS2	Tankyrase-2	5.51E-10	0.31
	GALK1_HUMAN+5	GALK1	Galactokinase	5.33E-09	0.29
	ACM1_RAT+5	Chrm1	Muscarinic acetylcholine receptor M1	4.14E-07	0.33
**DS86**	CAH3_BOVIN+5	CA3	Carbonic anhydrase 3	2.77E-44	0.35
	GLI2_HUMAN+5	GLI2	Zinc finger protein GLI2	4.61E-35	0.36
	PDE10_HUMAN+5	PDE10A	cAMP and cAMP-inhibited cGMP 3',5'-cyclic phosphodiesterase 10A	1.41E-30	0.42
	SSDH_HUMAN+5	ALDH5A1	Succinate-semialdehyde dehydrogenase, mitochondrial	8.14E-29	0.29
	GRM5_MOUSE+5	Grm5	Metabotropic glutamate receptor 5	2.41E-25	0.31
**DS87**	IL1B_HUMAN+5	IL1B	Interleukin-1 beta	2.94E-78	1
	CD81_HUMAN+5	CD81	CD81 antigen	3.50E-36	0.36
	DHI2_RAT+5	Hsd11b2	Corticosteroid 11-beta-dehydrogenase isozyme 2	9.02E-35	0.35
	DPOLB_RAT+5	Polb	DNA polymerase beta	1.75E-27	0.37
	PA2A1_ECHCA+5		Acidic phospholipase A2 EC-I	1.83E-21	0.33
**DS88**	IL1B_HUMAN+5	IL1B	Interleukin-1 beta	3.65E-84	1
**DS89**	IL1B_HUMAN+5	IL1B	Interleukin-1 beta	4.62E-62	0.55
**DS90**			No significant hits		
**DS91**			No significant hits		
**DS94**			No significant hits		

**DS95**			No significant hits		
**DS96**			No significant hits		
**DS97**	T2R31_HUMAN+5	TAS2R31	Taste receptor type 2 member 31	1.88E-12	0.29
	ACEA_CANAX+5	ICL1	Isocitrate lyase	1.47E-11	0.31
**DS98**			No significant hits		
**DS99**	MGRA_STAAU+5	mgrA	HTH-type transcriptional regulator MgrA	8.14E-29	0.29
	GRM3_RAT+5	Grm3	Metabotropic glutamate receptor 3	1.11E-16	0.34
	GRM2_RAT+5	Grm2	Metabotropic glutamate receptor 2	1.22E-15	0.35
	POL_RSVP+5	gag-pro-pol	Gag-Pro-Pol polyprotein	1.64E-14	0.28
	LIPP_PIG+5	PNLIP	Pancreatic triacylglycerol lipase	1.13E-08	0.28
**DS121**	S22A8_RAT+5	Slc22a8	Solute carrier family 22 member 8	1.11E-15	0.31
	ACEA_CANAX+5	ICL1	Isocitrate lyase	1.47E-14	0.4
	SO1A1_RAT+5	Slco1a1	Solute carrier organic anion transporter family member 1A1	2.43E-12	0.34
	MRP4_HUMAN+5	ABCC4	Multidrug resistance-associated protein 4	2.90E-09	0.33
	PA2_APIME+5		Phospholipase A2	5.01E-08	0.28
**DS131**			No significant hits		
**DS134**	PA2_APIME+5		Phospholipase A2	1.92E-10	0.38
**DS135**	PA2_APIME+5		Phospholipase A2	8.22E-09	0.31
**DS152**	AMD_HUMAN+5	PAM	Peptidyl-glycine alpha-amidating monooxygenase	1.78E-15	0.32
	FTSZ_ECOLI+5	ftsZ	Cell division protein FtsZ	2.65E-12	0.29
	O93874_COCLU+5	17HSDcl	17beta-hydroxysteroid dehydrogenase	1.80E-11	0.31
	RBBP9_HUMAN+5	RBBP9	Putative hydrolase RBBP9	5.83E-09	0.29
	P2RX1_MOUSE+5	P2rx1	P2X purinoceptor 1	3.93E-08	0.31
**DS153**	O93874_COCLU+5	17HSDcl	17beta-hydroxysteroid dehydrogenase	3.87E-44	0.36
	AMPB_MOUSE+5	Rnpep	Aminopeptidase B	2.14E-30	0.3
	AMPL_BOVIN+5	LAP3	Cytosol aminopeptidase	2.14E-30	0.3
	NEP_MOUSE+5	Mme	Neprilysin	2.62E-29	0.33

	ACER2_HUMAN+5	ACER2	Alkaline ceramidase 2	4.39E-29	0.29
**DS171**			No significant hits		
**DS172**			No significant hits		
**DS173**			No significant hits		
**DS174**			No significant hits		
**DS175**			No significant hits		
**DS176**			No significant hits		
**DS184**			No significant hits		
**DS185**			No significant hits		

References:

1. R. A. Davis, M. Sykes, V. M. Avery, D. Camp and R. J. Quinn, *Bioorg. Med. Chem.*, **2011**, *19*, 6615–6619.

2. D. R. Appleton, C. S. Chuen, M. V. Berridge, V. L. Webb and B. R. Copp, *J. Org. Chem.*, **2009**, *74*, 9195–9198.

3. S.-T. Ishigami, Y. Goto, N. Inoue, S.-I. Kawazu, Y. Matsumoto, Y. Imahara, M. Tarumi, H. Nakai, N. Fusetani and Y. Nakao, *J. Org. Chem.*, **2012**, *77*, 10962–10966.

4. M. T. R. de Almeida, G. E. Siless, C. D. Perez, M. J. Veloso, L. Schejter, L. Puricelli and J. A. Palermo, *J. Nat. Prod.*, **2010**, *73*, 1714–1717.

5. C. H. Gao, Y. F. Wang, S. Li, P. Y. Qian and S. H. Qi, *Mar. Drugs*, **2011**, *9*, 2479–2487.

6. H. V. K. Wangun, A. Wood, C. Fiorillo, J. K. Reed, P. J. McCarthy and A. E. Wrightt, *J. Nat. Prod.*, **2010**, *73*, 712–715.

7. K. Wolkenstein, W. Schoefberger, N. Muller and T. Oji, *J. Nat. Prod.*, **2009**, *72*, 2036–2039.

8. A. S. Antonov, S. A. Avilov, A. I. Kalinovsky, S. D. Anastyuk, P. S. Dmitrenok, V. I. Kalinin, S. Taboada, A. Bosh, C. Avila and V. A. Stonik, *J. Nat. Prod.*, **2009**, *72*, 33–38.

9. Y. Li, Y. Xu, L. Liu, Z. Han, P. Y. Lai, X. Guo, X. Zhang, W. Lin and P.-Y. Qian, *Mar. Drugs*, **2012**, *10*, 319–328.

10. W. M. Abdel-Mageed, B. F. Milne, M. Wagner, M. Schumacher, P. Sandor, W. Pathom-aree, M. Goodfellow, A. T. Bull, K. Horikoshi, R. Ebel, M. Diederich, H. P. Fiedler and M. Jaspars, *Org. Biomol. Chem.*, **2010**, *8*, 2352–2362.

11. L. Chen, X. X. Tang, M. Zheng, Z. W. Yi, X. Xiao, Y. K. Qiu and Z. Wu, *J. Asian Nat. Prod. Res.*, **2011**, *13*, 444–448.

12. H. B. Huang, Y. L. Yao, Z. X. He, T. T. Yang, J. Y. Ma, X. P. Tian, Y. Y. Li, C. G. Huang, X. P. Chen, W. J. Li, S. Zhang, C. S. Zhang and J. H. Ju, *J. Nat. Prod.*, **2011**, *74*, 2122–2127.

13. A. Gartner, B. Ohlendorf, D. Schulz, H. Zinecker, J. Wiese and J. F. Imhoff, *Mar. Drugs*, **2011**, *9*, 98–108.

14. R. Raju, A. M. Piggott, M. Conte, Z. Tnimov, K. Alexandrov and R. J. Capon, *Chem. – Eur. J.*, **2010**, *16*, 3194–3200.

15. R. Raju, A. M. Piggott, X.-C. Huang and R. J. Capon, *Org. Lett.*, **2011**, *13*, 2770–2773.

16. S. Li, X. Tian, S. Niu, W. Zhang, Y. Chen, H. Zhang, X. Yang, W. Zhang, W. Li, S. Zhang, J. Ju and C. Zhang, *Mar. Drugs*, **2011**, *9*, 1428–1439.

17. X.-W. Yang, G.-Y. Zhang, J.-X. Ying, B. Yang, X.-F. Zhou, A. Steinmetz, Y.-H. Liu and N. Wang, *Mar. Drugs*, **2012**, *11*, 33–39.

18. H. B. Huang, T. T. Yang, X. M. Ren, J. Liu, Y. X. Song, A. J. Sun, J. Y. Ma, B. Wang, Y. Zhang, C. G. Huang, C. S. Zhang and J. H. Ju, *J. Nat. Prod.*, **2012**, *75*, 202–208.

19. W. J. Zhang, Z. Liu, S. M. Li, T. T. Yang, Q. B. Zhang, L. Ma, X. P. Tian, H. B. Zhang, C. G. Huang, S. Zhang, J. H. Ju, Y. M. Shen and C. S. Zhang, *Org. Lett.*, **2012**, *14*, 3364 3367.

20. H. J. Shin, T. S. Kim, H. S. Lee, J. Y. Park, I. K. Choi and H. J. Kwon, *Phytochemistry*, **2008**, *69*, 2363–2366.

21. C. Hohmann, K. Schneider, C. Brunter, E. Irran, G. Nicholson, A. T. Bull, A. L. Jones, R. Brown, J. E. M. Stach, M. Goodfellow, W. Beil, M. Kr¨amer, J. F. Imhoff, R. D. S¨ussmuth and H.-P. Fiedler, *J. Antibiot.*, **2009**, *62*, 99–104.

22. S. Taboada, L. Nunez-Pons and C. Avila, *Polar Biol*., **2013**, *36*, 13–25.

23. J. Nachtigall, K. Schneider, C. Bruntner, A. T. Bull, M. Goodfellow, H. Zinecker, J. F. Imhoff, G. Nicholson, E. Irran, R. D. Sussmuth and H. P. Fiedler, *J. Antibiot.*, **2011**, *64*, 453–457.

24. F. Z. Wang, Z. Huang, X. F. Shi, Y. C. Chen, W. M. Zhang, X. P. Tian, J. Li and S. Zhang, *Bioorg. Med. Chem. Lett.*, **2012**, *22*, 7265–7267.

25. X. R. Wang, J. L. You, J. B. King, D. R. Powell and R. H. Cichewicz, *J. Nat. Prod.*, **2012**, *75*, 707–715.

26. S. X. Cai, T. J. Zhu, L. Du, B. Y. Zhao, D. H. Li and Q. Q. Gu, *J. Antibiot.*, **2011**, *64*, 193–196.

27. J. Peng, X. Y. Zhang, Z. C. Tu, X. Y. Xu and S. H. Qi, *J. Nat. Prod.,* **2013**, *76*, 983–987.

28. G. W. Wu, H. Y. Ma, T. J. Zhu, J. Li, Q. Q. Gu and D. H. Li, *Tetrahedron*, **2012**, *68*, 9745–9749.

29. C. S. Li, C. Y. An, X. M. Li, S. S. Gao, C. M. Cui, H. F. Sun and B. G. Wang, *J. Nat. Prod.*, **2011**, *74*, 1331–1334.

30. D. B. Stierle, A. A. Stierle, B. Patacini, K. McIntyre, T. Girtsman and E. Bolstad, *J. Nat. Prod.*, **2011**, *74*, 2273–2277.

31. Y. Li, D. Ye, Z. Shao, C. Cui and Y. Che, *Mar. Drugs*, **2012**, *10*, 497–508.

32. Y. Li, D. Z. Ye, X. L. Chen, X. H. Lu, Z. Z. Shao, H. Zhang and Y. S. Che, *J. Nat. Prod.*, **2009**, *72*, 912–916.

33. X. Lin, X. Zhou, F. Wang, K. Liu, B. Yang, X. Yang, Y. Peng, J. Liu, Z. Ren and Y. Liu, *Mar. Drugs*, **2012**, *10*, 106–115.

34. D. H. Li, S. X. Cai, T. J. Zhu, F. P. Wang, X. Xiao and Q. Q. Gu, *Chem. Biodiversity*, **2011**, *8*, 895–901.

35. J. L. You, H. Q. Dai, Z. H. Chen, G. J. Liu, Z. X. He, F. H. Song, X. Yang, H. A. Fu, L. X. Zhang and X. P. Chen, *J. Ind. Microbiol. Biotechnol.*, **2010**, *37*, 245–252.

36. E. H. Andrianasolo, L. Haramaty, K. L. McPhail, E. White, C. Vetriani, P. Falkowski and R. Lutz, *J. Nat. Prod.*, **2011**, *74*, 842–846.

37. M. El-Naggar, A. M. Piggott and R. J. Capon, *Org. Lett.*, **2008**, *10*, 4247–4250.

38. H. Zhang, M. M. Conte and R. J. Capon, *Angew. Chem., Int. Ed.*, **2010**, *49*, 9904–9906.

39. R. Ueoka, Y. Nakao, S. Fujii, R. W. M. van Soest and S. Matsunaga, *J. Nat. Prod.*, **2008**, *71*, 1089–1091.

40. A. A. Salim, J. Rae, F. Fontaine, M. M. Conte, Z. Khalil, S. Martin, R. G. Parton and R. J. Capon, *Org. Biomol. Chem.*, **2010**, *8*, 3188–3194.

41. H. Zhang, Z. G. Khalil and R. J. Capon, *Tetrahedron*, **2011**, *67*, 2591–2595.

42. H. Zhang, Z. Khalil, M. M. Conte, F. Plisson and R. J. Capon, *Tetrahedron Lett.*, **2012**, *53*, 3784–3787.

43. A. D. Wright, P. J. Schupp, J. P. Schror, A. Engemann, S. Rohde, D. Kelman, N. de Voogd, A. Carroll and C. A. Motti, *J. Nat. Prod.*, **2012**, *75*, 502–506.

44. P. L. Winder, H. L. Baker, P. Linley, E. A. Guzman, S. A. Pomponi, M. C. Diaz, J. K. Reed and A. E. Wright, *Bioorg. Med. Chem.*, **2011**, *19*, 6599–6603.

45. Y. Hitora, K. Takada, S. Okada, Y. Ise and S. Matsunaga, *J. Nat. Prod.*, **2011**, *74*, 1262–1267.

46. S. Ankisetty, D. J. Gochfeld, M. C. Diaz, S. I. Khan and M. Slattery, *J. Nat. Prod.*, **2010**, *73*, 1494–1498.

47. E. Manzo, M. L. Ciavatta, D. Melck, P. Schupp, N. J. de Voogd and M. Gavagnin, *J. Nat. Prod.*, **2009**, *72*, 1547–1551.

48. I. Paterson, S. M. Dalby, J. C. Roberts, G. J. Naylor, E. A. Guzman, R. Isbrucker, T. P. Pitts, P. Linley, D. Divlianska, J. K. Reed and A. E. Wright, *Angew. Chem., Int. Ed.*, **2011**, *50*, 3219–3223.

49. A. Plaza, G. Bifulco, M. Masullo, J. R. Lloyd, J. L. Keffer, P. L. Colin, J. N. A. Hooper, L. J. Bell and C. A. Bewley, *J. Org. Chem.*, **2010**, *75*, 4344–4355.

50. A. Plaza, J. L. Keffer, J. R. Lloyd, P. L. Colin and C. A. Bewley, *J. Nat. Prod.*, **2010**, *73*, 485–488.

51. M. El-Naggar, M. Conte and R. J. Capon, *Org. Biomol. Chem.*, **2010**, *8*, 407–412.

52. G. Genta-Jouve, N. Francezon, A. Puissant, P. Auberger, J. Vacelet, T. Perez, A. Fontana, A. Al Mourabit and O. P. Thomas, *Magn. Reson. Chem.*, **2011**, *49*, 533–536.

53. M. K. Na, Y. Q. Ding, B. Wang, B. L. Tekwani, R. F. Schinazi, S. Franzblau, M. Kelly, R. Stone, X. C. Li, D. Ferreira and M. T. Hamann, *J. Nat. Prod.*, **2010**, *73*, 383–387.

54. S. J. H. Hickford, J. W. Blunt and M. H. G. Munro, *Bioorg. Med. Chem.*, **2009**, *17*, 2199–2203.

55. H. Zhang and R. J. Capon, *Org. Lett.*, **2008**, *10*, 1959–1962.

56. H. Zhang, J. M. Major, R. J. Lewis and R. J. Capon, *Org. Biomol. Chem.*, **2008**, *6*, 3811–3815.

57. M. J. Keiser, B. L. Roth, B. N. Armbruster, P. Ernsberger, J. J. Irwin, B. K. Shoichet,. *Nat. Biotech.* **2007**, *25*, 197-206.
